# The associations of previous influenza/upper respiratory infection with COVID-19 susceptibility/morbidity/mortality: a nationwide cohort study in South Korea

**DOI:** 10.1038/s41598-021-00428-x

**Published:** 2021-11-03

**Authors:** So Young Kim, Joo-Hee Kim, Miyoung Kim, Jee Hye Wee, Younghee Jung, Chanyang Min, Dae Myoung Yoo, Songyong Sim, Hyo Geun Choi

**Affiliations:** 1 Department of Otorhinolaryngology-Head & Neck Surgery, CHA Bundang Medical Center, CHA University, Seongnam, Korea; 2 Division of Pulmonary, Allergy, and Critical Care Medicine, Department of Internal Medicine, Hallym University College of Medicine, Hallym University Sacred Heart Hospital, Anyang, Korea; 3 Department of Laboratory Medicine, Hallym University College of Medicine, Anyang, Korea; 4 Department of Otorhinolaryngology-Head & Neck Surgery, Hallym University College of Medicine, Anyang, Korea; 5 Division of Infectious Diseases, Department of Internal Medicine, Hallym University Sacred Heart Hospital, Hallym University College of Medicine, Anyang, Republic of Korea; 6 Hallym Data Science Laboratory, Hallym University College of Medicine, Anyang, Korea; 7 Graduate School of Public Health, Seoul National University, Seoul, Korea; 8 School of Data Science, Hallym University, Chuncheon, Korea

**Keywords:** Diseases, Medical research, Risk factors, Signs and symptoms

## Abstract

We aimed to investigate the associations of previous influenza/URI with the susceptibility of COVID-19 patients compared to that of non-COVID-19 participants. A nationwide COVID-19 cohort database was collected by the Korea National Health Insurance Corporation. A total of 8,070 COVID-19 patients (1 January 2020 through 4 June 2020) were matched with 32,280 control participants. Severe COVID-19 morbidity was defined based on the treatment histories of the intensive care unit, invasive ventilation, and extracorporeal membrane oxygenation and death. The susceptibility/morbidity/mortality associated with prior histories of 1–14, 1–30, 1–90, 15–45, 15–90, and 31–90 days before COVID-19 onset were analyzed using conditional/unconditional logistic regression. Prior influenza infection was related to increased susceptibility to COVID-19 (adjusted odds ratio [95% confidence interval] = 3.07 [1.61–5.85] for 1–14 days and 1.91 [1.54–2.37] for 1–90 days). Prior URI was also associated with increased susceptibility to COVID-19 (6.95 [6.38–7.58] for 1–14 days, 4.99 [4.64–5.37] for 1–30 days, and 2.70 [2.55–2.86] for 1–90 days). COVID-19 morbidity was positively associated with influenza (3.64 [1.55–9.21] and 3.59 [1.42–9.05]) and URI (1.40 [1.11–1.78] and 1.28 [1.02–1.61]) at 1–14 days and 1–30 days, respectively. Overall, previous influenza/URI did not show an association with COVID-19 mortality. Previous influenza/URI histories were associated with increased COVID-19 susceptibility and morbidity. Our findings indicate why controlling influenza/URI is important during the COVID-19 pandemic.

## Introduction

The coronavirus disease 19 (COVID-19) pandemic has lasted for approximately one year worldwide^1^. Another infectious viral disease, influenza, was approximated to have a mortality rate of 2.9–44.0 per 100,000 people/year^2^. In addition, elderly patients and patients with other medical morbidities are more susceptible to COVID-19 as well as influenza/URI^3,4^. Thus, there have been growing concerns about aggravated COVID-19 susceptibility and severity in patients with influenza or URI^5,6^.

Recent studies have suggested the effects of influenza infection on the disease course of COVID-19^7,8^. They reported severe COVID-19 outcomes in patients with coinfection with influenza^7–9^. Patients coinfected with SARS-CoV-2 and influenza were reportedly more prone to severe inflammation and organ injury, which could induce cytokine storms^7^. Coinfected patients demonstrated a higher risk of death, ventilator care, and intensive care unit (ICU) admission than influenza or SARS-CoV-2 single-infected patients^8^. In contrast, other studies reported a lower or similar rate of fatality of COVID-19 in coinfected patients with influenza than in single SARS-CoV-2-infected patients^10,11^. Moreover, the recent infection histories of endemic coronavirus infection were suggested to be associated with a milder clinical course of COVID-19^12^. The findings of this study suggested that the preexisting viral immune response might be effective in evading SARS-CoV-2 infection^12^. Owing to the limited number of study populations and unconcerned confounders, the impacts of influenza infection on COVID-19 are still controversial.

We hypothesized that recent infection histories of influenza/URI might be associated with SARS-CoV-2 positivity and COVID-19 morbidity/mortality. Because recent viral infections may transiently boost the immune response to another viral pathogen (SARS-CoV-2), vulnerability to viral infection could render certain patients highly susceptible to both SARS-CoV-2 and other viral infections^3,4^. Because URIs commonly originate from viral infections, such as rhinovirus, parainfluenza virus, adenovirus, coronavirus, and respiratory syncytial virus, we also analyzed URIs and influenza. Because patients with influenza infection/URI are not quarantined, the possibility of recent previous influenza/URI infection could exist. On the other hand, the opposite case might be rare because of quarantine in the case of COVID-19. Therefore, we analyzed the associations of previous influenza/URI with COVID-19. The primary object of this study was to evaluate the association between previous influenza/URI and susceptibility of COVID-19 patients compared to that of non-COVID-19 participants. The secondary objective was to estimate the relation between previous influenza/URI and morbidity/mortality in COVID-19 patients.

## Materials and methods

### Study population and participant selection

The ethics committee of Hallym University permitted this study (IRB: 2020–07-022) and waived written informed consent. All analyses adhered to the guidelines and regulations of the ethics committee of Hallym University.

We used Korea National Health Insurance Database Coronavirus Disease 2019 (NHID-COVID) medical claim code data from 2015 through 2020, which covers the entire country without any exception^13,14^. The NHID-COVID provided data of individuals who underwent SARS-CoV-2 testing using real-time reverse transcriptase–polymerase chain reaction (RT-PCR) assay of nasopharyngeal or pharyngeal swabs, in accordance with the WHO guideline, and control participants were proportionally sampled, stratifying with age and sex, from the NHID. A 15-fold number of control participants who were matched with COVID-19 patients for age and sex were provided.

**Figure 1:**
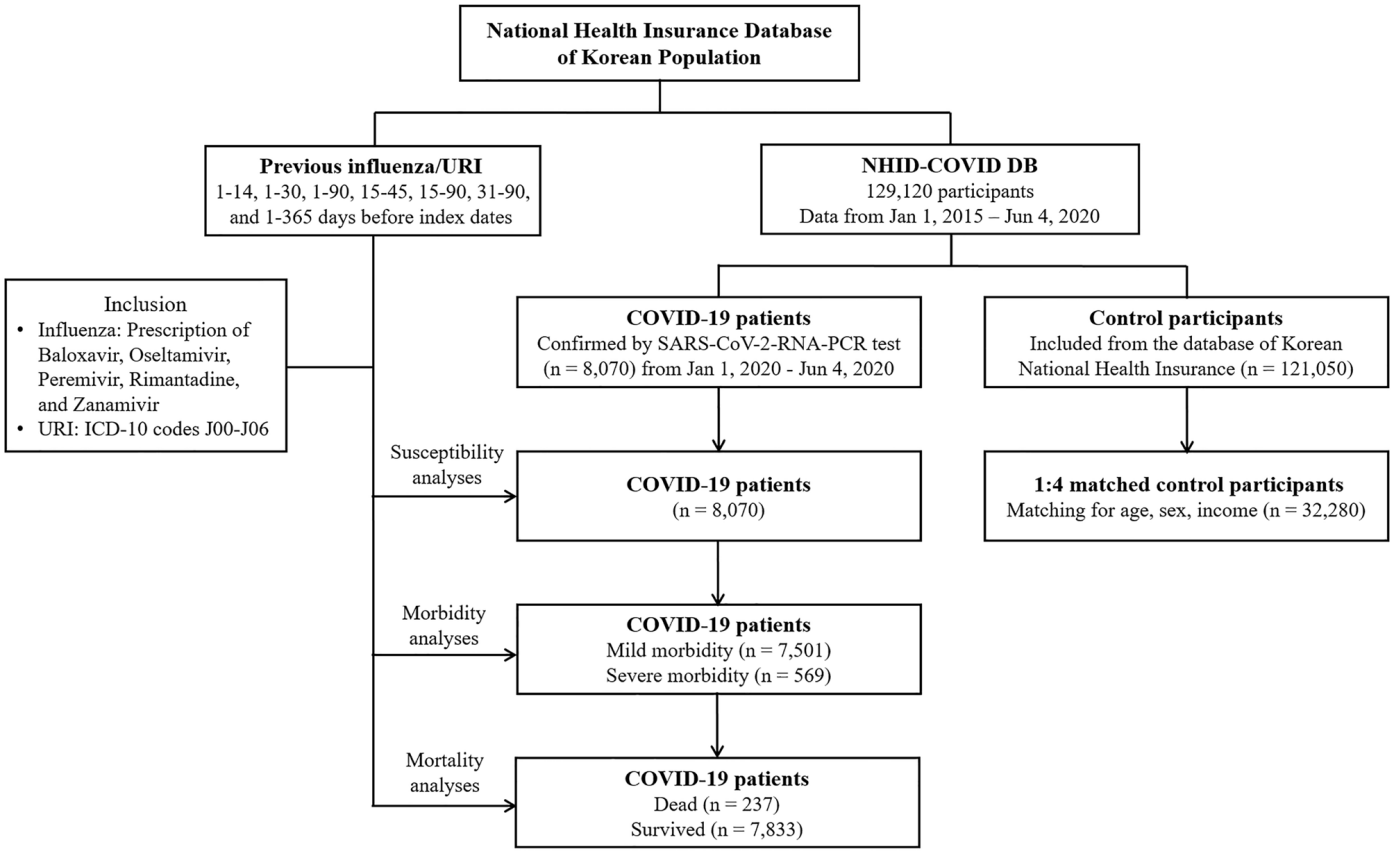
A schematic illustration of the participant selection process that was used in the present study. Of a total of 129,120 participants, 8,070 COVID-19 participants were matched with 32,280 control participants for age, sex, and income.

Confirmed COVID-19 patients (ICD-10: B342, B972, U071, U072) were included from 1 January 2020 through 4 June 2020, and all of them finished treatment or died as of 4 June 2020 (n = 8,070). Non-COVID-19 participants (control) were extracted, with 15-fold more confirmed COVID-19 cases (n = 121,050). We matched COVID-19 patients with control participants at a 1:4 ratio in terms of age, sex, and income. Among the control participants, we excluded participants with a lack of income records (n = 2,136). To avoid selection bias, control participants were selected randomly using clustered sampling. The index date was defined as the date of COVID-19 confirmation. Each index date of control participants was selected randomly from 1 January 2020 to 4 June 2020. As a consequence, the 8,070 COVID-19 participants were matched 1:4 with 32,280 control participants. The data of previous histories of influenza/URI were merged for the COVID-19 and control participants. The COVID-19 patients were divided by morbidity into mild (n = 569) and severe groups (n = 7,501). They were also divided into dead (n = 237) and surviving (n = 7,833) participants (Fig. 1).

### Exposures

Influenza was defined if the participant was prescribed baloxavir, oseltamivir, peremivir, or zanamivir. In Korea, these medications can be prescribed only after confirmation of influenza (type A or B) using rapid chromatographic immunoassay or real-time RT-PCR. We checked whether the participants had influenza in the previous periods of 1–14, 1–30, 1–90, 15–45, 15–90 and 31–90 days before the index dates. In addition, the total number of prescriptions 1–365 days before the index date was counted as the continuous variable.

URI was defined using the diagnosis codes (ICD-10) J00 (acute nasopharyngitis) through J06 (acute upper respiratory infections of multiple and unspecified sites). We checked if the participant had a URI in the previous periods of 1–14, 1–30, 1–90, 15–45, 15–90 and 31–90 days before the index dates. In addition, the total number of diagnoses 1–365 days before the index date was counted as the continuous variable.

### Outcomes

Laboratory confirmation of SARS-CoV-2 infection was defined as the primary outcome.

The secondary outcomes were morbidity and mortality in COVID-19 patients. Morbidity was defined as severe compared to mild morbidity. Severe morbidity was defined as admission to the ICU, invasive ventilation, extracorporeal membrane oxygenation (ECMO) or death. Mild morbidity was defined as other cases.

### Covariates

Age groups were divided into 10-year intervals: 0–9, 10–19, 20–29,…, and 80 + years old (total of nine age groups). Eleven income groups were reclassified into three classes (low income, middle income, and high income). Missing income [n = 127 (0.31%)] was replaced by middle income groups. The Charlson comorbidity index (CCI) has been used widely to measure disease burden, using 17 comorbidities as the continuous variable (0 [no comorbidities] through 29 [multiple comorbidities]) without respiratory diseases. Regarding influenza and URI, asthma (ICD-10 codes: J45 and J46) and chronic obstruction pulmonary disease (COPD, ICD-10 codes: J42 to J44, except J430) were additionally assigned if participants were treated ≥ two times and prescribed related medications, following our previous study^15^. In addition, hypertension (ICD-10 codes: I10 and I15) was additionally assigned if participants were treated ≥ two times, as this was not included in the CCI score.

### Statistical analyses

The general characteristics between the COVID-19 group and control group and between the severe morbidity group and mild morbidity group were compared using the chi-square or Fisher’s exact test for categorical variables and using the independent *t* test for continuous variables.

To estimate the susceptibility of COVID-19 patients compared to that of control participants, odds ratios (ORs) with 95% confidence intervals (CIs) of influenza/URI were calculated using a crude model (simple model), model 1 (adjusted for CCI score, asthma, COPD, and hypertension), and model 2 (adjusted for model 1 plus influenza and URI) with conditional logistic regression. In these analyses, age, sex, and income were stratified. To estimate morbidity/mortality in COVID-19 patients by influenza/URI, unconditional logistic regression was used for the unmatched analyses. Conditional logistic regression was used as this allows for micro adjustment of covariates and to address the unequal sizes in the matched pairs after the exclusion of patients missing demographic information (age, sex, income, and region).

To estimate the effects of the unmeasured confounders, the E-values of influenza and URI for COVID-19 infection and mortality of COVID-19 were calculated^16^. If the E-value was higher than the relations with unmeasured confounders, the association of influenza/URI with infection/mortality of COVID-19 could be valid^16^.

For the subgroup analyses, we divided participants by age (< 50 years old and ≥ 50 years old), sex, income (low, middle, and high), CCI scores (one score, two scores, and ≥ two scores), asthma, COPD, and hypertension history.

For statistical analyses, SAS version 9.4 (SAS Institute Inc., Cary, NC, USA) was used. We performed two-tailed analyses, and significance was defined as P values less than 0.05.

## Results

### COVID-19 susceptibility

**Figure 2:**
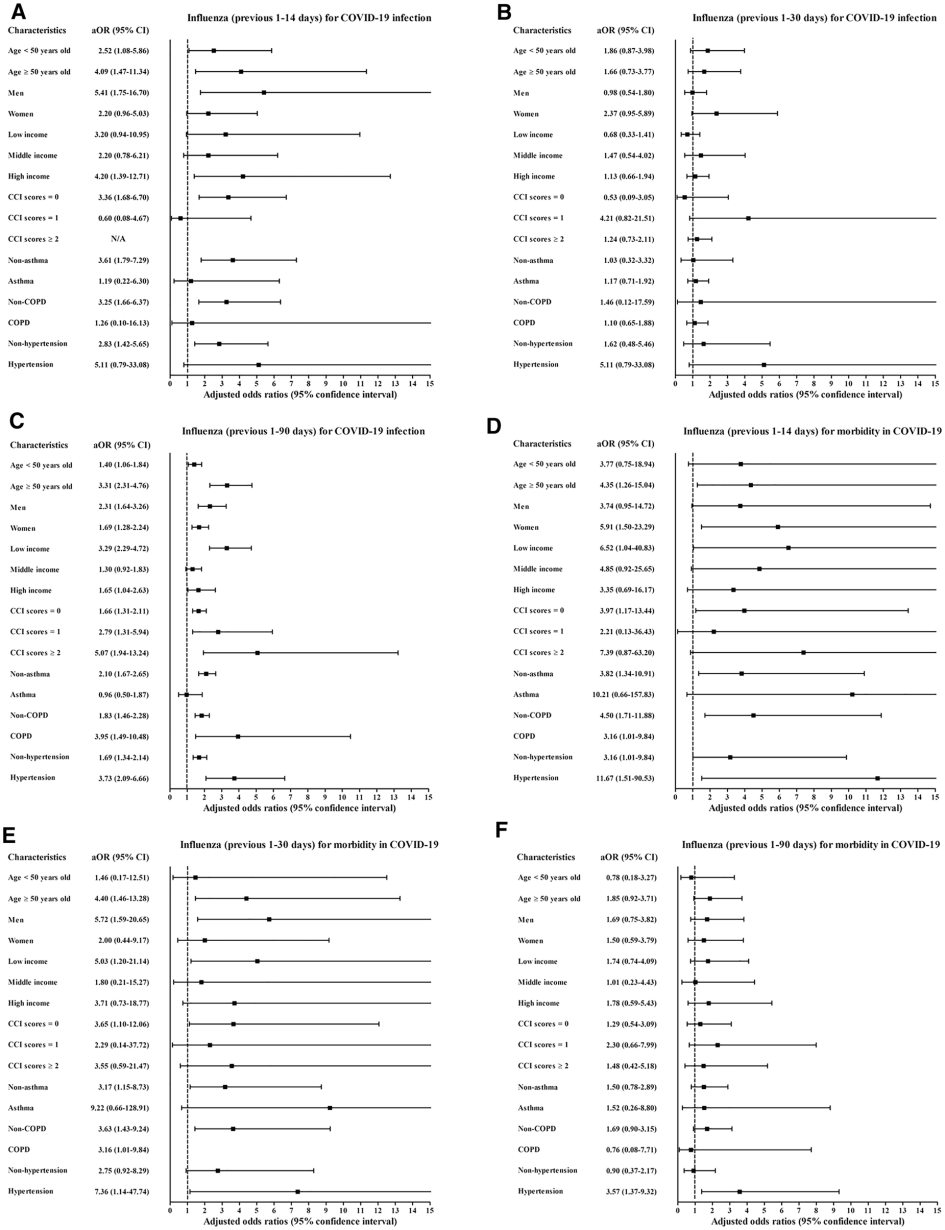

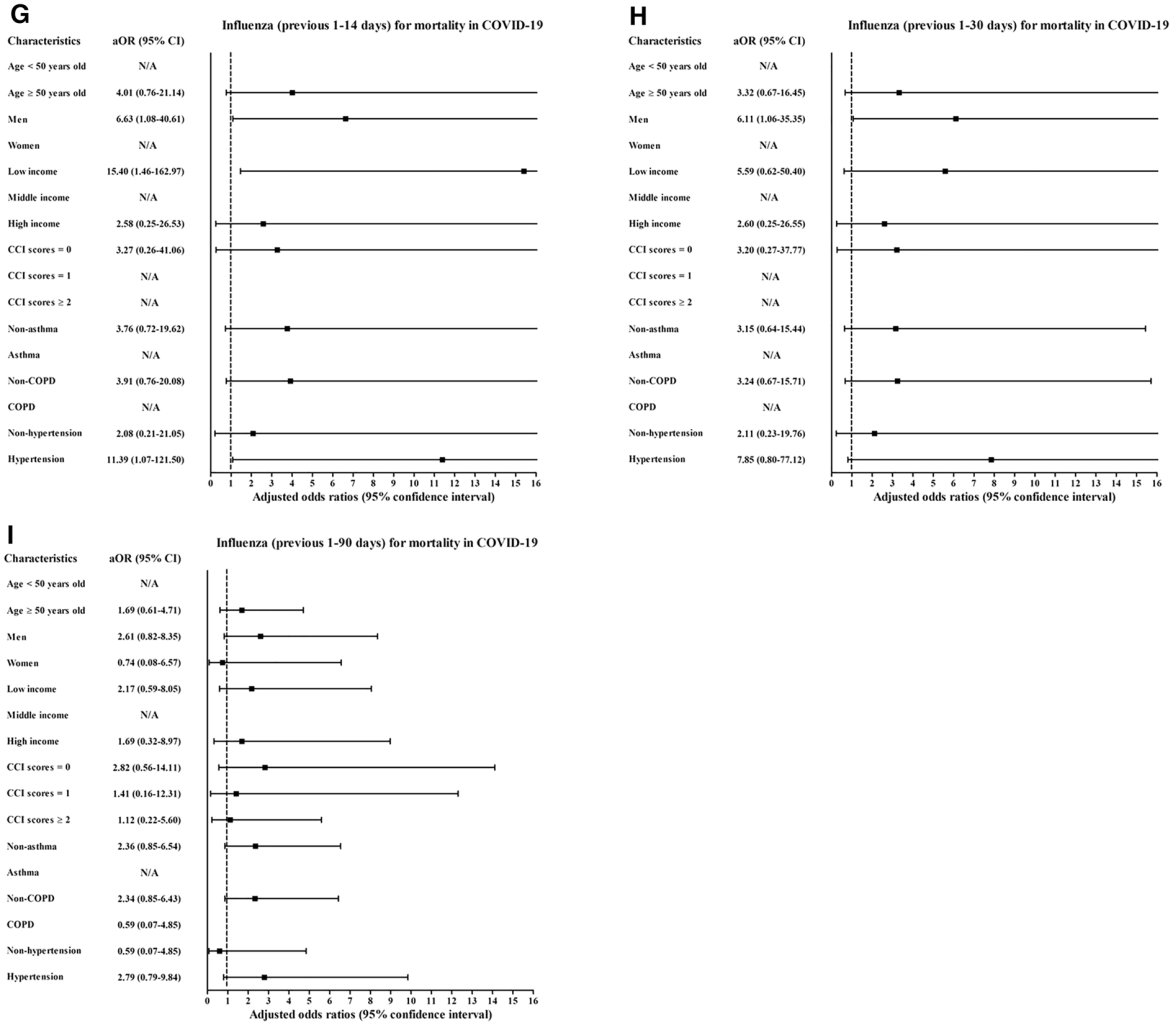
Subgroup analyses of the participants with COVID-19 by previous histories of influenza regarding (**A**) susceptibility for 1–14 days, (**B**) 1–30 days (**C**), and 1–90 days; (**D**) morbidity for 1–14 days, (**E**) 1–30 days, (**F**) and 1–90 days; and (**G**) mortality for 1–14 days, (**H**) 1–30 days, and (**I**) 1–90 days.

**Figure 3:**
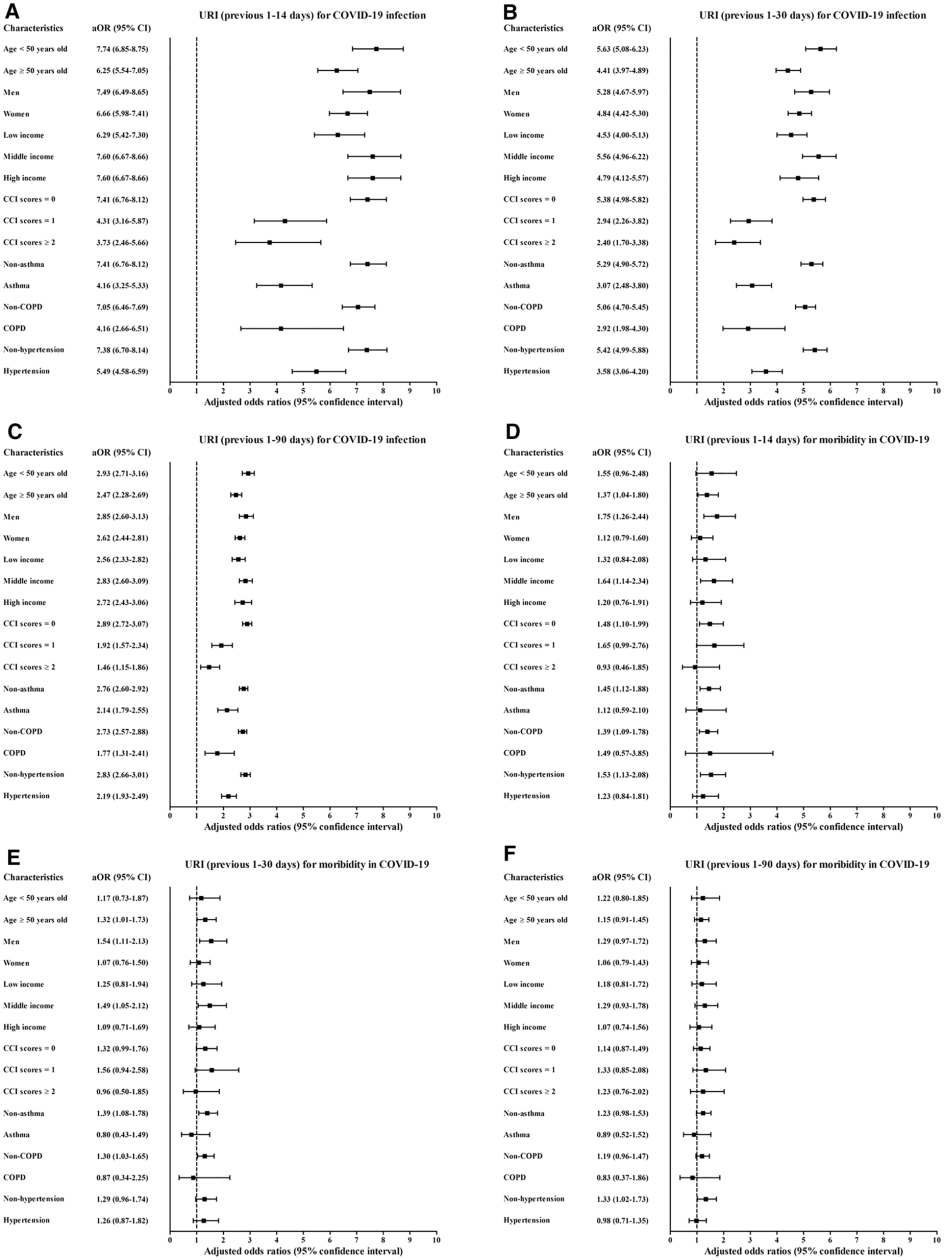

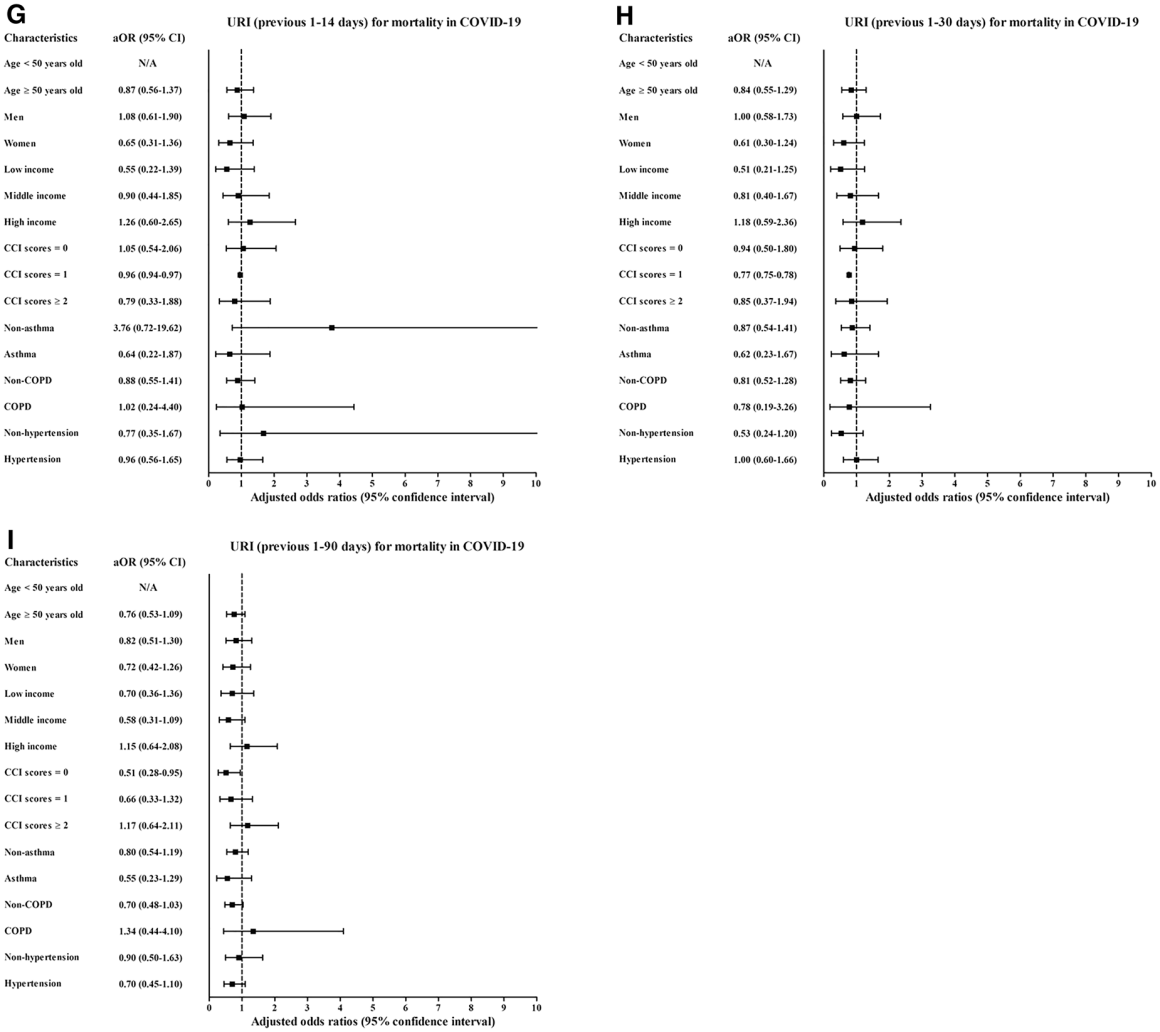
Subgroup analyses of the participants for COVID-19 susceptibility/morbidity/mortality by previous histories of URI regarding (**A**) susceptibility for 1–14 days, (**B**) 1–30 days (**C**), and 1–90 days; (**D**) morbidity for 1–14 days, (**E**) 1–30 days, (**F**) and 1–90 days; and (**G**) mortality for 1–14 days, (**H**) 1–30 days, and (**I**) 1–90 days.

Histories of influenza infections and URI in the previous 1–14 days, 1–30 days, and 1–90 days were more common in the COVID-19 patients than in the control participants (all P < 0.001, Table 1). Previous histories of influenza infection and URI were associated with increased positivity for SARS-CoV-2 infection (Table 2). The histories of influenza infections were related to 3.07-fold (1.61–5.85) and 1.91-fold (1.54–2.37) higher rates of COVID-19 in the previous 1–14 days and 1–90 days, respectively. A history of URI was associated with higher rates of COVID-19 in the previous 1–14 days (6.95, [6.37–7.58]), 1–30 days (4.99 [4.64–5.37]), and 1–90 days (2.70 [2.55–2.86]). The COVID-19 susceptibility associated with previous histories of influenza infection or URI was consistent in all subgroups according to age, sex, income, CCI score, and histories of asthma, COPD, and hypertension (Figs. 2A–C, 3A–C, and Table S1).

**Table 1:** General characteristics of participants.

Characteristics	Total participants			COVID-19 participants		
	COVID-19	Control	P-value	Severe morbidity	Mild morbidity	P-value
Total number (n, %)	8,070 (100.0)	32,280 (100.0)		569 (100.0)	7,501 (100.0)	
Age (years old) (n, %)			1.000			< 0.001*
0–9	81 (1.0)	324 (1.0)		6 (1.1)	75 (1.0)	
10–19	276 (3.4)	1,104 (3.4)		6 (1.1)	270 (3.6)	
20–29	2,057 (25.5)	8,228 (25.5)		31 (5.5)	2,026 (27.0)	
30–39	832 (10.3)	3,328 (10.3)		25 (4.4)	807 (10.8)	
40–49	1,036 (12.8)	4,144 (12.8)		30 (5.3)	1,006 (13.4)	
50–59	1,567 (19.4)	6,268 (19.4)		71 (12.5)	1,496 (19.9)	
60–69	1,199 (14.9)	4,796 (14.9)		116 (20.4)	1,083 (14.4)	
70–79	617 (7.7)	2,468 (7.7)		118 (20.7)	499 (6.7)	
80 +	405 (5.0)	1,620 (5.0)		166 (29.2)	239 (3.2)	
Sex (n, %)			1.000			< 0.001*
Male	3,236 (40.1)	12,944 (40.1)		306 (53.8)	2,930 (39.1)	
Female	4,834 (59.9)	19,336 (59.9)		263 (46.2)	4,571 (60.9)	
Income (n, %)			1.000			< 0.001*
1 (low)	2,836 (35.1)	11,344 (35.1)		185 (32.5)	2,651 (35.3)	
2	3,325 (41.2)	13,300 (41.2)		211 (37.1)	3,114 (41.5)	
3 (high)	1,909 (23.7)	7,636 (23.7)		173 (30.4)	1,736 (23.1)	
CCI score (n, %)			< 0.001*			< 0.001*
0	6,518 (80.8)	29,539 (91.5)		264 (46.4)	6,254 (83.4)	
1	889 (11.0)	1,416 (4.4)		134 (23.6)	755 (10.1)	
≥ 2	663 (8.2)	1,325 (4.1)		171 (30.1)	492 (6.6)	
Asthma (n, %)	704 (8.7)	2,613 (8.1)	0.066	86 (15.1)	618 (8.2)	< 0.001*
COPD (n, %)	264 (3.3)	883 (2.7)	0.001*	55 (9.7)	209 (2.8)	< 0.001*
Hypertension (n, %)	1,657 (20.5)	6,428 (19.9)	0.214	275 (48.3)	1,382 (18.4)	< 0.001*
Previous 1–14 days (n, %)						
Influenza	27 (0.3)	20 (0.1)	< 0.001*	6 (1.1)	21 (0.3)	0.003*
URI	1,442 (17.9)	1,003 (3.1)	< 0.001*	108 (19.0)	1,333 (17.8)	0.506
Previous 1–30 days (n, %)						
Influenza	32 (0.4)	48 (0.2)	< 0.001*	7 (1.2)	25 (0.3)	0.006*
URI	1,741 (21.6)	1,741 (5.4)	< 0.001*	119 (20.9)	1,622 (21.6)	0.691
Previous 1–90 days (n, %)						
Influenza	146 (1.8)	229 (0.7)	< 0.001*	14 (2.5)	132 (1.8)	0.227
URI	2,694 (33.4)	5,130 (15.9)	< 0.001*	177 (31.1)	2,517 (33.6)	0.233

CCI, Charlson comorbidity index; COPD, chronic obstructive pulmonary disease; upper respiratory tract infection, URI; COVID-19, coronavirus disease 2019; SD, standard deviation.

*Chi-square or Fisher's exact test. Significance at P < 0.05.

^†^Independent *t* test. Significance at P < 0.05.

**Table 2:** Crude and adjusted odds ratios of influenza and URI (previous 1–14, 1–30, and 1–90 days) for COVID-19 infection in all participants.

Characteristics	COVID-19 (exposure/total, %)	Control (exposure/total, %)	ORs (95% confidence interval) for COVID-19						E-value (95% CI)
			Crude^†^	P-value	Model 1^†‡^	P-value	Model 2^†^§	P-value	
**Previous 1–14 days**									
Influenza	27/8,070 (0.3%)	20/32,280 (0.1%)	5.62 (3.16–9.98)	< 0.001*	5.37 (3.01–9.60)	< 0.001*	3.07 (1.61–5.85)	0.001*	5.59 (2.60–11.18)
URI	1,441/8,070 (17.9%)	1,003/32,280 (3.1%)	6.82 (6.26–7.43)	< 0.001*	7.00 (6.42–7.62)	< 0.001*	6.95 (6.38–7.58)	< 0.001*	13.38 (12.24–14.64)
**Previous 1–30 days**									
Influenza	32/8,070 (0.4%)	48/32,280 (0.1%)	2.68 (1.71–4.19)	< 0.001*	2.46 (1.56–3.87)	< 0.001*	1.18 (0.72–1.91)	0.510	1.00 (1.00–1.67)
URI	1,741/8,070 (21.6%)	1,741/32,280 (5.4%)	4.87 (4.53–5.23)	< 0.001*	5.00 (4.65–5.38)	< 0.001*	4.99 (4.64–5.37)	< 0.001*	9.45 (8.75–10.21)
**Previous 1–90 days**									
Influenza	146/8,070 (1.8%)	229/32,280 (0.7%)	2.60 (2.10–3.20)	< 0.001*	2.52 (2.04–3.12)	< 0.001*	1.91 (1.54–2.37)	< 0.001*	3.23 (2.45–4.17)
URI	2,694/8,070 (33.4%)	5,130/32,280 (15.9%)	2.68 (2.54–2.84)	< 0.001*	2.73 (2.58–2.89)	< 0.001*	2.70 (2.55–2.86)	< 0.001*	4.84 (4.54–5.17)

*Conditional logistic regression model, Significance at P < 0.05.

^†^Stratified model for age, sex and income.

^‡^Model 1 was adjusted for CCI scores, asthma, COPD, and hypertension.

^§^Model 2 was adjusted for model 1 plus influenza and URI.

To assess possible coinfection, additional analyses were conducted for COVID-19 susceptibility, morbidity, and mortality associated with influenza/URI in the previous 15–45 days, 15–90 days, and 31–90 days (Table S2). The previous histories of influenza and URI for all of these periods demonstrated positive associations with susceptibility to COVID-19 (Table S3). The COVID-19 patients showed an increased number of medical visits for influenza/URI during the previous 1–365 days. These associations of previous influenza/URI with susceptibility to COVID-19 were valid in all subgroups according to age, sex, income, and past medical histories (Table S4).

### COVID-19 morbidity

The severe COVID-19 patients were older, more likely to be male, and in lower income groups than mild COVID-19 patients (all P < 0.001) (Table 1). The rates of high CCI score, asthma, COPD, and hypertension were higher in the severe COVID-19 patients than in the mild COVID-19 patients (all P < 0.001). The severe COVID-19 patients showed a higher rate of history of influenza infections for the previous 1–14 days and previous 1–30 days (P = 0.003 and P = 0.006, respectively).

A history of influenza/URI was associated with increased COVID-19 morbidity (Table 3). The history of influenza infection was related to 3.64-fold (1.55–9.21) and 3.59-fold (1.42–9.05) higher rates of morbidity for the previous 1–14 days and 1–30 days, respectively. URI history was associated with 1.40-fold (1.11–1.78) and 3.59-fold (1.02–1.61) higher rates of morbidity for the previous 1–14 days and 1–30 days, respectively. The association of previous histories of influenza/URI with COVID-19 morbidity was solid in the ≥ 50-year-old subgroups, low- and middle-income subgroups, no past medical history subgroup (CCI score = 0), and no past medical histories of asthma and COPD subgroups (Figs. 2D–F, 3D–F, and Table S5).

**Table 3:** Crude and adjusted odds ratios of influenza and URI (previous 1–14, 1–30, and 1–90 days) for morbidity in COVID-19 participants.

Characteristics	Severe participants	Mild participants	ORs (95% confidence interval) for morbidity						E-value (95% CI)
	(exposure/total, %)	(exposure/total, %)	Crude	P-value	Model 1^†^	P-value	Model 2^†‡^	P-value	
**Previous 1–14 days**									
Influenza	6/569 (1.1%)	21/7,501 (0.3%)	3.77 (1.68–8.80)	< 0.001*	3.89 (1.93–10.01)	< 0.001*	3.64 (1.55–9.21)	0.001*	6.74 (2.47–17.91)
URI	108/569 (19.0%)	1,333/7,501 (17.8%)	1.10 (0.88–1.36)	0.406	1.43 (1.13–1.81)	0.003*	1.40 (1.11–1.78)	0.005*	2.15 (1.46–2.96)
**Previous 1–30 days**									
Influenza	7/569 (1.2%)	25/7,501 (0.3%)	3.73 (1.60–8.65)	0.002*	3.82 (1.53–9.55)	0.004*	3.59 (1.42–9.05)	0.007*	6.64 (2.19–17.59)
URI	119/569 (20.9%)	1,622/7,501 (21.6%)	0.96 (0.78–1.18)	0.692	1.30 (1.03–1.64)	0.025*	1.28 (1.02–1.61)	0.037*	1.88 (1.16–2.60)
**Previous 1–90 days**									
Influenza	14/569 (2.5%)	132/7,501 (1.8%)	1.41 (0.81–2.46)	0.227	1.57 (0.86–2.89)	0.144	1.54 (0.84–2.84)	0.162	1.00 (1.00–5.13)
URI	177/569 (31.1%)	2,517/7,501 (33.6%)	0.89 (0.74–1.08)	0.233	1.17 (0.96–1.44)	0.124	1.17 (0.95–1.43)	0.137	1.00 (1.00–2.21)

*Unconditional logistic regression model, Significance at P < 0.05.

^†^Model 1 was adjusted for age, sex, income, CCI scores, asthma, COPD, and hypertension.

^‡^Model 2 was adjusted for model 1 plus influenza and URI.

Histories of influenza/URI in the previous 15–45 days, 15–90 days, and 31–90 days were not associated with severe COVID-19 (Table S6). The number of medical visits for the previous 1–365 days for influenza/URI was not related to severe COVID-19. The ≥ 50-year-old subgroup and the subgroups with CCI scores ≥ 2 and hypertension showed positive associations of the number of medical visits for influenza during the previous 1–365 days with COVID-19 morbidity (Table S7).

### COVID-19 mortality

Overall, COVID-19 mortality did not show an association with previous histories of influenza/URI for 1–14 days, 1–30 days, and 1–90 days (Table 4). The histories of influenza/URI in previous 15–45 days, 15–90 days, and 31–90 days and the number of medical visits for influenza/URI during the previous 1–365 days were not related to COVID-19 mortality (Table S8).

**Table 4:** Crude and adjusted odds ratios of influenza and URI (previous 1–14, 1–30, and 1–90 days) for mortality in COVID-19 participants.

Characteristics	Dead participants	Survived participants	ORs (95% confidence interval) for mortality						E-value (95% CI)
	(exposure/total, %)	(exposure/total, %)	Crude	P-value	Model 1^†^	P-value	Model 2^†‡^	P-value	
**Previous 1–14 days**									
Influenza	2/237 (0.8%)	25/7,833 (0.3%)	2.56 (0.60–10.83)	0.203	3.50 (0.69–17.82)	0.132	3.66 (0.71–18.81)	0.120	1.00 (1.00–37.11)
URI	27/237 (11.3%)	1,414/7,833 (18.1%)	0.62 (0.41–0.91)	0.015*	0.93 (0.60–1.44)	0.731	0.90 (0.58–1.40)	0.634	1.00 (1.00–2.84)
**Previous 1–30 days**									
Influenza	2/237 (0.8%)	30/7,833 (0.4%)	2.22 (0.53–9.32)	0.278	2.89 (0.60–14.04)	0.188	3.12 (0.64–15.19)	0.158	1.00 (1.00–29.87)
URI	30/237 (12.7%)	1,711/7,833 (21.8%)	0.52 (0.35–0.76)	< 0.001*	0.84 (0.55–1.29)	0.422	0.82 (0.54–1.26)	0.365	1.00 (1.00–3.11)
**Previous 1–90 days**									
Influenza	5/237 (2.1%)	141/7,833 (1.8%)	1.18 (0.48–2.90)	0.725	1.59 (0.58–4.39)	0.372	1.62 (0.59–4.47)	0.354	1.00 (1.00–8.41)
URI	49/237 (20.7%)	2,645/7,833 (33.8%)	0.51 (0.37–0.70)	< 0.001*	0.77 (0.54–1.10)	0.151	0.77 (0.54–1.10)	0.146	1.00 (1.00–3.11)

*Unconditional logistic regression model, Significance at P < 0.05.

^†^Model 1 was adjusted for age, sex, income, CCI scores, asthma, COPD, and hypertension.

^‡^Model 2 was adjusted for model 1 plus influenza and URI.

However, the OR of mortality was increased in some subgroups (Figs. 2G–I, 3G–I, Table S9, and Table S10). The subgroups of men (6.63 [1.08–40.61]), low income (15.40 [1.46–162.97]), and hypertension history (11.39 [1.07–121.50]) showed positive associations between influenza in the previous 1–14 days and COVID-19 (Table S9).

## Discussion

A previous history of influenza/URI was associated with increased SARS-CoV-2 positivity in this study. These positive associations were valid up to the previous periods of 1–90 days before COVID-19 onset. Severe COVID-19 morbidity was also associated with recent histories of influenza/URI. On the other hand, COVID-19 mortality was not related overall, but it was linked in some subgroups. Furthermore, this study evaluated the morbidity/mortality of subsequent COVID-19 infection after influenza infection or URI for the first time, while previous studies reported the increased morbidity/mortality of coinfected patients with SARS-CoV-2 and influenza^7,8,11^.

The COVID-19 susceptibility rate was increased in the patients with previous histories of influenza/URI in the present study. This result was contrary to previous studies that estimated a lower rate of COVID-19 in conditions with other viral pathogens^8,17,18^. These previous studies mainly investigated the effects of the positivity of other pathogens^8,18^ or the positivity of influenza-specific IgM^19^ on the coinfection of SARS-CoV-2. However, a serum antibody test study demonstrated that although approximately 23% of COVID-19 patients had SARS-CoV-2 cross-reactive antibodies during the prepandemic period, these antibodies were not neutralizing antibodies for SARS-CoV-2 and were not related to reducing COVID-19 susceptibility and hospitalization^20^. A cross-sectional study reported different contracting patterns of influenza viruses and SARS-CoV-2^21^. There were no coinfected cases in their study, and SARS-CoV-2 infection was higher in elderly individuals, while influenza virus infection was more prevalent in children less than 5 years old^21^. Furthermore, the antiinfection effects of nonpharmacologic interventions, such as wearing face masks, social distancing, restrictions on movement and health behaviors of infected patients, could mediate the decreased COVID-19 incidence in patients with influenza/URI^22–24^.

The higher susceptibility might be partially attributed to the underlying immune capability or environmental factors that could promote susceptibility to both SARS-CoV-2 and influenza/URI. A compromised immune response may increase susceptibility to respiratory viral infections. Respiratory viruses are able to block interferon activation and signaling^25^. Mucociliary clearance dysfunction by respiratory viruses could contribute to coinfection with other viruses^26^. Lymphopenia or T cell exhaustion induced by influenza virus^25^ may be the cause of higher susceptibility to COVID-19. In addition, low socioeconomic status and poor hygiene could support high susceptibility to respiratory viral infections^27^. Occupation, residence, and social activity factors could also influence the opportunity for contact with viral pathogens^28^.

Severe COVID-19 morbidity was associated with previous histories of influenza/URI in this study. The incompetency of immune responses to viral infections could be linked with COVID-19 severity in patients with previous influenza/URI histories. A meta-analysis demonstrated that increased white blood cell counts, decreased lymphocyte and platelet counts, elevated biomarkers for inflammation, cardiac and muscle injury, liver and kidney dysfunction, and coagulation dysfunction were related to severe COVID-19^29^. These biomarkers for severe COVID-19 were also associated with increased vulnerability to influenza/URI^30^. The incomplete recovery of immune and inflammatory responses due to the recent histories of influenza/URI could impose a more severe clinical presentation for subsequent SARS-CoV-2 infection, as the more remote infection histories of influenza/URI were not related to the more severe morbidity of COVID-19 in this study (Table S3). A retrospective cohort study reported higher levels of inflammatory cytokines, including interleukin-2R (IL-2L), IL-6, IL-8, and tumor necrosis factor-α, in coinfected patients with influenza A virus and SARS-CoV-2 than in patients with SARS-CoV-2 infection only^31^.

Several limitations should be considered when interpreting the current results. Ethnic and regional differences might exist for the association of previous influenza/URI with susceptibility, morbidity, and mortality of COVID-19^32,33^. The diagnosis of URI did not include laboratory results and could not differentiate viral or bacterial etiologies. For influenza infection, patients who were prescribed anti-influenza medications without health insurance coverage could not be included in this study. However, all Koreans are legally registered to the national health insurance system, and the proportion of uninsured prescriptions might be rare. Because of the relatively low number of deaths from COVID-19 in Korea, this aspect could not be concluded by the low statistical power. Finally, although we analyzed six periods to minimize the effects of coinfections, the possible impacts of coinfections could not be totally excluded in this study.

In summary, previous influenza/URI histories were associated with elevated COVID-19 susceptibility and morbidity in Korea. This study provides insight into why controlling influenza/URI could also be important during the COVID-19 pandemic. Caution regarding the potential risk of COVID-19 associated with previous influenza/URI infection might be an important infection prevention and control strategy.

## Supplementary Information

Supplementary Information 1.

Supplementary Information 2.

Supplementary Information 3.

Supplementary Information 4.

Supplementary Information 5.

Supplementary Information 6.

Supplementary Information 7.

Supplementary Information 8.

Supplementary Information 9.

Supplementary Information 10.

## Data Availability

Release of the data by the researcher is not allowed legally. All data are available from the database of Korea Centers for Disease Control and Prevention (kdca.go.kr). The Korea Centers for Disease Control and Prevention provides all of these data for any researcher who promises to follow the research ethics guidelines, with some cost. If you want to access the data of this article, you could download it from the website after promising to follow the research ethics guidelines.

## Abbreviations

URI: Upper respiratory infection
COVID-19: Coronavirus disease 2019
SARS-CoV-2: Severe acute respiratory syndrome coronavirus 2
ICU: Intensive care unit
NHID-COVID: Korea National Health Insurance Database Coronavirus Disease 2019
RT-PCR: Real-time reverse transcriptase–polymerase chain reaction
ECMO: Extracorporeal membrane oxygenation
CCI: Charlson comorbidity index
ORs: Odds ratios
CIs: Confidence intervals
COPD: Chronic obstructive pulmonary disease
