# Supplementary material for: The associations of previous influenza/upper respiratory infection with COVID-19 susceptibility/morbidity/mortality: a nationwide cohort study in South Korea
Source: Sci Rep. 2021 Nov 3;11:21568. doi: 10.1038/s41598-021-00428-x (PMC8566493; doi:10.1038/s41598-021-00428-x)
Supplement: Supplementary file 1 — Supplementary Information 1. [file 41598_2021_428_MOESM1_ESM.docx]

**Table S1** Stratified subgroup analyses of Crude and adjusted odds ratios of influenza and URI (previous 1-14, 1-30, and 1-90 days) for COVID-19 infection in total participants by covariates

| Characteristics | | COVID-19 | Control | ORs (95% confidence interval) for COVID-19 | | | | | |
| --- | --- | --- | --- | --- | --- | --- | --- | --- | --- |
|  |  | (exposure/total, %) | (exposure/total, %) | Crude | P-value | Model 1† | P-value | Model 2‡ | P-value |
| **Age < 50 years old ( n = 21,410)** | | | |  |  |  |  |  |  |
| Previous 1-14 days | | | |  |  |  |  |  |  |
|  | Influenza | 14/4,282 (0·3%) | 13/17,128 (0·1%) | 4·31 (2·20-9·73) | <0·001* | 4·32 (2·03-9·17) | <0·001* | 2·52 (1·08-5·86) | 0·032* |
|  | URI | 758/4,282 (17·7%) | 463/17,128 (2·7%) | 7·79 (6·90-8·80) | <0·001* | 7·79 (6·89-8·79) | <0·001* | 7·74 (6·85-8·75) | <0·001* |
| Previous 1-30 days | | | |  |  |  |  |  |  |
|  | Influenza | 16/4,282 (0·4%) | 33/17,128 (0·2%) | 1·94 (1·07-3·54) | 0·029* | 1·79 (0·97-3·28) | 0·061 | 0·85 (0·45-1·63) | 0·624 |
|  | URI | 938/4,282 (21·9%) | 823/17,128 (4·8%) | 5·62 (5·08-6·22) | <0·001* | 5·62 (5·07-6·22) | <0·001* | 5·63 (5·08-6·23) | <0·001* |
| Previous 1-90 days | | | |  |  |  |  |  |  |
|  | Influenza | 81/4,282 (1·9%) | 167/17,128 (1·0%) | 7·46 (2·97-18·72) | <0·001* | 1·93 (1·47-2·53) | <0·001* | 1·40 (1·06-1·84) | 0·019* |
|  | URI | 1473/4,282 (34·4%) | 2,618/17,128 (15·3%) | 5·98 (5·31-6·74) | <0·001* | 2·95 (2·73-3·19) | <0·001* | 2·93 (2·71-3·16) | <0·001* |
| **Age ≥ 50 years old (n = 18,940)** | | | |  |  |  |  |  |  |
| Previous 1-14 days | | | |  |  |  |  |  |  |
|  | Influenza | 13/3,788 (0·3%) | 7/15,152 (0·0%) | 7·46 (2·97-18·72) | <0·001* | 7·36 (2·91-18·61) | <0·001* | 4·09 (1·47-11·34) | 0·007* |
|  | URI | 684/3,788 (18·1%) | 540/15,152 (3·6%) | 5·98 (5·31-6·74) | <0·001* | 6·30 (5·58-7·11) | <0·001* | 6·25 (5·54-7·05) | <0·001* |
| Previous 1-30 days | | | |  |  |  |  |  |  |
|  | Influenza | 16/3,788 (0·4%) | 15/15,152 (0·1%) | 4·28 (2·11-8·66) | <0·001* | 3·92 (1·91-8·05) | <0·001* | 1·86 (0·87-3·98) | 0·110 |
|  | URI | 803/3,788 (21·2%) | 918/15,152 (6·1%) | 4·19 (3·78-4·65) | <0·001* | 4·43 (3·99-4·92) | <0·001* | 4·41 (3·97-4·89) | <0·001* |
| Previous 1-90 days | | | |  |  |  |  |  |  |
|  | Influenza | 65/3,788 (1·7%) | 62/15,152 (0·4%) | 4·26 (3·00-6·05) | <0·001* | 4·12 (2·89-5·87) | <0·001* | 3·31 (2·31-4·76) | <0·001* |
|  | URI | 1,221/3,788 (32·2%) | 2,512/15,152 (16·6%) | 2·41 (2·22-2·61) | <0·001* | 2·51 (2·31-2·72) | <0·001* | 2·47 (2·28-2·69) | <0·001* |
| **Men (n = 16,180)** | | | |  |  |  |  |  |  |
| Previous 1-14 days | | | |  |  |  |  |  |  |
|  | Influenza | 13/3,236 (0·4%) | 5/12,944 (0·0%) | 11·24 (4·05-31·24) | <0·001* | 11·36 (4·07-31·73) | <0·001* | 5·41 (1·75-16·70) | 0·003* |
|  | URI | 542/3,236 (16·7%) | 342/12,944 (2·6%) | 7·46 (6·47-8·59) | <0·001* | 7·59 (6·58-8·76) | <0·001* | 7·49 (6·49-8·65) | <0·001* |
| Previous 1-14 days | | | |  |  |  |  |  |  |
|  | Influenza | 13/3,236 (0·4%) | 14/12,944 (0·1%) | 3·73 (1·75-7·94) | <0·001* | 3·58 (1·67-7·70) | 0·001* | 1·66 (0·73-3·77) | 0·228 |
|  | URI | 627/3,236 (19·4%) | 577/12,944 (4·5%) | 5·20 (4·60-5·87) | <0·001* | 5·31 (4·70-6·00) | <0·001* | 5·28 (4·67-5·97) | <0·001* |
| Previous 1-90 days | | | |  |  |  |  |  |  |
|  | Influenza | 63/3,236 (1·9%) | 82/12,944 (0·6%) | 3·14 (2·25-4·38) | <0·001* | 3·12 (2·23-4·35) | <0·001* | 2·31 (1·64-3·26) | <0·001* |
|  | URI | 971/3,236 (30·0%) | 1,717/12,944 (13·3%) | 2·84 (2·60-3·12) | <0·001* | 2·90 (2·64-3·18) | <0·001* | 2·85 (2·60-3·13) | <0·001* |
| **Women (n = 24,170)** | | | |  |  |  |  |  |  |
| Previous 1-14 days | | | |  |  |  |  |  |  |
|  | Influenza | 14/4,834 (0·3%) | 15/19,336 (0·1%) | 3·55 (1·61-7·16) | <0·001* | 3·44 (1·64-7·22) | 0·001* | 2·20 (0·96-5·03) | 0·061 |
|  | URI | 900/4,834 (18·6%) | 661/19,336 (3·4%) | 6·48 (5·83-7·21) | <0·001* | 6·68 (6·00-7·44) | <0·001* | 6·66 (5·98-7·41) | <0·001* |
| Previous 1-14 days | | | |  |  |  |  |  |  |
|  | Influenza | 19/4,834 (0·4%) | 34/19,336 (0·2%) | 2·24 (1·28-3·93) | 0·005* | 2·01 (1·13-3·57) | 0·017* | 0·98 (0·54-1·80) | 0·953 |
|  | URI | 1114/4,834 (23·0%) | 1,164/19,336 (6·0%) | 4·69 (4·29-5·13) | <0·001* | 4·84 (4·42-5·30) | <0·001* | 4·84 (4·42-5·30) | <0·001* |
| Previous 1-90 days | | | |  |  |  |  |  |  |
|  | Influenza | 83/4,834 (1·7%) | 147/19,336 (0·8%) | 2·29 (1·75-3·01) | <0·001* | 2·21 (1·68-2·90) | <0·001* | 1·69 (1·28-2·24) | <0·001 |
|  | URI | 1723/4,834 (35·6%) | 3,413/19,336 (17·7%) | 2·59 (2·42-2·78) | <0·001* | 2·64 (2·46-2·83) | <0·001* | 2·62 (2·44-2·81) | <0·001* |
| **Low income (n = 14,180)** | | | |  |  |  |  |  |  |
| Previous 1-14 days | | | |  |  |  |  |  |  |
|  | Influenza | 8/2,836 (0·3%) | 6/11,344 (0·1%) | 4·67 (1·57-13·92) | 0·006* | 4·14 (1·37-12·56) | 0·012* | 3·20 (0·94-10·95) | 0·064* |
|  | URI | 454/2,836 (16·0%) | 346/11,344 (3·1%) | 6·09 (5·25-7·05) | <0·001* | 6·31 (5·44-7·32) | <0·001* | 6·29 (5·42-7·30) | <0·001* |
| Previous 1-30 days | | | |  |  |  |  |  |  |
|  | Influenza | 12/2,836 (0·4%) | 11/11,344 (0·1%) | 4·38 (1·93-9·93) | <0·001* | 3·52 (1·51-8·19) | 0·004* | 2·37 (0·95-5·89) | 0·064 |
|  | URI | 563/2,836 (19·9%) | 616/11,344 (5·4%) | 4·35 (3·84-4·92) | <0·001* | 4·55 (4·02-5·15) | <0·001* | 4·53 (4·00-5·13) | <0·001* |
| Previous 1-90 days | | | |  |  |  |  |  |  |
|  | Influenza | 64/2,836 (2·3%) | 64/11,344 (0·6%) | 4·07 (2·87-5·78) | <0·001* | 3·86 (2·71-5·49) | <0·001* | 3·29 (2·29-4·72) | <0·001* |
|  | URI | 894/2,836 (31·5%) | 1,789/11,344 (15·8%) | 2·49 (2·26-2·73) | <0·001* | 2·60 (2·36-2·86) | <0·001* | 2·56 (2·33-2·82) | <0·001* |
| **Middle income (n = 16,625)** | | | |  |  |  |  |  |  |
| Previous 1-14 days | | | |  |  |  |  |  |  |
|  | Influenza | 11/3,325 (0·3%) | 8/13,300 (0·1%) | 5·52 (2·22-13·72) | <0·001* | 5·10 (2·02-12·84) | 0·001* | 2·20 (0·78-6·21) | 0·135 |
|  | URI | 655/3,325 (19·7%) | 421/13,300 (3·2%) | 7·53 (6·62-8·58) | <0·001* | 7·64 (6·71-8·71) | <0·001* | 7·60 (6·67-8·66) | <0·001* |
| Previous 1-30 days | | | |  |  |  |  |  |  |
|  | Influenza | 12/3,325 (0·4%) | 28/13,300 (0·2%) | 1·72 (0·87-3·38) | 0·118 | 1·60 (0·81-3·19) | 0·178 | 0·68 (0·33-1·41) | 0·302 |
|  | URI | 770/3,325 (23·2%) | 702/13,300 (5·3%) | 5·45 (4·88-6·09) | <0·001* | 5·53 (4·94-6·18) | <0·001* | 5·56 (4·96-6·22) | <0·001* |
| Previous 1-90 days | | | |  |  |  |  |  |  |
|  | Influenza | 51/3,325 (1·5%) | 111/13,300 (0·8%) | 1·86 (1·33-2·60) | <0·001* | 1·85 (1·32-2·59) | <0·001* | 1·30 (0·92-1·83) | 0·141 |
|  | URI | 1160/3,325 (34·9%) | 2,134/13,300 (16·0%) | 2·84 (2·60-3·09) | <0·001* | 2·85 (2·61-3·10) | <0·001* | 2·83 (2·60-3·09) | <0·001* |
| **High income (n = 9,454)** | | | |  |  |  |  |  |  |
| Previous 1-14 days | | | |  |  |  |  |  |  |
|  | Influenza | 8/1,909 (0·4%) | 6/7,636 (0·1%) | 6·01 (2·43-18·48) | <0·001* | 7·10 (2·57-19·65) | <0·001* | 4·20 (1·39-12·71) | 0·011* |
|  | URI | 333/1,909 (17·4%) | 236/7,636 (3·1%) | 6·66 (5·59-7·94) | <0·001* | 6·93 (5·80-8·27) | <0·001* | 6·85 (5·73-8·18) | <0·001* |
| Previous 1-30 days | | | |  |  |  |  |  |  |
|  | Influenza | 8/1,909 (0·4%) | 09/7,636 (0·1%) | 3·57 (1·37-9·26) | 0·009* | 3·76 (1·45-9·80) | 0·007* | 1·47 (0·54-4·02) | 0·457 |
|  | URI | 408/1,909 (21·4%) | 423/7,636 (5·5%) | 4·68 (4·04-5·42) | <0·001* | 4·82 (4·15-5·59) | <0·001* | 4·79 (4·12-5·57) | <0·001* |
| Previous 1-90 days | | | |  |  |  |  |  |  |
|  | Influenza | 31/1,909 (1·6%) | 54/7,636 (0·7%) | 2·34 (1·50-3·66) | <0·001* | 2·32 (1·48-3·63) | <0·001* | 1·65 (1·04-2·63) | 0·033* |
|  | URI | 640/1,909 (33·5%) | 1,207/7,636 (15·8%) | 2·72 (2·42-3·04) | <0·001* | 2·75 (2·45-3·09) | <0·001* | 2·72 (2·43-3·06) | <0·001* |
| **CCI scores = 0 (n = 36,057)** | | | |  |  |  |  |  |  |
| Previous 1-14 days | | | |  |  |  |  |  |  |
|  | Influenza | 22/6,518 (0·3%) | 18/29,539 (0·1%) | 5·55 (2·98-10·36) | <0·001* | 5·45 (2·92-10·17) | <0·001* | 3·36 (1·68-6·70) | <0·001* |
|  | URI | 1227/6,518 (18·8%) | 901/29,539 (3·1%) | 7·37 (6·73-8·07) | <0·001* | 7·45 (6·80-8·16) | <0·001* | 7·41 (6·76-8·12) | <0·001* |
| Previous 1-30 days | | | |  |  |  |  |  |  |
|  | Influenza | 24/6,518 (0·4%) | 42/29,539 (0·1%) | 2·60 (1·57-4·29) | <0·001* | 2·54 (1·54-4·20) | <0·001* | 1·13 (0·66-1·94) | 0·657 |
|  | URI | 1495/6,518 (22·9%) | 1,566/29,539 (5·3%) | 5·32 (4·92-5·74) | <0·001* | 5·39 (4·99-5·82) | <0·001* | 5·38 (4·98-5·82) | <0·001* |
| Previous 1-90 days | | | |  |  |  |  |  |  |
|  | Influenza | 112/6,518 (1·7%) | 212/29,539 (0·7%) | 2·42 (1·92-3·05) | <0·001* | 2·37 (1·88-2·98) | <0·001* | 1·66 (1·31-2·11) | <0·001* |
|  | URI | 2291/6,518 (35·1%) | 4,665/29,539 (15·8%) | 2·89 (2·72-3·07) | <0·001* | 2·92 (2·75-3·10) | <0·001* | 2·89 (2·72-3·07) | <0·001* |
| **CCI scores = 1 (n = 2,305)** | | | |  |  |  |  |  |  |
| Previous 1-14 days | | | |  |  |  |  |  |  |
|  | Influenza | 2/889 (0·2%) | 2/1,416 (0·1%) | 1·59 (0·22-11·34) | 0·641 | 1·61 (0·23-11·47) | 0·636 | 0·60 (0·08-4·67) | 0·627 |
|  | URI | 148/889 (16·6%) | 64/1,416 (4·5%) | 4·22 (3·11-5·73) | <0·001* | 4·27 (3·14-5·81) | <0·001* | 4·31 (3·16-5·87) | <0·001* |
| Previous 1-30 days | | | |  |  |  |  |  |  |
|  | Influenza | 2/889 (0·2%) | 4/1,416 (0·3%) | 0·80 (0·15-4·36) | 0·792 | 0·83 (0·15-4·56) | 0·831 | 0·53 (0·09-3·05) | 0·473 |
|  | URI | 169/889 (19·0%) | 106/1,416 (7·5%) | 2·90 (2·24-3·76) | <0·001* | 2·92 (2·25-3·79) | <0·001* | 2·94 (2·26-3·82) | <0·001* |
| Previous 1-90 days | | | |  |  |  |  |  |  |
|  | Influenza | 19/889 (2·1%) | 11/1,416 (0·8%) | 2·79 (1·32-5·89) | 0·007* | 2·81 (1·33-5·95) | 0·007* | 2·79 (1·31-5·94) | 0·008* |
|  | URI | 262/889 (29·5%) | 257/1,416 (18·1%) | 1·88 (1·55-2·30) | <0·001* | 1·92 (1·57-2·34) | <0·001* | 1·92 (1·57-2·34) | <0·001* |
| **CCI scores ≥ 2 (n = 1,988)** | | | |  |  |  |  |  |  |
| Previous 1-14 days | | | |  |  |  |  |  |  |
|  | Influenza | 3/663 (0·5%) | 0/1,325 (0·0%) | N/A |  | N/A |  | N/A |  |
|  | URI | 67/663 (10·1%) | 38/1,325 (2·9%) | 3·81 (2·53-5·74) | <0·001* | 3·91 (2·59-5·91) | <0·001* | 3·73 (2·46-5·66) | <0·001* |
| Previous 1-30 days | | | |  |  |  |  |  |  |
|  | Influenza | 6/663 (0·9%) | 2/1,325 (0·2%) | 6·03 (1·22-29·94) | 0·028* | 5·88 (1·18-29·43) | 0·031* | 4·21 (0·82-21·51) | 0·084 |
|  | URI | 77/663 (11·6%) | 69/1,325 (5·2%) | 2·39 (1·70-3·36) | <0·001* | 2·47 (1·75-3·47) | <0·001* | 2·40 (1·70-3·38) | <0·001* |
| Previous 1-90 days | | | |  |  |  |  |  |  |
|  | Influenza | 15/663 (2·3%) | 6/1,325 (0·5%) | 5·09 (1·97-13·18) | <0·001* | 5·29 (2·03-13·76) | <0·001* | 5·07 (1·94-13·24) | <0·001* |
|  | URI | 141/663 (21·3%) | 208/1,325 (15·7%) | 1·45 (1·14-1·84) | 0·002* | 1·48 (1·17-1·88) | 0·001* | 1·46 (1·15-1·86) | 0·002* |
| **Non-asthma (n = 37,033)** | | | |  |  |  |  |  |  |
| Previous 1-14 days | | | |  |  |  |  |  |  |
|  | Influenza | 25/7,366 (0·3%) | 16/29,667 (0·1%) | 6·30 (3·36-11·81) | <0·001* | 6·30 (3·36-11·84) | <0·001* | 3·61 (1·79-7·29) | <0·001* |
|  | URI | 1299/7,366 (17·6%) | 848/29,667 (2·9%) | 7·28 (6·65-7·97) | <0·001* | 7·46 (6·81-8·18) | <0·001* | 7·41 (6·76-8·12) | <0·001* |
| Previous 1-30 days | | | |  |  |  |  |  |  |
|  | Influenza | 27/7,366 (0·4%) | 39/29,667 (0·1%) | 2·80 (1·71-4·57) | <0·001* | 2·68 (1·63-4·41) | <0·001* | 1·24 (0·73-2·11) | 0·435 |
|  | URI | 1561/7,366 (21·2%) | 1,468/29,667 (4·9%) | 5·17 (4·78-5·58) | <0·001* | 5·30 (4·91-5·73) | <0·001* | 5·29 (4·90-5·72) | <0·001* |
| Previous 1-90 days | | | |  |  |  |  |  |  |
|  | Influenza | 133/7,366 (1·8%) | 194/29,667 (0·7%) | 2·79 (2·24-3·49) | <0·001* | 2·78 (2·23-3·48) | <0·001* | 2·10 (1·67-2·65) | <0·001* |
|  | URI | 2398/7,366 (32·6%) | 4,452/29,667 (15·0%) | 2·73 (2·58-2·90) | <0·001* | 2·79 (2·63-2·96) | <0·001* | 2·76 (2·60-2·92) | <0·001* |
| **Asthma (n = 3,317)** | | | |  |  |  |  |  |  |
| Previous 1-14 days | | | |  |  |  |  |  |  |
|  | Influenza | 2/704 (0·3%) | 4/2,613 (0·2%) | 1·75 (0·63-11·26) | 0·279 | 1·99 (0·41-9·72) | 0·398 | 1·19 (0·22-6·30) | 0·841 |
|  | URI | 143/704 (20·3%) | 155/2,613 (5·9%) | 4·04 (3·16-5·17) | <0·001* | 4·17 (3·25-5·34) | <0·001* | 4·16 (3·25-5·33) | <0·001* |
| Previous 1-30 days | | | |  |  |  |  |  |  |
|  | Influenza | 5/704 (0·7%) | 9/2,613 (0·3%) | 2·07 (0·69-6·20) | 0·194 | 1·73 (0·55-5·40) | 0·345 | 1·03 (0·32-3·32) | 0·961 |
|  | URI | 180/704 (25·6%) | 273/2,613 (10·4%) | 2·94 (2·39-3·64) | <0·001* | 3·07 (2·48-3·80) | <0·001* | 3·07 (2·48-3·80) | <0·001* |
| Previous 1-90 days | | | |  |  |  |  |  |  |
|  | Influenza | 13/704 (1·8%) | 35/2,613 (1·3%) | 1·39 (0·73-2·63) | 0·319 | 1·29 (0·67-2·48) | 0·444 | 0·96 (0·50-1·87) | 0·913 |
|  | URI | 296/704 (42·0%) | 678/2,613 (25·9%) | 2·07 (1·74-2·46) | <0·001* | 2·14 (1·79-2·55) | <0·001* | 2·14 (1·79-2·55) | <0·001* |
| **Non-COPD (n = 39,203)** | | | |  |  |  |  |  |  |
| Previous 1-14 days | | | |  |  |  |  |  |  |
|  | Influenza | 26/7,806 (0·3%) | 18/31,397 (0·1%) | 5·81 (3·33-10·98) | <0·001* | 5·78 (3·16-10·56) | <0·001* | 3·25 (1·66-6·37) | <0·001* |
|  | URI | 1397/7,806 (17·9%) | 960/31,397 (3·1%) | 6·91 (6·34-7·54) | <0·001* | 7·10 (6·50-7·74) | <0·001* | 7·05 (6·46-7·69) | <0·001* |
| Previous 1-30 days | | | |  |  |  |  |  |  |
|  | Influenza | 31/7,806 (0·4%) | 46/31,397 (0·1%) | 2·72 (1·72-4·29) | <0·001* | 2·52 (1·58-4·00) | <0·001* | 1·17 (0·71-1·92) | 0·540 |
|  | URI | 1687/7,806 (21·6%) | 1,666/31,397 (5·3%) | 4·92 (4·57-5·29) | <0·001* | 5·07 (4·71-5·45) | <0·001* | 5·06 (4·70-5·45) | <0·001* |
| Previous 1-90 days | | | |  |  |  |  |  |  |
|  | Influenza | 137/7,806 (1·8%) | 221/31,397 (0·7%) | 2·52 (2·03-3·12) | <0·001* | 2·48 (2·00-3·08) | <0·001* | 1·83 (1·46-2·28) | <0·001* |
|  | URI | 2605/7,806 (33·4%) | 4,926/31,397 (15·7%) | 2·69 (2·55-2·85) | <0·001* | 2·76 (2·61-2·92) | <0·001* | 2·73 (2·57-2·88) | <0·001* |
| **COPD (n = 1,147)** | | | |  |  |  |  |  |  |
| Previous 1-14 days | | | |  |  |  |  |  |  |
|  | Influenza | 1/264 (0·4%) | 2/883 (0·2%) | 1·68 (0·15-18·55) | 0·674 | 1·86 (0·17-20·69) | 0·613 | 1·26 (0·10-16·13) | 0·857 |
|  | URI | 45/264 (17·0%) | 43/883 (4·9%) | 4·01 (2·58-6·26) | <0·001* | 4·17 (2·66-6·52) | <0·001* | 4·16 (2·66-6·51) | <0·001* |
| Previous 1-30 days | | | |  |  |  |  |  |  |
|  | Influenza | 1/264 (0·4%) | 2/883 (0·2%) | 1·68 (0·15-18·55) | 0·674 | 1·86 (0·17-20·69) | 0·613 | 1·46 (0·12-17·59) | 0·764 |
|  | URI | 54/264 (20·5%) | 75/883 (8·5%) | 2·77 (1·89-4·06) | <0·001* | 2·93 (1·99-4·30) | <0·001* | 2·92 (1·98-4·30) | <0·001* |
| Previous 1-90 days | | | |  |  |  |  |  |  |
|  | Influenza | 9/264 (3·4%) | 8/883 (0·9%) | 3·86 (1·47-10·11) | 0·006* | 4·06 (1·54-10·70) | 0·005* | 3·95 (1·49-10·48) | 0·006* |
|  | URI | 89/264 (33·7%) | 204/883 (23·1%) | 1·69 (1·26-2·28) | <0·001* | 1·78 (1·31-2·42) | <0·001* | 1·77 (1·31-2·41) | <0·001* |
| **Non-hypertension (n = 32,265)** | | | |  |  |  |  |  |  |
| Previous 1-14 days | | | |  |  |  |  |  |  |
|  | Influenza | 23/6,413 (0·4%) | 18/25,852 (0·1%) | 5·14 (2·92-9·94) | <0·001* | 5·06 (2·73-9·38) | <0·001* | 2·83 (1·42-5·65) | 0·003* |
|  | URI | 1155/6,413 (18·0%) | 751/25,852 (2·9%) | 7·34 (6·67-8·09) | <0·001* | 7·43 (6·75-8·19) | <0·001* | 7·38 (6·70-8·14) | <0·001* |
| Previous 1-30 days | | | |  |  |  |  |  |  |
|  | Influenza | 27/6,413 (0·4%) | 41/25,852 (0·2%) | 2·66 (1·64-4·33) | <0·001* | 2·47 (1·51-4·04) | <0·001* | 1·10 (0·65-1·88) | 0·713 |
|  | URI | 1416/6,413 (22·1%) | 1,302/25,852 (5·0%) | 5·34 (4·93-5·80) | <0·001* | 5·43 (5·00-5·89) | <0·001* | 5·42 (4·99-5·88) | <0·001* |
| Previous 1-90 days | | | |  |  |  |  |  |  |
|  | Influenza | 121/6,413 (1·9%) | 205/25,852 (0·8%) | 2·41 (1·92-3·02) | <0·001* | 2·37 (1·89-2·97) | <0·001* | 1·69 (1·34-2·14) | <0·001* |
|  | URI | 2183/6,413 (34·0%) | 3,995/25,852 (15·5%) | 2·82 (2·66-3·00) | <0·001* | 2·86 (2·69-3·05) | <0·001* | 2·83 (2·66-3·01) | <0·001* |
| **Hypertension (n = 8,085)** | | | |  |  |  |  |  |  |
| Previous 1-14 days | | | |  |  |  |  |  |  |
|  | Influenza | 4/1,657 (0·2%) | 2/6,428 (0·0%) | 7·74 (1·42-42·22) | 0·018* | 8·65 (1·58-47·43) | 0·013* | 5·11 (0·79-33·08) | 0·087 |
|  | URI | 287/1,657 (17·3%) | 252/6,428 (3·9%) | 5·13 (4·29-6·14) | <0·001* | 5·53 (4·61-6·63) | <0·001* | 5·49 (4·58-6·59) | <0·001* |
| Previous 1-30 days | | | |  |  |  |  |  |  |
|  | Influenza | 5/1,657 (0·3%) | 7/6,428 (0·1%) | 2·78 (0·88-8·78) | 0·081 | 2·70 (0·85-8·62) | 0·093 | 1·62 (0·48-5·46) | 0·436 |
|  | URI | 325/1,657 (19·6%) | 439/6,428 (6·8%) | 3·33 (2·85-3·89) | <0·001* | 3·59 (3·07-4·21) | <0·001* | 3·58 (3·06-4·20) | <0·001* |
| Previous 1-90 days | | | |  |  |  |  |  |  |
|  | Influenza | 25/1,657 (1·5%) | 24/6,428 (0·4%) | 4·09 (2·33-7·18) | <0·001* | 4·04 (2·29-7·15) | <0·001* | 3·73 (2·09-6·66) | <0·001* |
|  | URI | 511/1,657 (30·8%) | 1135/6,428 (17·7%) | 2·08 (1·84-2·35) | <0·001* | 2·21 (1·95-2·50) | <0·001* | 2·19 (1·93-2·49) | <0·001* |

Abbreviations: COPD, Chronic obstructive pulmonary disease; Upper respiratory tract infection, URI; COVID-19, Coronavirus Disease 2019; N/A, Not applicable; SD, Standard deviation

* Conditional or unconditional logistic regression model, Significance at P < 0·05

† Model 1 was adjusted for age, sex, income, and CCI scores, asthma, COPD, and hypertension

‡ Model 2 was adjusted for model 1 plus influenza and URI
