# Supplementary material for: The associations of previous influenza/upper respiratory infection with COVID-19 susceptibility/morbidity/mortality: a nationwide cohort study in South Korea
Source: Sci Rep. 2021 Nov 3;11:21568. doi: 10.1038/s41598-021-00428-x (PMC8566493; doi:10.1038/s41598-021-00428-x)
Supplement: Supplementary file 2 — Supplementary Information 2. [file 41598_2021_428_MOESM2_ESM.docx]

**Table S2** General Characteristics of Participants

| Characteristics | | Total participants | | | COVID-19 participants | | |
| --- | --- | --- | --- | --- | --- | --- | --- |
|  |  | COVID-19 | Control | P-value | Severe morbidity | Mild morbidity | P-value |
| Total number (n, %) | | 8,070 (100·0) | 32,280 (100·0) |  | 569 (100·0) | 7,501 (100·0) |  |
| Previous 15-45 days (n, %) | | | |  |  |  |  |
|  | Influenza | 35 (0·4) | 62 (0·2) | <0·001* | 2 (0·4) | 33 (0·4) | 1·000 |
|  | URI | 916 (11·4) | 1,957 (6·1) | <0·001* | 51 (9·0) | 865 (11·5) | 0·063 |
| Previous 15-90 days (n, %) | | | |  |  |  |  |
|  | Influenza | 120 (1·5) | 211 (0·7) | <0·001* | 7 (1·2) | 113 (1·5) | 0·600 |
|  | URI | 1,805 (22·4) | 4,661 (14·4) | <0·001* | 113 (19·9) | 1,692 (22·6) | 0·137 |
| Previous 31-90 days (n, %) | | | |  |  |  |  |
|  | Influenza | 112 (1·4) | 181 (0·6) | <0·001* | 6 (1·1) | 106 (1·4) | 0·481 |
|  | URI | 1,472 (18·2) | 4,076 (12·6) | <0·001* | 94 (16·5) | 1,378 (18·4) | 0·270 |
| The number of medical visit previous [1-365](tel:1365) days (days, mean, SD) | | | | |  |  |  |
|  | Influenza | 0·028 (0·16) | 0·018 (0·15) | <0·001† | 0·040 (0·27) | 0·027 (0·18) | 0·104 |
|  | URI | 1·547 (3·12) | 1·205 (3·12) | <0·001† | 1·605 (4·19) | 1·543 (3·02) | 0·650 |

* Chi-square or Fisher's exact test· Significance at P < 0·05

† Independent *t* test· Significance at P < 0·05
