# Supplementary material for: The associations of previous influenza/upper respiratory infection with COVID-19 susceptibility/morbidity/mortality: a nationwide cohort study in South Korea
Source: Sci Rep. 2021 Nov 3;11:21568. doi: 10.1038/s41598-021-00428-x (PMC8566493; doi:10.1038/s41598-021-00428-x)
Supplement: Supplementary file 3 — Supplementary Information 3. [file 41598_2021_428_MOESM3_ESM.docx]

**Table S3** Crude and adjusted odds ratios of influenza and URI (previous 15-45, 15-90, 31-90, and 1-365 days) for COVID-19 infection in total participants

| Characteristics | | COVID-19 | Control | ORs (95% confidence interval) for COVID-19 | | | | | |
| --- | --- | --- | --- | --- | --- | --- | --- | --- | --- |
|  |  | (exposure/total, %) | (exposure/total, %) | Crude† | P-value | Model 1†‡ | P-value | Model 2†§ | P-value |
| **Previous 15-45 days** | | | |  |  |  |  |  |  |
|  | Influenza | 35/8,070 (0·4%) | 62/32,280 (0·2%) | 2·27 (1·50-3·43) | <0·001* | 2·16 (1·42-3·29) | <0·001* | 1·80 (1·18-2·75) | 0·007* |
|  | URI | 916/8,070 (11·4%) | 1,957/32,280 (6·1%) | 1·99 (1·83-2·16) | <0·001* | 2·01 (1·85-2·19) | <0·001* | 2·00 (1·84-2·18) | <0·001* |
| **Previous 15-90 days** | | | |  |  |  |  |  |  |
|  | Influenza | 120/8,070 (1·5%) | 211/32,280 (0·7%) | 2·31 (1·84-2·89) | <0·001* | 2·25 (1·79-2·83) | <0·001* | 1·97 (1·56-2·47) | <0·001* |
|  | URI | 1,805/8,070 (22·4%) | 4,661/32,280 (14·4%) | 1·72 (1·62-1·83) | <0·001* | 1·74 (1·63-1·85) | <0·001* | 1·72 (1·61-1·82) | <0·001* |
| **Previous 31-90 days** | | | |  |  |  |  |  |  |
|  | Influenza | 112/8,070 (1·4%) | 181/32,280 (0·6%) | 2·51 (1·98-3·18) | <0·001* | 2·47 (1·95-3·14) | <0·001* | 2·22 (1·75-2·83) | <0·001* |
|  | URI | 1,472/8,070 (18·2%) | 4,076/32,280 (12·6%) | 1·55 (1·45-1·66) | <0·001* | 1·56 (1·46-1·67) | <0·001* | 1·54 (1·44-1·65) | <0·001* |
| **The number of medical visit previous 1-365 days (Days, mean, SD)** | | | |  |  |  |  |  |  |
|  | Influenza | 0·028 (0·19) | 0·156 (0·19) | 1·37 (1·19-1·56) | <0·001* | 1·33 (1·16-1·53) | <0·001* | 1·30 (1·13-1·49) | <0·001* |
|  | URI | 1·547 (3·12) | 1·206 (2·75) | 1·04 (1·03-1·05) | <0·001* | 1·04 (1·03-1·05) | <0·001* | 1·04 (1·03-1·05) | <0·001* |

* Conditional logistic regression model, Significance at P < 0·05

† Stratified model for age, sex and income·

‡ Model 1 was adjusted for CCI scores, asthma, COPD, and hypertension

§ Model 2 was adjusted for model 1 plus influenza and URI
