# Supplementary material for: The associations of previous influenza/upper respiratory infection with COVID-19 susceptibility/morbidity/mortality: a nationwide cohort study in South Korea
Source: Sci Rep. 2021 Nov 3;11:21568. doi: 10.1038/s41598-021-00428-x (PMC8566493; doi:10.1038/s41598-021-00428-x)
Supplement: Supplementary file 4 — Supplementary Information 4. [file 41598_2021_428_MOESM4_ESM.docx]

**Table S4** Stratified subgroup analyses of Crude and adjusted odds ratios of influenza and URI (previous 15-45, 15-90, 31-90, and 1-365 days) for COVID-19 infection in total participants by covariates

| Characteristics | | COVID-19 | Control | ORs (95% confidence interval) for COVID-19 | | | | | |
| --- | --- | --- | --- | --- | --- | --- | --- | --- | --- |
|  |  | (exposure/total, %) | (exposure/total, %) | Crude | P-value | Model 1† | P-value | Model 2‡ | P-value |
| **Age < 50 years old ( n = 21,410)** | | | |  |  |  |  |  |  |
| Previous 15-45 days | | | |  |  |  |  |  |  |
|  | Influenza | 20/4,282 (0·5%) | 45/17,128 (0·3%) | 1·78 (1·05-3·02) | 0·032* | 1·74 (1·02-2·95) | 0·042* | 1·32 (0·77-2·26) | 0·320 |
|  | URI | 503/4,282 (11·7%) | 924/17,128 (5·4%) | 2·35 (2·09-2·63) | <0·001* | 2·34 (2·09-2·63) | <0·001* | 2·33 (2·08-2·62) | <0·001* |
| Previous 15-90 days | | | |  |  |  |  |  |  |
|  | Influenza | 67/4,282 (1·6%) | 156/17,128 (0·9%) | 1·74 (1·30-2·32) | <0·001* | 1·72 (1·29-2·30) | <0·001* | 1·43 (1·07-1·92) | 0·017* |
|  | URI | 1,013/4,282 (23·7%) | 2,385/17,128 (13·9%) | 1·94 (1·78-2·10) | <0·001* | 1·93 (1·77-2·10) | <0·001* | 1·91 (1·76-2·08) | <0·001* |
| Previous 31-90 days | | | |  |  |  |  |  |  |
|  | Influenza | 63/4,282 (1·5%) | 134/17,128 (0·8%) | 1·90 (1·40-2·57) | <0·001* | 1·89 (1·40-2·56) | <0·001* | 1·62 (1·19-2·19) | 0·002* |
|  | URI | 829/4,282 (19·4%) | 2,073/17,128 (12·1%) | 1·76 (1·61-1·92) | <0·001* | 1·75 (1·60-1·92) | <0·001* | 1·73 (1·58-1·90) | <0·001* |
| The number of medical visit previous 1-365 days (Days, mean, SD) | | | | | |  |  |  |  |
|  | Influenza | 0·034 (0·21) | 0·027 (0·18) | 1·22 (1·03-1·44) | 0·020* | 1·20 (1·01-1·42) | 0·036* | 1·16 (0·98-1·37) | 0·096 |
|  | URI | 1·603 (2·90) | 1·170 (2·58) | 1·06 (1·05-1·08) | <0·001* | 1·06 (1·05-1·07) | <0·001* | 1·06 (1·05-1·07) | <0·001* |
| **Age ≥ 50 years old (n = 18,940)** | | | |  |  |  |  |  |  |
| Previous 15-45 days | | | |  |  |  |  |  |  |
|  | Influenza | 15/3,788 (0·4%) | 17/15,152 (0·1%) | 3·54 (1·77-7·10) | <0·001* | 3·30 (1·62-6·70) | 0·001* | 3·03 (1·49-6·16) | 0·002* |
|  | URI | 413/3,788 (10·9%) | 1,033/15,152 (6·8%) | 1·68 (1·49-1·89) | <0·001* | 1·71 (1·51-1·93) | <0·001* | 1·70 (1·51-1·92) | <0·001* |
| Previous 15-90 days | | | |  |  |  |  |  |  |
|  | Influenza | 53/3,788 (1·4%) | 55/15,152 (0·4%) | 3·91 (2·67-5·71) | <0·001* | 3·77 (2·56-5·53) | <0·001* | 1·43 (1·07-1·92) | 0·017* |
|  | URI | 792/3,788 (20·9%) | 2,276/15,152 (15·0%) | 1·50 (1·37-1·64) | <0·001* | 1·53 (1·40-1·68) | <0·001* | 1·91 (1·76-2·08) | <0·001* |
| Previous 31-90 days | | | |  |  |  |  |  |  |
|  | Influenza | 49/3,788 (1·3%) | 47/15,152 (0·3%) | 4·22 (2·83-6·31) | <0·001* | 4·15 (2·77-6·23) | <0·001* | 3·93 (2·62-5·91) | <0·001* |
|  | URI | 643/3,788 (17·0%) | 2,003/15,152 (13·2%) | 1·34 (1·22-1·48) | <0·001* | 1·37 (1·24-1·51) | <0·001* | 1·35 (1·22-1·49) | <0·001* |
| The number of medical visit previous 1-365 days (Days, mean, SD) | | | | | |  |  |  |  |
|  | Influenza | 0·021 (0·17) | 0·010 (0·12) | 1·71 (1·36-2·16) | <0·001* | 1·64 (1·30-2·07) | <0·001* | 1·61 (1·28-2·03) | <0·001* |
|  | URI | 1·484 (3·36) | 1·246 (2·93) | 1·02 (1·01-1·03) | <0·001* | 1·03 (1·02-1·04) | <0·001* | 1·03 (1·01-1·04) | <0·001* |
| **Men (n = 16,180)** | | | |  |  |  |  |  |  |
| Previous 15-45 days | | | |  |  |  |  |  |  |
|  | Influenza | 7/3,236 (0·2%) | 20/12,944 (0·2%) | 1·40 (0·59-3·32) | 0·443 | 1·37 (0·58-3·26) | 0·479 | 1·11 (0·47-2·67) | 0·809 |
|  | URI | 307/3,236 (9·5%) | 643/12,944 (5·0%) | 2·01 (1·75-2·32) | <0·001* | 2·01 (1·74-2·33) | <0·001* | 2·01 (1·74-2·32) | <0·001* |
| Previous 15-90 days | | | |  |  |  |  |  |  |
|  | Influenza | 50/3,236 (1·5%) | 78/12,944 (0·6%) | 2·60 (1·82-3·73) | <0·001* | 2·58 (1·80-3·70) | <0·001* | 2·23 (1·55-3·21) | <0·001* |
|  | URI | 622/3,236 (19·2%) | 1,547/12,944 (12·0%) | 1·77 (1·59-1·96) | <0·001* | 1·79 (1·61-1·98) | <0·001* | 1·76 (1·59-1·95) | <0·001* |
| Previous 31-90 days | | | |  |  |  |  |  |  |
|  | Influenza | 48/3,236 (1·5%) | 68/12,944 (0·5%) | 2·87 (1·98-4·16) | <0·001* | 2·86 (1·97-4·16) | <0·001* | 2·57 (1·76-3·74) | <0·001* |
|  | URI | 512/3,236 (15·8%) | 1,358/12,944 (10·5%) | 1·61 (1·45-1·80) | <0·001* | 1·63 (1·46-1·82) | <0·001* | 1·60 (1·43-1·79) | <0·001* |
| The number of medical visit previous 1-365 days (Days, mean, SD) | | | | | |  |  |  |  |
|  | Influenza | 0·029 (0·20) | 0·018 (0·15) | 1·49 (1·20-1·85) | <0·001 | 1·47 (1·18-1·83) | 0·001* | 1·42 (1·14-1·77) | 0·002* |
|  | URI | 1·307 (2·73) | 1·011 (2·75) | 1·04 (1·02-1·05) | <0·001* | 1·04 (1·02-1·05) | <0·001* | 1·04 (1·02-1·05) | <0·001* |
| **Women (n = 24,170)** | | | |  |  |  |  |  |  |
| Previous 15-45 days | | | |  |  |  |  |  |  |
|  | Influenza | 28/4,834 (0·6%) | 42/19,336 (0·2%) | 2·68 (1·66-4·33) | <0·001* | 2·55 (1·57-4·14) | <0·001 | 2·14 (1·31-3·49) | 0·002* |
|  | URI | 609/4,834 (12·6%) | 1,314/19,336 (6·8%) | 1·98 (1·79-2·19) | <0·001* | 2·01 (1·81-2·23) | <0·001* | 1·99 (1·80-2·21) | <0·001* |
| Previous 15-90 days | | | |  |  |  |  |  |  |
|  | Influenza | 70/4,834 (1·4%) | 133/19,336 (0·7%) | 2·13 (1·59-2·86) | <0·001* | 2·07 (1·54-2·78) | <0·001* | 1·82 (1·35-2·45) | <0·001* |
|  | URI | 1183/4,834 (24·5%) | 3,114/19,336 (16·1%) | 1·69 (1·57-1·83) | <0·001* | 1·71 (1·58-1·84) | <0·001* | 1·69 (1·57-1·83) | <0·001* |
| Previous 31-90 days | | | |  |  |  |  |  |  |
|  | Influenza | 64/4,834 (1·3%) | 113/19,336 (0·6%) | 2·29 (1·68-3·12) | <0·001* | 2·25 (1·65-3·07) | <0·001* | 2·03 (1·48-2·77) | <0·001* |
|  | URI | 960/4,834 (19·9%) | 2,718/19,336 (14·1%) | 1·52 (1·40-1·65) | <0·001* | 1·52 (1·40-1·66) | <0·001* | 1·51 (1·39-1·64) | <0·001* |
| The number of medical visit previous 1-365 days (Days, mean, SD) | | | | | |  |  |  |  |
|  | Influenza | 0·027 (0·18) | 0·019 (0·16) | 1·30 (1·09-1·54) | 0·003* | 1·26 (1·06-1·50) | 0·009* | 1·23 (1·04-1·47) | 0·017* |
|  | URI | 1·709 (3·35) | 1·336 (2·75) | 1·04 (1·03-1·05) | <0·001* | 1·04 (1·03-1·05) | <0·001* | 1·04 (1·03-1·05) | <0·001* |
| **Low income (n = 14,180)** | | | |  |  |  |  |  |  |
| Previous 15-45 days | | | |  |  |  |  |  |  |
|  | Influenza | 13/2,836 (0·5%) | 17/11,344 (0·1%) | 3·07 (1·49-6·34) | 0·002* | 2·70 (1·28-5·67) | 0·009* | 2·31 (1·09-4·89) | 0·028* |
|  | URI | 330/2,836 (11·6%) | 715/11,344 (6·3%) | 1·96 (1·71-2·26) | <0·001* | 2·03 (1·77-2·33) | <0·001* | 2·02 (1·76-2·32) | <0·001* |
| Previous 15-90 days | | | |  |  |  |  |  |  |
|  | Influenza | 57/2,836 (2·0%) | 58/11,344 (0·5%) | 4·00 (2·77-5·77) | <0·001* | 3·81 (2·63-5·53) | <0·001* | 3·50 (2·41-5·10) | <0·001* |
|  | URI | 635/2,836 (22·4%) | 1,626/11,344 (14·3%) | 1·74 (1·57-1·92) | <0·001* | 1·80 (1·62-2·00) | <0·001* | 1·78 (1·60-1·97) | <0·001* |
| Previous 31-90 days | | | |  |  |  |  |  |  |
|  | Influenza | 53/2,836 (1·9%) | 52/11,344 (0·5%) | 4·14 (2·82-6·08) | <0·001* | 4·06 (2·76-5·98) | <0·001* | 3·79 (2·57-5·59) | <0·001* |
|  | URI | 507/2,836 (17·9%) | 1,417/11,344 (12·5%) | 1·53 (1·37-1·71) | <0·001* | 1·58 (1·41-1·76) | <0·001* | 1·55 (1·38-1·74) | <0·001* |
| The number of medical visit previous 1-365 days (Days, mean, SD) | | | | | |  |  |  |  |
|  | Influenza | 0·034 (0·21) | 0·014 (0·14) | 1·95 (1·55-2·45) | <0·001* | 1·90 (1·51-2·38) | <0·001* | 1·86 (1·48-2·33) | <0·001* |
|  | URI | 1·546 (3·43) | 1·159 (2·67) | 1·04 (1·03-1·06) | <0·001* | 1·05 (1·03-1·06) | <0·001* | 1·05 (1·03-1·06) | <0·001* |
| **Middle income (n = 16,625)** | | | |  |  |  |  |  |  |
| Previous 15-45 days | | | |  |  |  |  |  |  |
|  | Influenza | 16/3,325 (0·5%) | 31/13,300 (0·2%) | 2·07 (1·13-3·80) | 0·018* | 2·07 (1·13-3·79) | 0·019* | 1·73 (0·94-3·20) | 0·080 |
|  | URI | 376/3,325 (11·3%) | 785/13,300 (5·9%) | 2·04 (1·79-2·32) | <0·001* | 2·04 (1·79-2·33) | <0·001* | 2·03 (1·78-2·31) | <0·001* |
| Previous 15-90 days | | | |  |  |  |  |  |  |
|  | Influenza | 40/3,325 (1·2%) | 103/13,300 (0·8%) | 1·57 (1·08-2·26) | 0·017* | 1·58 (1·09-2·29) | 0·015* | 1·34 (0·93-1·95) | 0·122 |
|  | URI | 742/3,325 (22·3%) | 1,943/13,300 (14·6%) | 1·69 (1·54-1·86) | <0·001* | 1·68 (1·53-1·85) | <0·001* | 1·67 (1·52-1·84) | <0·001* |
| Previous 31-90 days | | | |  |  |  |  |  |  |
|  | Influenza | 38/3,325 (1·1%) | 84/13,300 (0·6%) | 1·83 (1·24-2·69) | 0·002* | 1·85 (1·26-2·73) | 0·002* | 1·63 (1·10-2·40) | 0·015* |
|  | URI | 613/3,325 (18·4%) | 1,702/13,300 (12·8%) | 1·55 (1·40-1·71) | <0·001* | 1·54 (1·39-1·71) | <0·001* | 1·53 (1·38-1·69) | <0·001* |
| The number of medical visit previous 1-365 days (Days, mean, SD) | | | | | |  |  |  |  |
|  | Influenza | 0·025 (0·18) | 0·022 (0·17) | 1·09 (0·88-1·35) | 0·449 | 1·05 (0·85-1·31) | 0·634 | 1·03 (0·83-1·28) | 0·790 |
|  | URI | 1·583 (3·17) | 1·235 (2·80) | 1·04 (1·03-1·05) | <0·001* | 1·04 (1·03-1·05) | <0·001* | 1·04 (1·03-1·05) | <0·001* |
| **High income (n = 9,454)** | | | |  |  |  |  |  |  |
| Previous 15-45 days | | | |  |  |  |  |  |  |
|  | Influenza | 6/1,909 (0·3%) | 14/7,636 (0·2%) | 1·72 (0·66-4·47) | 0·269 | 1·73 (0·66-4·53) | 0·261 | 1·35 (0·51-3·56) | 0·546 |
|  | URI | 210/1,909 (11·0%) | 457/7,636 (6·0%) | 1·95 (1·64-2·31) | <0·001* | 1·94 (1·63-2·31) | <0·001* | 1·93 (1·62-2·30) | <0·001* |
| Previous 15-90 days | | | |  |  |  |  |  |  |
|  | Influenza | 23/1,909 (1·2%) | 50/7,636 (0·7%) | 1·86 (1·13-3·07) | 0·015* | 1·82 (1·10-3·01) | 0·019* | 1·54 (0·92-2·56) | 0·098 |
|  | URI | 428/1,909 (22·4%) | 1,092/7,636 (14·3%) | 1·74 (1·54-1·98) | <0·001* | 1·74 (1·53-1·98) | <0·001* | 1·73 (1·52-1·96) | <0·001* |
| Previous 31-90 days | | | |  |  |  |  |  |  |
|  | Influenza | 21/1,909 (1·1%) | 45/7,636 (0·6%) | 1·89 (1·12-3·18) | 0·017* | 1·83 (1·08-3·10) | 0·024* | 1·60 (0·94-2·73) | 0·081 |
|  | URI | 352/1,909 (18·4%) | 957/7,636 (12·5%) | 1·59 (1·39-1·81) | <0·001* | 1·58 (1·38-1·81) | <0·001* | 1·56 (1·36-1·79) | <0·001* |
| The number of medical visit previous 1-365 days (Days, mean, SD) | | | | | |  |  |  |  |
|  | Influenza | 0·025 (0·18) | 0·020 (0·15) | 1·21 (0·90-1·62) | 0·219 | 1·18 (0·88-1·59) | 0·278 | 1·15 (0·85-1·55) | 0·378 |
|  | URI | 1·488 (2·49) | 1·223 (2·78) | 1·03 (1·02-1·05) | <0·001* | 1·04 (1·02-1·05) | <0·001* | 1·04 (1·02-1·05) | <0·001* |
| **CCI scores = 0 (n = 36,057)** | | | |  |  |  |  |  |  |
| Previous 15-45 days | | | |  |  |  |  |  |  |
|  | Influenza | 27/6,518 (0·4%) | 56/29,539 (0·2%) | 2·19 (1·38-3·47) | <0·001* | 2·14 (1·35-3·39) | 0·001* | 1·66 (1·04-2·66) | 0·034* |
|  | URI | 790/6,518 (12·1%) | 1,766/29,539 (6·0%) | 2·17 (1·99-2·37) | <0·001* | 2·18 (2·00-2·39) | <0·001* | 2·17 (1·99-2·37) | <0·001* |
| Previous 15-90 days | | | |  |  |  |  |  |  |
|  | Influenza | 92/6,518 (1·4%) | 196/29,539 (0·7%) | 2·14 (1·67-2·75) | <0·001* | 2·10 (1·64-2·69) | <0·001* | 1·74 (1·36-2·25) | <0·001* |
|  | URI | 1538/6,518 (23·6%) | 4,238/29,539 (14·3%) | 1·84 (1·73-1·97) | <0·001* | 1·85 (1·73-1·98) | <0·001* | 1·83 (1·71-1·96) | <0·001* |
| Previous 31-90 days | | | |  |  |  |  |  |  |
|  | Influenza | 86/6,518 (1·3%) | 170/29,539 (0·6%) | 2·31 (1·78-3·00) | <0·001* | 2·26 (1·74-2·94) | <0·001* | 1·95 (1·50-2·54) | <0·001* |
|  | URI | 1242/6,518 (19·1%) | 3,704/29,539 (12·5%) | 1·64 (1·53-1·76) | <0·001* | 1·65 (1·54-1·77) | <0·001* | 1·63 (1·51-1·75) | <0·001* |
| The number of medical visit previous 1-365 days (Days, mean, SD) | | | |  |  |  |  |  |  |
|  | Influenza | 0·027 (0·19) | 0·019 (0·16) | 1·33 (1·15-1·54) | <0·001* | 1·30 (1·13-1·51) | <0·001* | 1·24 (1·07-1·44) | 0·004* |
|  | URI | 1·590 (3·01) | 1·195 (2·74) | 1·04 (1·03-1·05) | <0·001* | 1·04 (1·04-1·05) | <0·001* | 1·04 (1·03-1·05) | <0·001* |
| **CCI scores = 1 (n = 2,305)** | | | |  |  |  |  |  |  |
| Previous 15-45 days | | | |  |  |  |  |  |  |
|  | Influenza | 4/889 (0·4%) | 4/1,416 (0·3%) | 1·60 (0·40-6·40) | 0·510 | 1·64 (0·41-6·63) | 0·486 | 1·64 (0·40-6·63) | 0·491 |
|  | URI | 88/889 (9·9%) | 109/1,416 (7·7%) | 1·32 (0·98-1·77) | 0·067 | 1·33 (0·99-1·79) | 0·058 | 1·33 (0·99-1·79) | 0·058 |
| Previous 15-90 days | | | |  |  |  |  |  |  |
|  | Influenza | 17/889 (1·9%) | 9/1,416 (0·6%) | 3·05 (1·35-6·87) | 0·007* | 3·07 (1·36-6·94) | 0·007* | 3·07 (1·36-6·94) | 0·007* |
|  | URI | 171/889 (19·2%) | 234/1,416 (16·5%) | 1·20 (0·97-1·50) | 0·096 | 1·22 (0·98-1·53) | 0·071 | 1·22 (0·98-1·53) | 0·073 |
| Previous 31-90 days | | | |  |  |  |  |  |  |
|  | Influenza | 17/889 (1·9%) | 7/1,416 (0·5%) | 3·92 (1·62-9·50) | 0·002* | 3·93 (1·62-9·52) | 0·003* | 3·96 (1·63-9·62) | 0·002* |
|  | URI | 149/889 (16·8%) | 200/1,416 (14·1%) | 1·22 (0·97-1·54) | 0·086 | 1·25 (0·99-1·59) | 0·057 | 1·26 (1·00-1·59) | 0·054 |
| The number of medical visit previous 1-365 days (Days, mean, SD) | | | |  |  |  |  |  |  |
|  | Influenza | 0·025 (0·16) | 0·020 (0·18) | 1·18 (0·73-1·92) | 0·497 | 1·17 (0·72-1·89) | 0·534 | 1·16 (0·72-1·88) | 0·546 |
|  | URI | 1·620 (4·15) | 1·474 (3·17) | 1·01 (0·99-1·04) | 0·344 | 1·01 (0·99-1·04) | 0·298 | 1·01 (0·99-1·04) | 0·303 |
| **CCI scores ≥ 2 (n = 1,988)** | | | |  |  |  |  |  |  |
| Previous 15-45 days | | | |  |  |  |  |  |  |
|  | Influenza | 4/663 (0·6%) | 2/1,325 (0·2%) | 4·01 (0·73-21·91) | 0·110 | 3·88 (0·70-21·34) | 0·120 | 3·85 (0·70-21·19) | 0·122 |
|  | URI | 38/663 (5·7%) | 82/1,325 (6·2%) | 0·92 (0·62-1·37) | 0·687 | 0·89 (0·60-1·33) | 0·575 | 0·90 (0·60-1·34) | 0·593 |
| Previous 15-90 days | | | |  |  |  |  |  |  |
|  | Influenza | 11/663 (1·7%) | 6/1,325 (0·5%) | 3·71 (1·37-10·07) | 0·010* | 3·85 (1·41-10·50) | 0·008* | 3·85 (1·41-10·49) | 0·009* |
|  | URI | 96/663 (14·5%) | 189/1,325 (14·3%) | 1·02 (0·78-1·33) | 0·897 | 1·03 (0·79-1·34) | 0·853 | 1·02 (0·78-1·33) | 0·900 |
| Previous 31-90 days | | | |  |  |  |  |  |  |
|  | Influenza | 9/663 (1·4%) | 4/1,325 (0·3%) | 4·55 (1·39-14·81) | 0·012* | 4·88 (1·49-15·95) | 0·009* | 4·91 (1·50-16·06) | 0·009* |
|  | URI | 81/663 (12·2%) | 172/1,325 (13·0%) | 0·93 (0·70-1·24) | 0·630 | 0·94 (0·70-1·24) | 0·649 | 0·93 (0·70-1·23) | 0·602 |
| The number of medical visit previous 1-365 days (Days, mean, SD) | | | |  |  |  |  |  |  |
|  | Influenza | 0·038 (0·25) | 0·011 (0·13) | 2·43 (1·32-4·45) | 0·004* | 2·40 (1·29-4·44) | 0·006* | 2·40 (1·29-4·44) | 0·006* |
|  | URI | 1·035 (2·47) | 1·166 (2·52) | 0·98 (0·94-1·02) | 0·272 | 0·98 (0·94-1·02) | 0·293 | 0·98 (0·94-1·02) | 0·289 |
| **Non-asthma (n = 37,033)** | | | |  |  |  |  |  |  |
| Previous 15-45 days | | | |  |  |  |  |  |  |
|  | Influenza | 28/7,366 (0·4%) | 51/29,667 (0·2%) | 2·22 (1·40-3·52) | <0·001* | 2·18 (1·37-3·47) | 0·001* | 1·76 (1·10-2·82) | 0·019* |
|  | URI | 803/7,366 (10·9%) | 1,679/29,667 (5·7%) | 2·04 (1·87-2·23) | <0·001* | 2·07 (1·89-2·26) | <0·001* | 2·06 (1·88-2·25) | <0·001* |
| Previous 15-90 days | | | |  |  |  |  |  |  |
|  | Influenza | 110/7,366 (1·5%) | 179/29,667 (0·6%) | 2·50 (1·97-3·17) | <0·001* | 2·49 (1·96-3·16) | <0·001* | 2·17 (1·70-2·76) | <0·001* |
|  | URI | 1584/7,366 (21·5%) | 4,045/29,667 (13·6%) | 1·74 (1·63-1·85) | <0·001* | 1·76 (1·65-1·88) | <0·001* | 1·74 (1·63-1·86) | <0·001* |
| Previous 31-90 days | | | |  |  |  |  |  |  |
|  | Influenza | 103/7,366 (1·4%) | 155/29,667 (0·5%) | 2·70 (2·10-3·47) | <0·001* | 2·71 (2·11-3·49) | <0·001* | 2·44 (1·89-3·14) | <0·001* |
|  | URI | 1283/7,366 (17·4%) | 3,529/29,667 (11·9%) | 1·56 (1·46-1·68) | <0·001* | 1·58 (1·47-1·70) | <0·001* | 1·56 (1·45-1·67) | <0·001* |
| The number of medical visit previous 1-365 days (Days, mean, SD) | | | |  |  |  |  |  |  |
|  | Influenza | 0·027 (0·18) | 0·017 (0·15) | 1·40 (1·21-1·62) | <0·001* | 1·40 (1·21-1·62) | <0·001* | 1·34 (1·16-1·55) | <0·001* |
|  | URI | 1·422 (2·82) | 1·091 (2·42) | 1·05 (1·04-1·06) | <0·001* | 1·05 (1·04-1·06) | <0·001* | 1·05 (1·04-1·06) | <0·001* |
| **Asthma (n = 3,317)** | | | |  |  |  |  |  |  |
| Previous 15-45 days | | | |  |  |  |  |  |  |
|  | Influenza | 7/704 (1·0%) | 11/2,613 (0·4%) | 2·38 (0·92-6·15) | 0·075 | 2·31 (0·88-6·03) | 0·088 | 2·14 (0·82-5·60) | 0·123 |
|  | URI | 113/704 (16·1%) | 278/2,613 (10·6%) | 1·61 (1·27-2·04) | <0·001* | 1·65 (1·30-2·10) | <0·001* | 1·64 (1·29-2·09) | <0·001* |
| Previous 15-90 days | | | |  |  |  |  |  |  |
|  | Influenza | 10/704 (1·4%) | 32/2,613 (1·2%) | 1·16 (0·57-2·38) | 0·680 | 1·15 (0·56-2·36) | 0·704 | 0·99 (0·48-2·04) | 0·972 |
|  | URI | 221/704 (31·4%) | 616/2,613 (23·6%) | 1·48 (1·24-1·78) | <0·001* | 1·52 (1·26-1·82) | <0·001* | 1·52 (1·26-1·83) | <0·001* |
| Previous 31-90 days | | | |  |  |  |  |  |  |
|  | Influenza | 9/704 (1·3%) | 26/2,613 (1·0%) | 1·29 (0·60-2·76) | 0·515 | 1·27 (0·59-2·75) | 0·538 | 1·12 (0·52-2·43) | 0·773 |
|  | URI | 189/704 (26·8%) | 547/2,613 (20·9%) | 1·39 (1·15-1·68) | <0·001* | 1·41 (1·17-1·72) | <0·001* | 1·41 (1·16-1·71) | <0·001* |
| The number of medical visit previous 1-365 days (Days, mean, SD) | | | |  |  |  |  |  |  |
|  | Influenza | 0·043 (0·27) | 0·036 (0·22) | 1·13 (0·81-1·60) | 0·472 | 1·08 (0·76-1·54) | 0·664 | 1·07 (0·75-1·52) | 0·717 |
|  | URI | 2·857 (5·20) | 2·507 (5·00) | 1·01 (1·00-1·03) | 0·106 | 1·01 (1·00-1·03) | 0·071 | 1·01 (1·00-1·03) | 0·073 |
| **Non-COPD (n = 39,203)** | | | |  |  |  |  |  |  |
| Previous 15-45 days | | | |  |  |  |  |  |  |
|  | Influenza | 30/7,806 (0·4%) | 60/31,397 (0·2%) | 2·02 (1·30-3·13) | 0·002* | 1·98 (1·27-3·08) | 0·003* | 1·59 (1·02-2·49) | 0·042* |
|  | URI | 882/7,806 (11·3%) | 1,868/31,397 (5·9%) | 2·01 (1·85-2·19) | <0·001* | 2·05 (1·88-2·23) | <0·001* | 2·04 (1·87-2·22) | <0·001* |
| Previous 15-90 days | | | |  |  |  |  |  |  |
|  | Influenza | 112/7,806 (1·4%) | 205/31,397 (0·7%) | 2·22 (1·76-2·79) | <0·001* | 2·20 (1·74-2·78) | <0·001* | 1·89 (1·49-2·39) | <0·001* |
|  | URI | 1740/7,806 (22·3%) | 4,470/31,397 (14·2%) | 1·73 (1·62-1·84) | <0·001* | 1·76 (1·65-1·87) | <0·001* | 1·74 (1·63-1·85) | <0·001* |
| Previous 31-90 days | | | |  |  |  |  |  |  |
|  | Influenza | 104/7,806 (1·3%) | 175/31,397 (0·6%) | 2·41 (1·89-3·08) | <0·001* | 2·42 (1·89-3·09) | <0·001* | 2·14 (1·67-2·74) | <0·001* |
|  | URI | 1419/7,806 (18·2%) | 3,903/31,397 (12·4%) | 1·57 (1·47-1·67) | <0·001* | 1·58 (1·48-1·69) | <0·001* | 1·56 (1·46-1·67) | <0·001* |
| The number of medical visit previous 1-365 days (Days, mean, SD) | | | |  |  |  |  |  |  |
|  | Influenza | 0·028 (0·19) | 0·019 (0·15) | 1·35 (1·18-1·55) | <0·001* | 1·34 (1·16-1·53) | <0·001* | 1·28 (1·12-1·47) | <0·001* |
|  | URI | 1·548 (3·15) | 1·185 (2·73) | 1·04 (1·03-1·05) | <0·001* | 1·04 (1·03-1·05) | <0·001* | 1·04 (1·03-1·05) | <0·001* |
| **COPD (n = 1,147)** | | | |  |  |  |  |  |  |
| Previous 15-45 days | | | |  |  |  |  |  |  |
|  | Influenza | 5/264 (1·9%) | 2/883 (0·2%) | 8·50 (1·64-44·09) | 0·011* | 8·16 (1·56-42·65) | 0·013* | 8·48 (1·62-44·33) | 0·011* |
|  | URI | 34/264 (12·9%) | 89/883 (10·1%) | 1·32 (0·87-2·01) | 0·198 | 1·30 (0·85-1·99) | 0·231 | 1·33 (0·87-2·04) | 0·194 |
| Previous 15-90 days | | | |  |  |  |  |  |  |
|  | Influenza | 8/264 (3·0%) | 6/883 (0·7%) | 4·57 (1·57-13·28) | 0·005* | 4·74 (1·62-13·86) | 0·005* | 4·70 (1·61-13·76) | 0·005* |
|  | URI | 65/264 (24·6%) | 191/883 (21·6%) | 1·18 (0·86-1·63) | 0·306 | 1·22 (0·88-1·69) | 0·235 | 1·22 (0·87-1·69) | 0·247 |
| Previous 31-90 days | | | |  |  |  |  |  |  |
|  | Influenza | 8/264 (3·0%) | 6/883 (0·7%) | 4·57 (1·57-13·28) | 0·005* | 4·74 (1·62-13·86) | 0·005* | 4·72 (1·61-13·81) | 0·005* |
|  | URI | 53/264 (20·1%) | 173/883 (19·6%) | 1·03 (0·73-1·45) | 0·862 | 1·07 (0·75-1·51) | 0·722 | 1·06 (0·74-1·50) | 0·766 |
| The number of medical visit previous 1-365 days (Days, mean, SD) | | | |  |  |  |  |  |  |
|  | Influenza | 0·022 (0·18) | 0·022 (0·18) | 1·53 (0·79-2·97) | 0·213 | 1·57 (0·81-3·07) | 0·185 | 1·57 (0·80-3·06) | 0·190 |
|  | URI | 1·931 (3·43) | 1·931 (3·43) | 0·96 (0·91-1·00) | 0·070 | 0·96 (0·91-1·01) | 0·076 | 0·96 (0·91-1·01) | 0·077 |
| **Non-hypertension (n = 32,265)** | | | |  |  |  |  |  |  |
| Previous 15-45 days | | | |  |  |  |  |  |  |
|  | Influenza | 30/6,413 (0·5%) | 53/25,852 (0·2%) | 2·29 (1·46-3·58) | <0·001* | 2·24 (1·43-3·52) | <0·001* | 1·75 (1·11-2·78) | 0·017* |
|  | URI | 757/6,413 (11·8%) | 1,477/25,852 (5·7%) | 2·21 (2·01-2·42) | <0·001* | 2·23 (2·03-2·44) | <0·001* | 2·21 (2·01-2·42) | <0·001* |
| Previous 15-90 days | | | |  |  |  |  |  |  |
|  | Influenza | 99/6,413 (1·5%) | 189/25,852 (0·7%) | 2·13 (1·67-2·72) | <0·001* | 2·11 (1·65-2·70) | <0·001* | 1·78 (1·38-2·28) | <0·001* |
|  | URI | 1471/6,413 (22·9%) | 3,633/25,852 (14·1%) | 1·82 (1·70-1·95) | <0·001* | 1·84 (1·72-1·97) | <0·001* | 1·82 (1·69-1·94) | <0·001* |
| Previous 31-90 days | | | |  |  |  |  |  |  |
|  | Influenza | 92/6,413 (1·4%) | 164/25,852 (0·6%) | 2·28 (1·76-2·95) | <0·001* | 2·28 (1·76-2·95) | <0·001* | 1·98 (1·52-2·56) | <0·001* |
|  | URI | 1194/6,413 (18·6%) | 3,163/25,852 (12·2%) | 1·64 (1·53-1·77) | <0·001* | 1·65 (1·54-1·78) | <0·001* | 1·63 (1·51-1·75) | <0·001* |
| The number of medical visit previous 1-365 days (Days, mean, SD) | | | |  |  |  |  |  |  |
|  | Influenza | 0·030 (0·20) | 0·021 (0·17) | 1·30 (1·13-1·50) | <0·001* | 1·29 (1·12-1·49) | <0·001* | 1·23 (1·07-1·42) | <0·001* |
|  | URI | 1·557 (2·92) | 1·164 (2·62) | 1·05 (1·04-1·06) | <0·001* | 1·05 (1·04-1·06) | <0·001* | 1·05 (1·04-1·06) | <0·001* |
| **Hypertension (n = 8,085)** | | | |  |  |  |  |  |  |
| Previous 15-45 days | | | |  |  |  |  |  |  |
|  | Influenza | 5/1,657 (0·3%) | 9/6,428 (0·1%) | 2·17 (0·73-6·47) | 0·166 | 1·97 (0·65-5·97) | 0·234 | 1·94 (0·64-5·89) | 0·241 |
|  | URI | 159/1,657 (9·6%) | 480/6,428 (7·5%) | 1·32 (1·09-1·59) | 0·004* | 1·35 (1·11-1·63) | 0·002* | 1·35 (1·11-1·63) | 0·003* |
| Previous 15-90 days | | | |  |  |  |  |  |  |
|  | Influenza | 21/1,657 (1·3%) | 22/6,428 (0·3%) | 3·74 (2·05-6·81) | <0·001* | 3·63 (1·97-6·67) | <0·001* | 3·55 (1·93-6·54) | <0·001* |
|  | URI | 334/1,657 (20·2%) | 1028/6,428 (16·0%) | 1·33 (1·16-1·52) | <0·001* | 1·37 (1·19-1·58) | <0·001* | 1·37 (1·19-1·57) | <0·001* |
| Previous 31-90 days | | | |  |  |  |  |  |  |
|  | Influenza | 20/1,657 (1·2%) | 17/6,428 (0·3%) | 4·61 (2·41-8·82) | <0·001* | 4·59 (2·38-8·86) | <0·001* | 4·56 (2·36-8·81) | <0·001* |
|  | URI | 278/1,657 (16·8%) | 913/6,428 (14·2%) | 1·22 (1·05-1·41) | 0·009* | 1·24 (1·07-1·44) | 0·005* | 1·24 (1·07-1·44) | 0·005* |
| The number of medical visit previous 1-365 days (Days, mean, SD) | | | |  |  |  |  |  |  |
|  | Influenza | 0·021 (0·16) | 0·009 (0·10) | 2·02 (1·36-3·00) | 0·001* | 1·87 (1·24-2·81) | 0·003* | 1·86 (1·23-2·80) | 0·003* |
|  | URI | 1·511 (3·82) | 1·372 (3·23) | 1·01 (1·00-1·03) | 0·135 | 1·02 (1·00-1·03) | 0·060 | 1·01 (1·00-1·03) | 0·063 |

Abbreviations: COPD, Chronic obstructive pulmonary disease; Upper respiratory tract infection, URI; COVID-19, Coronavirus Disease 2019; N/A, Not applicable; SD, Standard deviation

* Conditional or unconditional logistic regression model, Significance at P < 0·05

† Model 1 was adjusted for age, sex, income, CCI scores, asthma, COPD, and hypertension

‡ Model 2 was adjusted for model 1 plus influenza and URI
