# Supplementary material for: The associations of previous influenza/upper respiratory infection with COVID-19 susceptibility/morbidity/mortality: a nationwide cohort study in South Korea
Source: Sci Rep. 2021 Nov 3;11:21568. doi: 10.1038/s41598-021-00428-x (PMC8566493; doi:10.1038/s41598-021-00428-x)
Supplement: Supplementary file 5 — Supplementary Information 5. [file 41598_2021_428_MOESM5_ESM.docx]

**Table S5** Subgroup analyses of crude and adjusted odds ratios of influenza and URI (previous 1-14, 1-30, and 1-90 days) for morbidity in COVID-19 participants by covariates

| Characteristics | | Severe participants | Mild participants | ORs (95% confidence interval) for morbidity | | | | | |
| --- | --- | --- | --- | --- | --- | --- | --- | --- | --- |
|  |  | (exposure/total, %) | (exposure/total, %) | Crude | P-value | Model 1† | P-value | Model 2†‡ | P-value |
| **Age < 50 years old ( n = 4,282)** | | | |  |  |  |  |  |  |
| Previous 1-14 days | | | |  |  |  |  |  |  |
|  | Influenza | 1/98 (1·0%) | 13/4,184 (0·3%) | 3·68 (1·39-30·03) | 0·013* | 3·89 (0·78-19·41) | 0·097 | 3·77 (0·75-18·94) | 0·107 |
|  | URI | 23/98 (23·5%) | 734/4,184 (17·5%) | 1·52 (0·96-2·43) | 0·077 | 1·56 (0·97-2·50) | 0·067 | 1·55 (0·96-2·48) | 0·072 |
| Previous 1-30 days | | | |  |  |  |  |  |  |
|  | Influenza | 1/98 (1·0%) | 15/4,184 (0·4%) | 2·87 (0·38-21·91) | 0·310 | 1·49 (0·17-12·81) | 0·715 | 1·46 (0·17-12·51) | 0·731 |
|  | URI | 24/98 (24·5%) | 914/4,184 (21·8%) | 1·16 (0·73-1·85) | 0·532 | 1·17 (0·73-1·88) | 0·516 | 1·17 (0·73-1·87) | 0·523 |
| Previous 1-90 days | | | |  |  |  |  |  |  |
|  | Influenza | 2/98 (2·0%) | 79/4,184 (1·9%) | 1·08 (0·26-4·47) | 0·913 | 0·80 (0·19-3·36) | 0·757 | 0·78 (0·18-3·27) | 0·730 |
|  | URI | 39/98 (39·8%) | 1,434/4,184 (34·3%) | 1·27 (0·84-1·91) | 0·256 | 1·21 (0·80-1·84) | 0·367 | 1·22 (0·80-1·85) | 0·359 |
| **Age ≥ 50 years old (n = 3,788)** | | | |  |  |  |  |  |  |
| Previous 1-14 days | | | |  |  |  |  |  |  |
|  | Influenza | 5/471 (1·1%) | 8/3,317 (0·2%) | 4·44 (1·45-13·62) | 0·009* | 4·82 (1·42-16·31) | 0·012* | 4·35 (1·26-15·04) | 0·020* |
|  | URI | 85/471 (18·0%) | 599/3,317 (18·1%) | 1·00 (0·78-1·28) | 0·995 | 1·40 (1·06-1·84) | 0·017* | 1·37 (1·04-1·80) | 0·027* |
| Previous 1-30 days | | | |  |  |  |  |  |  |
|  | Influenza | 6/471 (1·3%) | 10/3,317 (0·3%) | 4·27 (1·54-11·80) | 0·005* | 4·76 (1·60-14·16) | 0·005* | 4·40 (1·46-13·28) | 0·009* |
|  | URI | 95/471 (20·2%) | 708/3,317 (21·3%) | 0·93 (0·73-1·18) | 0·560 | 1·35 (1·04-1·76) | 0·025* | 1·32 (1·01-1·73) | 0·039* |
| Previous 1-90 days | | | |  |  |  |  |  |  |
|  | Influenza | 12/471 (2·5%) | 53/3,317 (1·6%) | 1·61 (0·85-3·04) | 0·141 | 1·87 (0·93-3·76) | 0·078 | 1·85 (0·92-3·71) | 0·084 |
|  | URI | 138/471 (29·3%) | 1,083/3,317 (32·6%) | 0·86 (0·69-1·06) | 0·146 | 1·16 (0·91-1·46) | 0·230 | 1·15 (0·91-1·45) | 0·248 |
| **Men (n = 3,236)** | | | |  |  |  |  |  |  |
| Previous 1-14 days | | | |  |  |  |  |  |  |
|  | Influenza | 4/306 (1·3%) | 8/2,930 (0·3%) | 4·12 (1·21-12·41) | 0·023* | 4·74 (1·25-17·93) | 0·022* | 3·74 (0·95-14·72) | 0·059 |
|  | URI | 64/306 (20·9%) | 477/2,930 (16·3%) | 1·39 (1·04-1·86) | 0·027* | 1·80 (1·30-2·50) | <0·001* | 1·75 (1·26-2·44) | 0·001* |
| Previous 1-30 days | | | |  |  |  |  |  |  |
|  | Influenza | 5/306 (1·6%) | 8/2,930 (0·3%) | 6·07 (1·97-18·66) | 0·002* | 6·82 (1·92-24·16) | 0·003* | 5·72 (1·59-20·65) | 0·008* |
|  | URI | 68/306 (22·2%) | 559/2,930 (19·1%) | 1·21 (0·91-1·61) | 0·186 | 1·60 (1·16-2·20) | 0·004* | 1·54 (1·11-2·13) | 0·009* |
| Previous 1-90 days | | | |  |  |  |  |  |  |
|  | Influenza | 8/306 (2·6%) | 55/2,930 (1·9%) | 1·40 (0·66-2·98) | 0·375 | 1·74 (0·77-3·95) | 0·185 | 1·69 (0·75-3·82) | 0·209 |
|  | URI | 97/306 (31·7%) | 874/2,930 (29·8%) | 1·09 (0·85-1·41) | 0·497 | 1·30 (0·97-1·73) | 0·078 | 1·29 (0·97-1·72) | 0·085 |
| **Women (n = 4,834)** | | | |  |  |  |  |  |  |
| Previous 1-14 days | | | |  |  |  |  |  |  |
|  | Influenza | 2/263 (0·8%) | 13/4,571 (0·3%) | 3·00 (1·33-17·27) | 0·017* | 5·92 (1·51-23·24) | 0·011* | 5·91 (1·50-23·29) | 0·011* |
|  | URI | 44/263 (16·7%) | 856/4,571 (18·7%) | 0·87 (0·63-1·22) | 0·419 | 1·12 (0·79-1·60) | 0·517 | 1·12 (0·79-1·60) | 0·533 |
| Previous 1-30 days | | | |  |  |  |  |  |  |
|  | Influenza | 2/263 (0·8%) | 17/4,571 (0·4%) | 2·05 (0·47-8·93) | 0·338 | 2·02 (0·44-9·23) | 0·364 | 2·00 (0·44-9·17) | 0·372 |
|  | URI | 51/263 (19·4%) | 1,063/4,571 (23·3%) | 0·79 (0·58-1·09) | 0·149 | 1·07 (0·77-1·50) | 0·681 | 1·07 (0·76-1·50) | 0·699 |
| Previous 1-90 days | | | |  |  |  |  |  |  |
|  | Influenza | 6/263 (2·3%) | 77/4,571 (1·7%) | 1·36 (0·59-3·16) | 0·469 | 1·51 (0·60-3·81) | 0·387 | 1·50 (0·59-3·79) | 0·396 |
|  | URI | 80/263 (30·4%) | 1,643/4,571 (35·9%) | 0·78 (0·60-1·02) | 0·070 | 1·07 (0·80-1·43) | 0·656 | 1·06 (0·79-1·43) | 0·678 |
| **Low income (n = 2,836)** | | | |  |  |  |  |  |  |
| Previous 1-14 days | | | |  |  |  |  |  |  |
|  | Influenza | 2/185 (1·1%) | 6/2,651 (0·2%) | 4·78 (1·12-30·03) | 0·037* | 7·30 (1·22-43·83) | 0·030* | 6·52 (1·04-40·83) | 0·045* |
|  | URI | 28/185 (15·1%) | 426/2,651 (16·1%) | 0·93 (0·62-1·41) | 0·738 | 1·37 (0·88-2·15) | 0·167 | 1·32 (0·84-2·08) | 0·233 |
| Previous 1-30 days | | | |  |  |  |  |  |  |
|  | Influenza | 3/185 (1·6%) | 9/2,651 (0·3%) | 4·84 (1·30-18·03) | 0·019* | 5·23 (1·26-21·67) | 0·023* | 5·03 (1·20-21·14) | 0·027* |
|  | URI | 31/185 (16·8%) | 532/2,651 (20·1%) | 0·80 (0·54-1·19) | 0·276 | 1·28 (0·83-1·98) | 0·260 | 1·25 (0·81-1·94) | 0·306 |
| Previous 1-90 days | | | |  |  |  |  |  |  |
|  | Influenza | 7/185 (3·8%) | 57/2,651 (2·2%) | 1·79 (0·81-3·98) | 0·154 | 1·74 (0·74-4·11) | 0·206 | 1·74 (0·74-4·09) | 0·208 |
|  | URI | 48/185 (25·9%) | 846/2,651 (31·9%) | 0·75 (0·53-1·05) | 0·092 | 1·18 (0·81-1·72) | 0·379 | 1·18 (0·81-1·72) | 0·382 |
| **Middle income (n = 3,325)** | | | |  |  |  |  |  |  |
| Previous 1-14 days | | | |  |  |  |  |  |  |
|  | Influenza | 1/211 (0·5%) | 10/3,114 (0·3%) | 1·46 (0·71-15·38) | 0·128 | 5·27 (1·03-27·00) | 0·046* | 4·85 (0·92-25·65) | 0·063 |
|  | URI | 50/211 (23·7%) | 604/3,114 (19·4%) | 1·33 (0·96-1·84) | 0·092 | 1·65 (1·16-2·37) | 0·006* | 1·64 (1·14-2·34) | 0·007* |
| Previous 1-30 days | | | |  |  |  |  |  |  |
|  | Influenza | 1/211 (0·5%) | 11/3,114 (0·4%) | 1·34 (0·17-10·46) | 0·778 | 1·97 (0·24-16·43) | 0·530 | 1·80 (0·21-15·27) | 0·588 |
|  | URI | 53/211 (25·1%) | 717/3,114 (23·0%) | 1·12 (0·81-1·55) | 0·486 | 1·50 (1·05-2·13) | 0·025* | 1·49 (1·05-2·12) | 0·026* |
| Previous 1-90 days | | | |  |  |  |  |  |  |
|  | Influenza | 2/211 (0·9%) | 49/3,114 (1·6%) | 0·60 (0·15-2·48) | 0·479 | 1·05 (0·24-4·63) | 0·954 | 1·01 (0·23-4·43) | 0·995 |
|  | URI | 74/211 (35·1%) | 1,086/3,114 (34·9%) | 1·01 (0·75-1·35) | 0·954 | 1·29 (0·93-1·78) | 0·126 | 1·29 (0·93-1·78) | 0·126 |
| **High income (n = 1,909)** | | | |  |  |  |  |  |  |
| Previous 1-14 days | | | |  |  |  |  |  |  |
|  | Influenza | 3/173 (1·7%) | 5/1,736 (0·3%) | 5·48 (1·12-17·01) | 0·044* | 3·47 (0·73-16·48) | 0·118 | 3·35 (0·69-16·17) | 0·133 |
|  | URI | 30/173 (17·3%) | 303/1,736 (17·5%) | 0·99 (0·66-1·50) | 0·970 | 1·23 (0·78-1·94) | 0·379 | 1·20 (0·76-1·91) | 0·431 |
| Previous 1-30 days | | | |  |  |  |  |  |  |
|  | Influenza | 3/173 (1·7%) | 5/1,736 (0·3%) | 6·11 (1·45-25·79) | 0·014* | 3·84 (0·77-19·23) | 0·101 | 3·71 (0·73-18·77) | 0·114 |
|  | URI | 35/173 (20·2%) | 373/1,736 (21·5%) | 0·93 (0·63-1·37) | 0·701 | 1·13 (0·74-1·74) | 0·573 | 1·09 (0·71-1·69) | 0·686 |
| Previous 1-90 days | | | |  |  |  |  |  |  |
|  | Influenza | 5/173 (2·9%) | 26/1,736 (1·5%) | 1·96 (0·74-5·16) | 0·175 | 1·81 (0·60-5·49) | 0·295 | 1·78 (0·59-5·43) | 0·309 |
|  | URI | 55/173 (31·8%) | 585/1,736 (33·7%) | 0·92 (0·66-1·28) | 0·613 | 1·09 (0·75-1·58) | 0·670 | 1·07 (0·74-1·56) | 0·720 |
| **CCI scores = 0 (n = 6,518)** | | | |  |  |  |  |  |  |
| Previous 1-14 days | | | |  |  |  |  |  |  |
|  | Influenza | 4/264 (1·5%) | 18/6,254 (0·3%) | 5·33 (1·79-15·86) | 0·003* | 4·13 (1·25-13·66) | 0·020* | 3·97 (1·17-13·44) | 0·027* |
|  | URI | 69/264 (26·1%) | 1,158/6,254 (18·5%) | 1·56 (1·18-2·06) | 0·002* | 1·49 (1·11-2·01) | 0·008* | 1·48 (1·10-1·99) | 0·010* |
| Previous 1-30 days | | | |  |  |  |  |  |  |
|  | Influenza | 4/264 (1·5%) | 20/6,254 (0·3%) | 4·80 (1·63-14·13) | 0·005* | 3·86 (1·19-12·53) | 0·025* | 3·65 (1·10-12·06) | 0·034* |
|  | URI | 77/264 (29·2%) | 1,418/6,254 (22·7%) | 1·40 (1·07-1·84) | 0·014* | 1·34 (1·01-1·78) | 0·046* | 1·32 (0·99-1·76) | 0·058 |
| Previous 1-90 days | | | |  |  |  |  |  |  |
|  | Influenza | 6/264 (2·3%) | 106/6,254 (1·7%) | 1·35 (0·59-3·10) | 0·479 | 1·33 (0·56-3·18) | 0·523 | 1·29 (0·54-3·09) | 0·568 |
|  | URI | 105/264 (39·8%) | 2,186/6,254 (35·0%) | 1·23 (0·96-1·58) | 0·109 | 1·15 (0·88-1·50) | 0·316 | 1·14 (0·87-1·49) | 0·335 |
| **CCI scores = 1 (n = 889)** | | | |  |  |  |  |  |  |
| Previous 1-14 days | | | |  |  |  |  |  |  |
|  | Influenza | 1/134 (0·7%) | 1/755 (0·1%) | 5·67 (0·35-91·27) | 0·221 | 2·64 (0·16-44·81) | 0·501 | 2·21 (0·13-36·43) | 0·579 |
|  | URI | 25/134 (18·7%) | 122/755 (16·2%) | 1·25 (0·78-2·00) | 0·354 | 1·67 (1·00-2·78) | 0·051 | 1·65 (0·99-2·76) | 0·055 |
| Previous 1-30 days | | | |  |  |  |  |  |  |
|  | Influenza | 1/134 (0·7%) | 1/755 (0·1%) | 5·67 (0·35-91·27) | 0·221 | 2·64 (0·16-44·81) | 0·501 | 2·29 (0·14-37·72) | 0·562 |
|  | URI | 27/134 (20·1%) | 142/755 (18·8%) | 1·09 (0·69-1·73) | 0·715 | 1·57 (0·95-2·60) | 0·082 | 1·56 (0·94-2·58) | 0·088 |
| Previous 1-90 days | | | |  |  |  |  |  |  |
|  | Influenza | 4/134 (3·0%) | 15/755 (2·0%) | 1·52 (0·50-4·65) | 0·463 | 2·19 (0·63-7·59) | 0·216 | 2·30 (0·66-7·99) | 0·191 |
|  | URI | 40/134 (29·9%) | 222/755 (29·4%) | 1·02 (0·68-1·53) | 0·917 | 1·31 (0·84-2·04) | 0·231 | 1·33 (0·85-2·08) | 0·208 |
| **CCI scores ≥ 2 (n = 663)** | | | |  |  |  |  |  |  |
| Previous 1-14 days | | | |  |  |  |  |  |  |
|  | Influenza | 1/171 (0·6%) | 2/492 (0·4%) | 1·70 (0·41-20·71) | 0·290 | 7·03 (0·86-57·28) | 0·069 | 7·39 (0·87-63·20) | 0·068 |
|  | URI | 14/171 (8·2%) | 53/492 (10·8%) | 0·74 (0·40-1·37) | 0·336 | 1·04 (0·53-2·04) | 0·911 | 0·93 (0·46-1·85) | 0·828 |
| Previous 1-30 days | | | |  |  |  |  |  |  |
|  | Influenza | 2/171 (1·2%) | 4/492 (0·8%) | 1·44 (0·26-7·96) | 0·673 | 3·50 (0·59-20·76) | 0·168 | 3·55 (0·59-21·47) | 0·167 |
|  | URI | 15/171 (8·8%) | 62/492 (12·6%) | 0·67 (0·37-1·21) | 0·181 | 1·01 (0·53-1·94) | 0·969 | 0·96 (0·50-1·85) | 0·903 |
| Previous 1-90 days | | | |  |  |  |  |  |  |
|  | Influenza | 4/171 (2·3%) | 11/492 (2·2%) | 1·05 (0·33-3·34) | 0·937 | 1·50 (0·43-5·26) | 0·523 | 1·48 (0·42-5·18) | 0·544 |
|  | URI | 32/171 (18·7%) | 109/492 (22·2%) | 0·81 (0·52-1·26) | 0·344 | 1·24 (0·76-2·03) | 0·390 | 1·23 (0·76-2·02) | 0·403 |
| **Non-asthma (n =7,366 )** | | | |  |  |  |  |  |  |
| Previous 1-14 days | | | |  |  |  |  |  |  |
|  | Influenza | 5/483 (1·0%) | 19/6,883 (0·3%) | 3·83 (1·71-10·43) | 0·001* | 4·13 (1·48-11·54) | 0·007* | 3·82 (1·34-10·91) | 0·012* |
|  | URI | 91/483 (18·8%) | 1,207/6,883 (17·5%) | 1·11 (0·87-1·40) | 0·400 | 1·47 (1·14-1·91) | 0·003* | 1·45 (1·12-1·88) | 0·005* |
| Previous 1-30 days | | | |  |  |  |  |  |  |
|  | Influenza | 6/483 (1·2%) | 21/6,883 (0·3%) | 4·11 (1·65-10·23) | 0·002* | 3·48 (1·28-9·43) | 0·014* | 3·17 (1·15-8·73) | 0·025* |
|  | URI | 102/483 (21·1%) | 1,459/6,883 (21·2%) | 1·00 (0·79-1·25) | 0·967 | 1·41 (1·10-1·81) | 0·007* | 1·39 (1·08-1·78) | 0·01* |
| Previous 1-90 days | | | |  |  |  |  |  |  |
|  | Influenza | 12/483 (2·5%) | 121/6,883 (1·8%) | 1·43 (0·78-2·60) | 0·247 | 1·55 (0·81-2·98) | 0·185 | 1·50 (0·78-2·89) | 0·222 |
|  | URI | 147/483 (30·4%) | 2,251/6,883 (32·7%) | 0·90 (0·74-1·10) | 0·304 | 1·23 (0·99-1·54) | 0·062 | 1·23 (0·98-1·53) | 0·072 |
| **Asthma (n = 704)** | | | |  |  |  |  |  |  |
| Previous 1-14 days | | | |  |  |  |  |  |  |
|  | Influenza | 1/86 (1·2%) | 2/618 (0·3%) | 3·62 (0·33-40·39) | 0·295 | 10·77 (0·70-165·17) | 0·088 | 10·21 (0·66-157·83) | 0·096 |
|  | URI | 17/86 (19·8%) | 126/618 (20·4%) | 0·96 (0·55-1·69) | 0·894 | 1·16 (0·62-2·17) | 0·648 | 1·12 (0·59-2·10) | 0·733 |
| Previous 1-30 days | | | |  |  |  |  |  |  |
|  | Influenza | 1/86 (1·2%) | 4/618 (0·6%) | 1·81 (0·20-16·35) | 0·599 | 8·57 (0·62-117·90) | 0·108 | 9·22 (0·66-128·91) | 0·099 |
|  | URI | 17/86 (19·8%) | 163/618 (26·4%) | 0·69 (0·39-1·20) | 0·191 | 0·82 (0·45-1·52) | 0·537 | 0·80 (0·43-1·49) | 0·489 |
| Previous 1-90 days | | | |  |  |  |  |  |  |
|  | Influenza | 2/86 (2·3%) | 11/618 (1·8%) | 1·31 (0·29-6·03) | 0·725 | 1·52 (0·27-8·69) | 0·640 | 1·52 (0·26-8·80) | 0·639 |
|  | URI | 30/86 (34·9%) | 266/618 (43·0%) | 0·71 (0·44-1·14) | 0·153 | 0·89 (0·52-1·52) | 0·673 | 0·89 (0·52-1·52) | 0·671 |
| **Non-COPD (n = 7,806)** | | | |  |  |  |  |  |  |
| Previous 1-14 days | | | |  |  |  |  |  |  |
|  | Influenza | 6/514 (1·2%) | 20/7,292 (0·3%) | 4·33 (2·02-10·94) | <0·001* | 4·87 (1·88-12·65) | 0·001* | 4·50 (1·71-11·88) | 0·002* |
|  | URI | 101/514 (19·6%) | 1,296/7,292 (17·8%) | 1·13 (0·90-1·42) | 0·284 | 1·42 (1·11-1·82) | 0·005* | 1·39 (1·09-1·78) | 0·008* |
| Previous 1-30 days | | | |  |  |  |  |  |  |
|  | Influenza | 7/514 (1·4%) | 24/7,292 (0·3%) | 4·18 (1·79-9·75) | 0·001* | 3·90 (1·55-9·81) | 0·004* | 3·63 (1·43-9·24) | 0·007* |
|  | URI | 112/514 (21·8%) | 1,575/7,292 (21·6%) | 1·01 (0·81-1·26) | 0·919 | 1·33 (1·05-1·68) | 0·019* | 1·30 (1·03-1·65) | 0·029* |
| Previous 1-90 days | | | |  |  |  |  |  |  |
|  | Influenza | 13/514 (2·5%) | 124/7,292 (1·7%) | 1·50 (0·84-2·68) | 0·170 | 1·73 (0·93-3·23) | 0·084 | 1·69 (0·90-3·15) | 0·101 |
|  | URI | 165/514 (32·1%) | 2,440/7,292 (33·5%) | 0·94 (0·78-1·14) | 0·527 | 1·20 (0·97-1·48) | 0·096 | 1·19 (0·96-1·47) | 0·113 |
| **COPD (n = 264)** | | | |  |  |  |  |  |  |
| Previous 1-14 days | | | |  |  |  |  |  |  |
|  | Influenza | 0/55 (0·0%) | 1/209 (0·5%) | N/A |  | N/A |  | N/A |  |
|  | URI | 7/55 (12·7%) | 37/209 (17·7%) | 0·79 (0·35-1·81) | 0·580 | 1·49 (0·58-3·86) | 0·412 | 1·49 (0·57-3·85) | 0·414 |
| Previous 1-30 days | | | |  |  |  |  |  |  |
|  | Influenza | 0/55 (0·0%) | 1/209 (0·5%) | N/A |  | N/A |  | N/A |  |
|  | URI | 7/55 (12·7%) | 47/209 (22·5%) | 0·50 (0·21-1·18) | 0·116 | 0·87 (0·34-2·26) | 0·775 | 0·87 (0·34-2·25) | 0·774 |
| Previous 1-90 days | | | |  |  |  |  |  |  |
|  | Influenza | 1/55 (1·8%) | 8/209 (3·8%) | 0·47 (0·06-3·80) | 0·475 | 0·78 (0·08-7·88) | 0·834 | 0·76 (0·08-7·71) | 0·816 |
|  | URI | 12/55 (21·8%) | 77/209 (36·8%) | 0·48 (0·24-0·96) | 0·039* | 0·83 (0·37-1·87) | 0·659 | 0·83 (0·37-1·86) | 0·651 |
| **Non-hypertension (n = 6,413)** | | | |  |  |  |  |  |  |
| Previous 1-14 days | | | |  |  |  |  |  |  |
|  | Influenza | 4/294 (1·4%) | 19/6,119 (0·3%) | 4·39 (1·86-12·98) | 0·001* | 3·53 (1·16-10·74) | 0·027* | 3·16 (1·01-9·84) | 0·047* |
|  | URI | 66/294 (22·4%) | 1,089/6,119 (17·8%) | 1·34 (1·01-1·77) | 0·043* | 1·56 (1·15-2·11) | 0·004* | 1·53 (1·13-2·08) | 0·006* |
| Previous 1-30 days | | | |  |  |  |  |  |  |
|  | Influenza | 5/294 (1·7%) | 22/6,119 (0·4%) | 4·80 (1·80-12·75) | 0·002* | 2·99 (1·00-8·87) | 0·049* | 2·75 (0·92-8·29) | 0·071 |
|  | URI | 70/294 (23·8%) | 1,346/6,119 (22·0%) | 1·11 (0·84-1·46) | 0·464 | 1·32 (0·98-1·77) | 0·067 | 1·29 (0·96-1·74) | 0·090 |
| Previous 1-90 days | | | |  |  |  |  |  |  |
|  | Influenza | 6/294 (2·0%) | 115/6,119 (1·9%) | 1·09 (0·48-2·49) | 0·843 | 0·95 (0·39-2·27) | 0·901 | 0·90 (0·37-2·17) | 0·815 |
|  | URI | 106/294 (36·1%) | 2,077/6,119 (33·9%) | 1·10 (0·86-1·40) | 0·456 | 1·33 (1·02-1·73) | 0·037* | 1·33 (1·02-1·73) | 0·036* |
| **Hypertension (n = 1,657)** | | | |  |  |  |  |  |  |
| Previous 1-14 days | | | |  |  |  |  |  |  |
|  | Influenza | 2/275 (0·7%) | 2/1,382 (0·1%) | 5·06 (0·71-36·04) | 0·106 | 11·62 (1·53-88·36) | 0·018* | 11·67 (1·51-90·53) | 0·019* |
|  | URI | 42/275 (15·3%) | 244/1,382 (17·7%) | 0·86 (0·61-1·23) | 0·419 | 1·24 (0·84-1·82) | 0·277 | 1·23 (0·84-1·81) | 0·289 |
| Previous 1-30 days | | | |  |  |  |  |  |  |
|  | Influenza | 2/275 (0·7%) | 3/1,382 (0·2%) | 3·37 (0·56-20·25) | 0·185 | 7·29 (1·14-46·42) | 0·036* | 7·36 (1·14-47·74) | 0·036* |
|  | URI | 49/275 (17·8%) | 276/1,382 (20·0%) | 0·87 (0·62-1·22) | 0·412 | 1·26 (0·87-1·82) | 0·220 | 1·26 (0·87-1·82) | 0·222 |
| Previous 1-90 days | | | |  |  |  |  |  |  |
|  | Influenza | 8/275 (2·9%) | 17/1,382 (1·2%) | 2·41 (1·03-5·63) | 0·043* | 3·57 (1·37-9·32) | 0·009* | 3·57 (1·37-9·32) | 0·009* |
|  | URI | 71/275 (25·8%) | 440/1,382 (31·8%) | 0·75 (0·56-1·00) | 0·049* | 0·97 (0·71-1·34) | 0·873 | 0·98 (0·71-1·35) | 0·879 |

Abbreviations: COPD, Chronic obstructive pulmonary disease; Upper respiratory tract infection, URI; COVID-19, Coronavirus Disease 2019; N/A, Not applicable; SD, Standard deviation

* Unconditional logistic regression model, Significance at P < 0·05

† Model 1 was adjusted for age, sex, income, CCI scores, asthma, COPD, and hypertension

‡ Model 2 was adjusted for model 1 plus influenza and URI
