# Supplementary material for: The associations of previous influenza/upper respiratory infection with COVID-19 susceptibility/morbidity/mortality: a nationwide cohort study in South Korea
Source: Sci Rep. 2021 Nov 3;11:21568. doi: 10.1038/s41598-021-00428-x (PMC8566493; doi:10.1038/s41598-021-00428-x)
Supplement: Supplementary file 6 — Supplementary Information 6. [file 41598_2021_428_MOESM6_ESM.docx]

**Table S6** Crude and adjusted odds ratios of influenza and URI (previous 15-45, 15-90, 31-90, 1-365 days) for morbidity in COVID-19 participants

| Characteristics | | Severe participant | Mild participants | ORs (95% confidence interval) for morbidity | | | | | |
| --- | --- | --- | --- | --- | --- | --- | --- | --- | --- |
|  |  | (exposure/total, %) | (exposure/total, %) | Crude | P-value | Model 1† | P-value | Model 2†‡ | P-value |
| **Previous 15-45 days** | | | |  |  |  |  |  |  |
|  | Influenza | 2/569 (0·4%) | 33/7,501 (0·4%) | 0·80 (0·19-3·34) | 0·758 | 0·72 (0·15-3·47) | 0·684 | 0·72 (0·15-3·49) | 0·688 |
|  | URI | 51/569 (9·0%) | 865/7,501 (11·5%) | 0·76 (0·56-1·02) | 0·064 | 0·92 (0·67-1·27) | 0·628 | 0·93 (0·67-1·27) | 0·631 |
| **Previous 15-90 days** | | | |  |  |  |  |  |  |
|  | Influenza | 7/569 (1·2%) | 113/7,501 (1·5%) | 0·81 (0·38-1·76) | 0·601 | 0·88 (0·39-2·01) | 0·760 | 0·88 (0·38-2·00) | 0·753 |
|  | URI | 113/569 (19·9%) | 1,692/7,501 (22·6%) | 0·85 (0·69-1·05) | 0·137 | 1·06 (0·84-1·34) | 0·632 | 1·06 (0·84-1·34) | 0·627 |
| **Previous 31-90 days** | | | |  |  |  |  |  |  |
|  | Influenza | 6/569 (1·1%) | 106/7,501 (1·4%) | 0·74 (0·33-1·70) | 0·483 | 0·80 (0·33-1·94) | 0·614 | 0·79 (0·33-1·93) | 0·610 |
|  | URI | 94/569 (16·5%) | 1,378/7,501 (18·4%) | 0·88 (0·70-1·11) | 0·271 | 1·04 (0·81-1·33) | 0·774 | 1·04 (0·81-1·34) | 0·764 |
| **The number of medical visit previous 1-365 days (Days, mean, SD)** | | | |  |  |  |  |  |  |
|  | Influenza | 0·040 (0·27) | 0·027 (0·18) | 1·36 (0·94-1·96) | 0·107 | 1·49 (0·99-2·25) | 0·058 | 1·48 (0·98-2·24) | 0·061 |
|  | URI | 1·605 (4·19) | 1·543 (3·03) | 1·01 (0·98-1·03) | 0·650 | 1·02 (0·99-1·04) | 0·222 | 1·02 (0·99-1·04) | 0·233 |

* Unconditional logistic regression model, Significance at P < 0·05

† Model 1 was adjusted for age, sex, income, CCI scores, asthma, COPD, and hypertension

‡ Model 2 was adjusted for model 1 plus influenza and URI
