# Supplementary material for: The associations of previous influenza/upper respiratory infection with COVID-19 susceptibility/morbidity/mortality: a nationwide cohort study in South Korea
Source: Sci Rep. 2021 Nov 3;11:21568. doi: 10.1038/s41598-021-00428-x (PMC8566493; doi:10.1038/s41598-021-00428-x)
Supplement: Supplementary file 7 — Supplementary Information 7. [file 41598_2021_428_MOESM7_ESM.docx]

**Table S7** Subgroup analyses of crude and adjusted odds ratios of influenza and URI (previous 15-45, 15-90, 31-90, and 1-365 days) for morbidity in COVID-19 participants by covariates

| Characteristics | | Severe participants | Mild participants | ORs (95% confidence interval) for morbidity | | | | | |
| --- | --- | --- | --- | --- | --- | --- | --- | --- | --- |
|  |  | (exposure/total, %) | (exposure/total, %) | Crude | P-value | Model 1† | P-value | Model 2†‡ | P-value |
| **Age < 50 years old ( n = 4,282)** | | | |  |  |  |  |  |  |
| Previous 15-45 days | | | |  |  |  |  |  |  |
|  | Influenza | 0/98 (0·0%) | 20/4,184 (0·5%) | N/A |  | N/A |  | N/A |  |
|  | URI | 10/98 (10·2%) | 493/4,184 (11·8%) | 0·85 (0·44-1·65) | 0·632 | 0·82 (0·42-1·60) | 0·555 | 0·82 (0·42-1·61) | 0·566 |
| Previous 15-90 days | | | |  |  |  |  |  |  |
|  | Influenza | 0/98 (0·0%) | 67/4,184 (1·6%) | N/A |  | N/A |  | N/A |  |
|  | URI | 25/98 (25·5%) | 988/4,184 (23·6%) | 1·11 (0·70-1·75) | 0·663 | 1·02 (0·64-1·64) | 0·924 | 1·04 (0·65-1·67) | 0·876 |
| Previous 31-90 days | | | |  |  |  |  |  |  |
|  | Influenza | 0/98 (0·0%) | 63/4,184 (1·5%) | N/A |  | N/A |  | N/A |  |
|  | URI | 22/98 (22·4%) | 807/4,184 (19·3%) | 1·21 (0·75-1·96) | 0·434 | 1·08 (0·66-1·78) | 0·755 | 1·11 (0·67-1·81) | 0·694 |
| The number of medical visits previous 1-365 days (Days, mean, SD) | | | | | |  |  |  |  |
|  | Influenza | 0·041 (0·40) | 0·034 (0·20) | 1·16 (0·49-2·74) | 0·737 | 0·91 (0·41-2·06) | 0·825 | 0·92 (0·41-2·06) | 0·836 |
|  | URI | 1·663 (3·23) | 1·602 (2·89) | 1·01 (0·94-1·08) | 0·836 | 0·98 (0·91-1·05) | 0·505 | 0·98 (0·91-1·05) | 0·507 |
| **Age ≥ 50 years old (n = 3,788)** | | | |  |  |  |  |  |  |
| Previous 15-45 days | | | |  |  |  |  |  |  |
|  | Influenza | 2/471 (0·4%) | 13/3,317 (0·4%) | 1·09 (0·25-4·82) | 0·913 | 0·96 (0·19-4·90) | 0·959 | 0·96 (0·19-4·91) | 0·960 |
|  | URI | 41/471 (8·7%) | 372/3,317 (11·2%) | 0·76 (0·54-1·06) | 0·103 | 0·96 (0·67-1·38) | 0·824 | 0·96 (0·67-1·38) | 0·824 |
| Previous 15-90 days | | | |  |  |  |  |  |  |
|  | Influenza | 7/471 (1·5%) | 46/3,317 (1·4%) | 1·07 (0·48-2·39) | 0·864 | 1·23 (0·52-2·94) | 0·642 | 1·23 (0·51-2·93) | 0·645 |
|  | URI | 88/471 (18·7%) | 704/3,317 (21·2%) | 0·85 (0·67-1·09) | 0·205 | 1·06 (0·81-1·38) | 0·690 | 1·06 (0·81-1·38) | 0·693 |
| Previous 31-90 days | | | |  |  |  |  |  |  |
|  | Influenza | 6/471 (1·3%) | 43/3,317 (1·3%) | 0·98 (0·42-2·32) | 0·968 | 1·10 (0·43-2·81) | 0·843 | 1·10 (0·43-2·81) | 0·843 |
|  | URI | 72/471 (15·3%) | 571/3,317 (17·2%) | 0·87 (0·67-1·13) | 0·297 | 1·00 (0·75-1·34) | 0·991 | 1·00 (0·75-1·34) | 0·992 |
| The number of medical visits previous 1-365 days (Days, mean, SD) | | | | | |  |  |  |  |
|  | Influenza | 0·040 (0·23) | 0·019 (0·15) | 1·80 (1·15-2·82) | 0·01* | 1·84 (1·10-3·07) | 0·02* | 1·83 (1·10-3·06) | 0·021* |
|  | URI | 1·592 (4·37) | 1·468 (3·19) | 1·01 (0·98-1·04) | 0·455 | 1·02 (0·99-1·05) | 0·130 | 1·02 (0·99-1·05) | 0·133 |
| **Men (n = 3,236)** | | | |  |  |  |  |  |  |
| Previous 15-45 days | | | |  |  |  |  |  |  |
|  | Influenza | 1/306 (0·3%) | 6/2,930 (0·2%) | 1·60 (0·19-13·33) | 0·662 | 1·78 (0·19-16·95) | 0·618 | 1·80 (0·19-17·37) | 0·610 |
|  | URI | 26/306 (8·5%) | 281/2,930 (9·6%) | 0·88 (0·58-1·33) | 0·535 | 0·94 (0·59-1·49) | 0·784 | 0·93 (0·59-1·48) | 0·770 |
| Previous 15-90 days | | | |  |  |  |  |  |  |
|  | Influenza | 4/306 (1·3%) | 46/2,930 (1·6%) | 0·83 (0·30-2·32) | 0·723 | 1·05 (0·35-3·12) | 0·936 | 1·04 (0·35-3·11) | 0·938 |
|  | URI | 57/306 (18·6%) | 565/2,930 (19·3%) | 0·96 (0·71-1·30) | 0·782 | 1·04 (0·74-1·45) | 0·831 | 1·04 (0·74-1·45) | 0·832 |
| Previous 31-90 days | | | |  |  |  |  |  |  |
|  | Infected | 3/306 (1·0%) | 45/2,930 (1·5%) | 0·64 (0·20-2·06) | 0·448 | 0·77 (0·23-2·66) | 0·682 | 0·77 (0·23-2·66) | 0·682 |
|  | Infected | 47/306 (15·4%) | 465/2,930 (15·9%) | 0·96 (0·70-1·33) | 0·818 | 0·99 (0·69-1·42) | 0·959 | 0·99 (0·69-1·43) | 0·961 |
| The number of medical visits previous 1-365 days (Days, mean, SD) | | | | | |  |  |  |  |
|  | Influenza | 0·039 (0·24) | 0·028 (0·19) | 1·29 (0·77-2·15) | 0·341 | 1·56 (0·86-2·86) | 0·146 | 1·55 (0·85-2·82) | 0·156 |
|  | URI | 1·618 (3·74) | 1·274 (2·60) | 1·04 (1·00-1·07) | 0·04* | 1·03 (1·00-1·07) | 0·072 | 1·03 (1·00-1·07) | 0·076 |
| **Women (n = 4,834)** | | | |  |  |  |  |  |  |
| Previous 15-45 days | | | |  |  |  |  |  |  |
|  | Influenza | 1/263 (0·4%) | 27/4,571 (0·6%) | 0·64 (0·09-4·75) | 0·666 | 0·40 (0·05-3·39) | 0·397 | 0·40 (0·05-3·40) | 0·397 |
|  | URI | 25/263 (9·5%) | 584/4,571 (12·8%) | 0·72 (0·47-1·09) | 0·122 | 0·90 (0·57-1·41) | 0·639 | 0·90 (0·57-1·41) | 0·640 |
| Previous 15-90 days | | | |  |  |  |  |  |  |
|  | Influenza | 3/263 (1·1%) | 67/4,571 (1·5%) | 0·78 (0·24-2·48) | 0·669 | 0·73 (0·20-2·64) | 0·635 | 0·73 (0·20-2·62) | 0·626 |
|  | URI | 56/263 (21·3%) | 1,127/4,571 (24·7%) | 0·83 (0·61-1·12) | 0·218 | 1·08 (0·78-1·49) | 0·655 | 1·08 (0·78-1·50) | 0·645 |
| Previous 31-90 days | | | |  |  |  |  |  |  |
|  | Influenza | 3/263 (1·1%) | 61/4,571 (1·3%) | 0·85 (0·27-2·74) | 0·789 | 0·87 (0·24-3·20) | 0·830 | 0·86 (0·23-3·17) | 0·815 |
|  | URI | 47/263 (17·9%) | 913/4,571 (20·0%) | 0·87 (0·63-1·21) | 0·406 | 1·08 (0·76-1·53) | 0·660 | 1·08 (0·76-1·54) | 0·652 |
| The number of medical visits previous 1-365 days (Days, mean, SD) | | | | | |  |  |  |  |
|  | Influenza | 0·042 (0·29) | 0·026 (0·18) | 1·42 (0·84-2·41) | 0·194 | 1·49 (0·85-2·62) | 0·166 | 1·49 (0·85-2·62) | 0·165 |
|  | URI | 1·589 (4·68) | 1·715 (3·26) | 0·99 (0·95-1·03) | 0·553 | 1·00 (0·96-1·04) | 0·923 | 1·00 (0·96-1·04) | 0·918 |
| **Low income (n = 2,836)** | | | |  |  |  |  |  |  |
| Previous 15-45 days | | | |  |  |  |  |  |  |
|  | Influenza | 1/185 (0·5%) | 12/2,651 (0·5%) | 1·20 (0·16-9·24) | 0·864 | 0·66 (0·07-6·28) | 0·714 | 0·65 (0·07-6·26) | 0·711 |
|  | URI | 13/185 (7·0%) | 317/2,651 (12·0%) | 0·56 (0·31-0·99) | 0·046* | 0·90 (0·49-1·65) | 0·732 | 0·90 (0·49-1·65) | 0·728 |
| Previous 15-90 days | | | |  |  |  |  |  |  |
|  | Influenza | 5/185 (2·7%) | 52/2,651 (2·0%) | 1·39 (0·55-3·52) | 0·488 | 1·28 (0·47-3·45) | 0·628 | 1·28 (0·47-3·46) | 0·627 |
|  | URI | 30/185 (16·2%) | 605/2,651 (22·8%) | 0·66 (0·44-0·98) | 0·039* | 1·02 (0·66-1·57) | 0·940 | 1·02 (0·66-1·58) | 0·933 |
| Previous 31-90 days | | | |  |  |  |  |  |  |
|  | Influenza | 4/185 (2·2%) | 49/2,651 (1·8%) | 1·17 (0·42-3·29) | 0·761 | 1·09 (0·36-3·27) | 0·880 | 1·09 (0·36-3·27) | 0·879 |
|  | URI | 25/185 (13·5%) | 482/2,651 (18·2%) | 0·70 (0·46-1·08) | 0·111 | 1·05 (0·66-1·68) | 0·839 | 1·05 (0·66-1·68) | 0·839 |
| The number of medical visits previous 1-365 days (Days, mean, SD) | | | | | |  |  |  |  |
|  | Influenza | 0·049 (0·26) | 0·033 (0·21) | 1·33 (0·74-2·39) | 0·338 | 1·45 (0·76-2·76) | 0·258 | 1·45 (0·76-2·76) | 0·257 |
|  | URI | 1·281 (2·65) | 1·564 (3·48) | 0·97 (0·91-1·03) | 0·266 | 1·00 (0·96-1·05) | 0·939 | 1·00 (0·96-1·05) | 0·914 |
| **Middle income (n = 3,325)** | | | |  |  |  |  |  |  |
| Previous 15-45 days | | | |  |  |  |  |  |  |
|  | Influenza | 0/211 (0·0%) | 16/3,114 (0·5%) | N/A |  | N/A |  | N/A |  |
|  | URI | 22/211 (10·4%) | 354/3,114 (11·4%) | 0·91 (0·58-1·43) | 0·676 | 0·98 (0·60-1·61) | 0·949 | 0·99 (0·60-1·62) | 0·969 |
| Previous 15-90 days | | | |  |  |  |  |  |  |
|  | Influenza | 0/211 (0·0%) | 40/3,114 (1·3%) | N/A |  | N/A |  | N/A |  |
|  | URI | 47/211 (22·3%) | 695/3,114 (22·3%) | 1·00 (0·71-1·40) | 0·988 | 1·22 (0·85-1·76) | 0·288 | 1·23 (0·86-1·78) | 0·262 |
| Previous 31-90 days | | | |  |  |  |  |  |  |
|  | Influenza | 0/211 (0·0%) | 38/3,114 (1·2%) | N/A |  | N/A |  | N/A |  |
|  | URI | 37/211 (17·5%) | 576/3,114 (18·5%) | 0·94 (0·65-1·35) | 0·728 | 1·12 (0·76-1·67) | 0·567 | 1·13 (0·76-1·69) | 0·535 |
| The number of medical visits previous 1-365 days (Days, mean, SD) | | | | | |  |  |  |  |
|  | Influenza | 0·033 (0·30) | 0·024 (0·16) | 1·28 (0·66-2·50) | 0·468 | 1·49 (0·74-3·01) | 0·267 | 1·50 (0·74-3·02) | 0·262 |
|  | URI | 2·009 (5·73) | 1·554 (2·92) | 1·03 (1·00-1·07) | 0·049* | 1·04 (1·00-1·07) | 0·068 | 1·04 (1·00-1·07) | 0·067 |
| **High income (n = 1,909)** | | | |  |  |  |  |  |  |
| Previous 15-45 days | | | |  |  |  |  |  |  |
|  | Influenza | 1/173 (0·6%) | 5/1,736 (0·3%) | 2·01 (0·23-17·33) | 0·524 | 1·25 (0·12-13·13) | 0·854 | 1·27 (0·12-13·50) | 0·842 |
|  | URI | 16/173 (9·2%) | 194/1,736 (11·2%) | 0·81 (0·47-1·38) | 0·441 | 0·90 (0·50-1·63) | 0·731 | 0·90 (0·50-1·62) | 0·726 |
| Previous 15-90 days | | | |  |  |  |  |  |  |
|  | Influenza | 2/173 (1·2%) | 21/1,736 (1·2%) | 0·96 (0·22-4·11) | 0·952 | 0·88 (0·18-4·37) | 0·872 | 0·88 (0·18-4·40) | 0·877 |
|  | URI | 36/173 (20·8%) | 392/1,736 (22·6%) | 0·90 (0·61-1·32) | 0·594 | 0·98 (0·64-1·50) | 0·914 | 0·98 (0·64-1·50) | 0·921 |
| Previous 31-90 days | | | |  |  |  |  |  |  |
|  | Influenza | 2/173 (1·2%) | 19/1,736 (1·1%) | 1·06 (0·25-4·58) | 0·940 | 1·01 (0·20-5·19) | 0·994 | 1·01 (0·20-5·19) | 0·995 |
|  | URI | 32/173 (18·5%) | 320/1,736 (18·4%) | 1·00 (0·67-1·50) | 0·984 | 1·01 (0·64-1·57) | 0·982 | 1·01 (0·64-1·58) | 0·982 |
| The number of medical visits previous 1-365 days (Days, mean, SD) | | | | | |  |  |  |  |
|  | Influenza | 0·040 (0·23) | 0·023 (0·17) | 1·54 (0·77-3·10) | 0·225 | 1·47 (0·63-3·44) | 0·377 | 1·48 (0·63-3·49) | 0·366 |
|  | URI | 1·457 (3·19) | 1·491 (2·41) | 0·99 (0·93-1·06) | 0·861 | 0·99 (0·93-1·06) | 0·797 | 0·99 (0·93-1·05) | 0·752 |
| **CCI scores = 0 (n = 6,518)** | | | |  |  |  |  |  |  |
| Previous 15-45 days | | | |  |  |  |  |  |  |
|  | Influenza | 1/264 (0·4%) | 26/6,254 (0·4%) | 0·91 (0·12-6·74) | 0·927 | 0·91 (0·12-7·17) | 0·927 | 0·94 (0·12-7·53) | 0·957 |
|  | URI | 27/264 (10·2%) | 763/6,254 (12·2%) | 0·82 (0·55-1·23) | 0·337 | 0·75 (0·49-1·16) | 0·195 | 0·75 (0·49-1·16) | 0·195 |
| Previous 15-90 days | | | |  |  |  |  |  |  |
|  | Influenza | 2/264 (0·8%) | 90/6,254 (1·4%) | 0·52 (0·13-2·13) | 0·366 | 0·55 (0·13-2·30) | 0·413 | 0·55 (0·13-2·30) | 0·413 |
|  | URI | 65/264 (24·6%) | 1,473/6,254 (23·6%) | 1·06 (0·80-1·41) | 0·689 | 0·99 (0·73-1·35) | 0·962 | 1·00 (0·74-1·35) | 0·989 |
| Previous 31-90 days | | | |  |  |  |  |  |  |
|  | Influenza | 1/264 (0·4%) | 85/6,254 (1·4%) | 0·28 (0·04-1·99) | 0·201 | 0·29 (0·04-2·15) | 0·227 | 0·29 (0·04-2·15) | 0·227 |
|  | URI | 53/264 (20·1%) | 1,189/6,254 (19·0%) | 1·07 (0·79-1·46) | 0·666 | 0·98 (0·71-1·36) | 0·916 | 0·99 (0·71-1·38) | 0·957 |
| The number of medical visits previous 1-365 days (Days, mean, SD) | | | | | |  |  |  |  |
|  | Influenza | 0·023 (0·17) | 0·028 (0·19) | 0·85 (0·41-1·79) | 0·673 | 0·94 (0·44-2·00) | 0·864 | 0·93 (0·44-2·00) | 0·857 |
|  | URI | 1·795 (2·93) | 1·581 (3·02) | 1·02 (0·99-1·05) | 0·257 | 1·00 (0·97-1·04) | 0·864 | 1·00 (0·97-1·04) | 0·857 |
| **CCI scores = 1 (n = 889)** | | | |  |  |  |  |  |  |
| Previous 15-45 days | | | |  |  |  |  |  |  |
|  | Influenza | 1/134 (0·7%) | 3/755 (0·4%) | 1·89 (0·20-18·26) | 0·584 | 2·26 (0·18-27·79) | 0·526 | 2·31 (0·19-28·50) | 0·514 |
|  | URI | 14/134 (10·4%) | 74/755 (9·8%) | 1·07 (0·59-1·96) | 0·817 | 1·27 (0·66-2·44) | 0·468 | 1·28 (0·67-2·46) | 0·459 |
| Previous 15-90 days | | | |  |  |  |  |  |  |
|  | Influenza | 3/134 (2·2%) | 14/755 (1·9%) | 1·21 (0·34-4·28) | 0·765 | 2·09 (0·52-8·38) | 0·299 | 2·11 (0·52-8·46) | 0·295 |
|  | URI | 23/134 (17·2%) | 148/755 (19·6%) | 0·85 (0·52-1·38) | 0·510 | 1·04 (0·61-1·77) | 0·893 | 1·05 (0·62-1·80) | 0·851 |
| Previous 31-90 days | | | |  |  |  |  |  |  |
|  | Influenza | 3/134 (2·2%) | 14/755 (1·9%) | 1·21 (0·34-4·28) | 0·765 | 2·09 (0·52-8·38) | 0·299 | 2·05 (0·51-8·22) | 0·314 |
|  | URI | 18/134 (13·4%) | 131/755 (17·4%) | 0·74 (0·44-1·26) | 0·265 | 0·85 (0·47-1·51) | 0·570 | 0·86 (0·48-1·54) | 0·603 |
| The number of medical visits previous 1-365 days (Days, mean, SD) | | | | | |  |  |  |  |
|  | Influenza | 0·030 (0·17) | 0·024 (0·16) | 1·23 (0·43-3·51) | 0·694 | 1·70 (0·55-5·22) | 0·357 | 1·73 (0·56-5·32) | 0·342 |
|  | URI | 2·172 (7·21) | 1·522 (3·33) | 1·03 (0·99-1·07) | 0·113 | 1·03 (0·99-1·07) | 0·099 | 1·03 (0·99-1·07) | 0·096 |
| **CCI scores ≥ 2 (n = 663)** | | | |  |  |  |  |  |  |
| Previous 15-45 days | | | |  |  |  |  |  |  |
|  | Influenza | 0/171 (0·0%) | 4/492 (0·8%) | N/A |  | N/A |  | N/A |  |
|  | URI | 10/171 (5·8%) | 28/492 (5·7%) | 1·03 (0·49-2·17) | 0·939 | 1·61 (0·69-3·74) | 0·267 | 1·60 (0·69-3·71) | 0·276 |
| Previous 15-90 days | | | |  |  |  |  |  |  |
|  | Influenza | 2/171 (1·2%) | 9/492 (1·8%) | 0·64 (0·14-2·97) | 0·565 | 0·73 (0·14-3·73) | 0·704 | 0·71 (0·14-3·72) | 0·686 |
|  | URI | 25/171 (14·6%) | 71/492 (14·4%) | 1·02 (0·62-1·66) | 0·952 | 1·58 (0·91-2·73) | 0·105 | 1·58 (0·91-2·73) | 0·104 |
| Previous 31-90 days | | | |  |  |  |  |  |  |
|  | Influenza | 2/171 (1·2%) | 7/492 (1·4%) | 0·82 (0·17-3·99) | 0·806 | 0·83 (0·16-4·46) | 0·831 | 0·79 (0·14-4·38) | 0·791 |
|  | URI | 23/171 (13·5%) | 58/492 (11·8%) | 1·16 (0·69-1·95) | 0·568 | 1·75 (0·99-3·11) | 0·056 | 1·75 (0·99-3·11) | 0·055 |
| The number of medical visits previous 1-365 days (Days, mean, SD) | | | | | |  |  |  |  |
|  | Influenza | 0·076 (0·41) | 0·024 (0·15) | 2·13 (1·04-4·34) | 0·038* | 2·37 (1·10-5·08) | 0·027* | 2·37 (1·11-5·10) | 0·027* |
|  | URI | 0·865 (1·98) | 1·093 (2·62) | 0·96 (0·89-1·04) | 0·303 | 1·01 (0·93-1·10) | 0·853 | 1·01 (0·93-1·10) | 0·791 |
| **Non-asthma (n =7,366 )** | | | |  |  |  |  |  |  |
| Previous 15-45 days | | | |  |  |  |  |  |  |
|  | Influenza | 1/483 (0·2%) | 27/6,883 (0·4%) | 0·53 (0·07-3·89) | 0·530 | 0·86 (0·11-6·56) | 0·887 | 0·86 (0·11-6·56) | 0·886 |
|  | URI | 42/483 (8·7%) | 761/6,883 (11·1%) | 0·77 (0·55-1·06) | 0·109 | 1·01 (0·71-1·43) | 0·969 | 1·01 (0·71-1·43) | 0·965 |
| Previous 15-90 days | | | |  |  |  |  |  |  |
|  | Influenza | 6/483 (1·2%) | 104/6,883 (1·5%) | 0·82 (0·36-1·88) | 0·639 | 0·92 (0·38-2·20) | 0·843 | 0·90 (0·37-2·17) | 0·809 |
|  | URI | 95/483 (19·7%) | 1,489/6,883 (21·6%) | 0·89 (0·70-1·12) | 0·310 | 1·17 (0·91-1·51) | 0·214 | 1·18 (0·91-1·51) | 0·211 |
| Previous 31-90 days | | | |  |  |  |  |  |  |
|  | Influenza | 5/483 (1·0%) | 98/6,883 (1·4%) | 0·73 (0·29-1·79) | 0·485 | 0·82 (0·32-2·14) | 0·686 | 0·81 (0·31-2·10) | 0·658 |
|  | URI | 78/483 (16·1%) | 1,205/6,883 (17·5%) | 0·91 (0·71-1·17) | 0·447 | 1·15 (0·87-1·50) | 0·328 | 1·15 (0·88-1·51) | 0·319 |
| The number of medical visits previous 1-365 days (Days, mean, SD) | | | | | |  |  |  |  |
|  | Influenza | 0·037 (0·22) | 0·026 (0·18) | 1·35 (0·87-2·08) | 0·178 | 1·38 (0·84-2·26) | 0·206 | 1·37 (0·83-2·24) | 0·217 |
|  | URI | 1·329 (2·84) | 1·429 (2·81) | 0·99 (0·95-1·02) | 0·452 | 1·01 (0·98-1·05) | 0·498 | 1·01 (0·98-1·04) | 0·536 |
| **Asthma (n = 704)** | | | |  |  |  |  |  |  |
| Previous 15-45 days | | | |  |  |  |  |  |  |
|  | Influenza | 1/86 (1·2%) | 6/618 (1·0%) | 1·20 (0·14-10·09) | 0·867 | 0·75 (0·07-7·75) | 0·807 | 0·71 (0·07-7·34) | 0·772 |
|  | URI | 9/86 (10·5%) | 104/618 (16·8%) | 0·58 (0·28-1·19) | 0·136 | 0·67 (0·31-1·46) | 0·311 | 0·67 (0·31-1·45) | 0·306 |
| Previous 15-90 days | | | |  |  |  |  |  |  |
|  | Influenza | 1/86 (1·2%) | 9/618 (1·5%) | 0·80 (0·10-6·36) | 0·830 | 0·67 (0·07-6·54) | 0·728 | 0·62 (0·06-6·17) | 0·683 |
|  | URI | 18/86 (20·9%) | 203/618 (32·8%) | 0·54 (0·31-0·93) | 0·028* | 0·64 (0·35-1·18) | 0·156 | 0·64 (0·35-1·18) | 0·151 |
| Previous 31-90 days | | | |  |  |  |  |  |  |
|  | Influenza | 1/86 (1·2%) | 8/618 (1·3%) | 0·90 (0·11-7·26) | 0·919 | 0·67 (0·07-6·65) | 0·735 | 0·64 (0·06-6·37) | 0·701 |
|  | URI | 16/86 (18·6%) | 173/618 (28·0%) | 0·59 (0·33-1·04) | 0·068 | 0·65 (0·34-1·24) | 0·192 | 0·65 (0·34-1·23) | 0·188 |
| The number of medical visits previous 1-365 days (Days, mean, SD) | | | | | |  |  |  |  |
|  | Influenza | 0·058 (0·44) | 0·040 (0·24) | 1·22 (0·61-2·45) | 0·578 | 1·73 (0·81-3·68) | 0·156 | 1·73 (0·81-3·68) | 0·155 |
|  | URI | 3·151 (8·30) | 2·816 (4·61) | 1·01 (0·97-1·05) | 0·575 | 1·03 (0·98-1·07) | 0·236 | 1·03 (0·98-1·07) | 0·234 |
| **Non-COPD (n = 7,806)** | | | |  |  |  |  |  |  |
| Previous 15-45 days | | | |  |  |  |  |  |  |
|  | Influenza | 1/514 (0·2%) | 29/7,292 (0·4%) | 0·49 (0·07-3·59) | 0·481 | 0·76 (0·10-5·74) | 0·789 | 0·76 (0·10-5·75) | 0·790 |
|  | URI | 49/514 (9·5%) | 833/7,292 (11·4%) | 0·82 (0·60-1·11) | 0·192 | 0·99 (0·72-1·38) | 0·973 | 1·00 (0·72-1·38) | 0·979 |
| Previous 15-90 days | | | |  |  |  |  |  |  |
|  | Influenza | 6/514 (1·2%) | 106/7,292 (1·5%) | 0·80 (0·35-1·83) | 0·599 | 0·94 (0·39-2·25) | 0·889 | 0·93 (0·39-2·22) | 0·867 |
|  | URI | 107/514 (20·8%) | 1,633/7,292 (22·4%) | 0·91 (0·73-1·14) | 0·407 | 1·12 (0·88-1·43) | 0·346 | 1·12 (0·88-1·43) | 0·344 |
| Previous 31-90 days | | | |  |  |  |  |  |  |
|  | Influenza | 5/514 (1·0%) | 99/7,292 (1·4%) | 0·71 (0·29-1·76) | 0·465 | 0·85 (0·33-2·19) | 0·731 | 0·84 (0·32-2·18) | 0·717 |
|  | URI | 88/514 (17·1%) | 1,331/7,292 (18·3%) | 0·93 (0·73-1·17) | 0·520 | 1·07 (0·83-1·39) | 0·586 | 1·08 (0·83-1·39) | 0·578 |
| The number of medical visits previous 1-365 days (Days, mean, SD) | | | | | |  |  |  |  |
|  | Influenza | 0·041 (0·27) | 0·027 (0·18) | 1·37 (0·94-2·01) | 0·105 | 1·50 (0·99-2·29) | 0·056 | 1·50 (0·98-2·28) | 0·059 |
|  | URI | 1·671 (4·37) | 1·540 (3·04) | 1·01 (0·99-1·04) | 0·358 | 1·02 (0·99-1·04) | 0·163 | 1·02 (0·99-1·04) | 0·172 |
| **COPD (n = 264)** | | | |  |  |  |  |  |  |
| Previous 15-45 days | | | |  |  |  |  |  |  |
|  | Influenza | 1/55 (1·8%) | 4/209 (1·9%) | 0·95 (0·10-8·67) | 0·964 | 1·04 (0·09-12·08) | 0·977 | 0·95 (0·08-11·06) | 0·968 |
|  | URI | 2/55 (3·6%) | 32/209 (15·3%) | 0·21 (0·05-0·90) | 0·036* | 0·32 (0·07-1·47) | 0·142 | 0·32 (0·07-1·47) | 0·142 |
| Previous 15-90 days | | | |  |  |  |  |  |  |
|  | Influenza | 1/55 (1·8%) | 7/209 (3·3%) | 0·53 (0·06-4·44) | 0·562 | 0·80 (0·08-8·16) | 0·850 | 0·72 (0·07-7·48) | 0·783 |
|  | URI | 6/55 (10·9%) | 59/209 (28·2%) | 0·31 (0·13-0·77) | 0·011* | 0·42 (0·16-1·15) | 0·092 | 0·42 (0·16-1·14) | 0·090 |
| Previous 31-90 days | | | |  |  |  |  |  |  |
|  | Influenza | 1/55 (1·8%) | 7/209 (3·3%) | 0·53 (0·06-4·44) | 0·562 | 0·80 (0·08-8·16) | 0·850 | 0·76 (0·07-7·81) | 0·815 |
|  | URI | 6/55 (10·9%) | 47/209 (22·5%) | 0·42 (0·17-1·05) | 0·063 | 0·57 (0·20-1·58) | 0·278 | 0·56 (0·20-1·58) | 0·274 |
| The number of medical visits previous 1-365 days (Days, mean, SD) | | | | | |  |  |  |  |
|  | Influenza | 0·036 (0·19) | 0·038 (0·19) | 0·95 (0·20-4·60) | 0·948 | 1·48 (0·24-9·22) | 0·675 | 1·42 (0·22-8·95) | 0·712 |
|  | URI | 0·982 (1·68) | 1·660 (2·53) | 0·85 (0·72-1·01) | 0·065 | 0·92 (0·78-1·09) | 0·329 | 0·92 (0·78-1·09) | 0·338 |
| **Non-hypertension (n = 6,413)** | | | |  |  |  |  |  |  |
| Previous 15-45 days | | | |  |  |  |  |  |  |
|  | Influenza | 1/294 (0·3%) | 29/6,119 (0·5%) | 0·72 (0·10-5·28) | 0·744 | 0·79 (0·10-6·06) | 0·820 | 0·80 (0·10-6·14) | 0·829 |
|  | URI | 28/294 (9·5%) | 729/6,119 (11·9%) | 0·78 (0·52-1·16) | 0·216 | 0·89 (0·58-1·35) | 0·572 | 0·89 (0·58-1·35) | 0·575 |
| Previous 15-90 days | | | |  |  |  |  |  |  |
|  | Influenza | 1/294 (0·3%) | 98/6,119 (1·6%) | 0·21 (0·03-1·51) | 0·121 | 0·21 (0·03-1·51) | 0·120 | 0·20 (0·03-1·49) | 0·117 |
|  | URI | 65/294 (22·1%) | 1,406/6,119 (23·0%) | 0·95 (0·72-1·26) | 0·729 | 1·16 (0·86-1·57) | 0·336 | 1·17 (0·86-1·59) | 0·310 |
| Previous 31-90 days | | | |  |  |  |  |  |  |
|  | Influenza | 0/294 (0·0%) | 92/6,119 (1·5%) | N/A |  | N/A |  | N/A |  |
|  | URI | 52/294 (17·7%) | 1,142/6,119 (18·7%) | 0·94 (0·69-1·27) | 0·675 | 1·13 (0·81-1·57) | 0·475 | 1·14 (0·82-1·59) | 0·429 |
| The number of medical visits previous 1-365 days (Days, mean, SD) | | | | | |  |  |  |  |
|  | Influenza | 0·031 (0·27) | 0·030 (0·19) | 1·02 (0·57-1·84) | 0·951 | 1·01 (0·55-1·86) | 0·982 | 1·01 (0·54-1·86) | 0·987 |
|  | URI | 1·483 (3·41) | 1·560 (2·89) | 0·99 (0·95-1·03) | 0·657 | 1·00 (0·96-1·05) | 0·878 | 1·00 (0·96-1·05) | 0·879 |
| **Hypertension (n = 1,657)** | | | |  |  |  |  |  |  |
| Previous 15-45 days | | | |  |  |  |  |  |  |
|  | Influenza | 1/275 (0·4%) | 4/1,382 (0·3%) | 1·26 (0·14-11·29) | 0·838 | 0·69 (0·06-7·66) | 0·760 | 0·69 (0·06-7·66) | 0·760 |
|  | URI | 23/275 (8·4%) | 136/1,382 (9·8%) | 0·84 (0·53-1·33) | 0·448 | 1·00 (0·61-1·65) | 0·993 | 1·00 (0·61-1·65) | 0·994 |
| Previous 15-90 days | | | |  |  |  |  |  |  |
|  | Influenza | 6/275 (2·2%) | 15/1,382 (1·1%) | 2·03 (0·78-5·29) | 0·146 | 2·62 (0·89-7·72) | 0·081 | 2·61 (0·89-7·70) | 0·082 |
|  | URI | 48/275 (17·5%) | 286/1,382 (20·7%) | 0·81 (0·58-1·14) | 0·222 | 0·94 (0·65-1·36) | 0·755 | 0·94 (0·65-1·36) | 0·761 |
| Previous 31-90 days | | | |  |  |  |  |  |  |
|  | Influenza | 6/275 (2·2%) | 14/1,382 (1·0%) | 2·18 (0·83-5·72) | 0·114 | 2·80 (0·94-8·40) | 0·066 | 2·80 (0·93-8·37) | 0·066 |
|  | URI | 42/275 (15·3%) | 236/1,382 (17·1%) | 0·88 (0·61-1·25) | 0·465 | 0·94 (0·64-1·39) | 0·760 | 0·94 (0·64-1·39) | 0·768 |
| The number of medical visits previous 1-365 days (Days, mean, SD) | | | | | |  |  |  |  |
|  | Influenza | 0·051 (0·27) | 0·014 (0·13) | 2·82 (1·49-5·32) | 0·001* | 3·39 (1·60-7·21) | 0·002* | 3·45 (1·62-7·33) | 0·001* |
|  | URI | 1·735 (4·90) | 1·467 (3·57) | 1·02 (0·99-1·05) | 0·299 | 1·03 (0·99-1·06) | 0·117 | 1·03 (1·00-1·06) | 0·100 |

Abbreviations: COPD, Chronic obstructive pulmonary disease; Upper respiratory tract infection, URI; COVID-19, Coronavirus Disease 2019; N/A, Not applicable; SD, Standard deviation

* Unconditional logistic regression model, Significance at P < 0·05

† Model 1 was adjusted for age, sex, income, CCI scores, asthma, COPD, and hypertension

‡ Model 2 was adjusted for model 1 plus influenza and URI
