# Supplementary material for: The associations of previous influenza/upper respiratory infection with COVID-19 susceptibility/morbidity/mortality: a nationwide cohort study in South Korea
Source: Sci Rep. 2021 Nov 3;11:21568. doi: 10.1038/s41598-021-00428-x (PMC8566493; doi:10.1038/s41598-021-00428-x)
Supplement: Supplementary file 8 — Supplementary Information 8. [file 41598_2021_428_MOESM8_ESM.docx]

**Table S8** Crude and adjusted odds ratios of influenza and URI (previous 15-45, 15-90, 31-90, and 1-365 days) for mortality in COVID-19 participants

| Characteristics | | Dead participants | Survived participants | ORs (95% confidence interval) for mortality | | | | | |
| --- | --- | --- | --- | --- | --- | --- | --- | --- | --- |
|  |  | (exposure/total, %) | (exposure/total, %) | Crude | P-value | Model 1† | P-value | Model 2†‡ | P-value |
| **Previous 15-45 days** | | | |  |  |  |  |  |  |
|  | Influenza | 0/237 (0·0%) | 35/7,833 (0·4%) | N/A |  | N/A |  | N/A |  |
|  | URI | 14/237 (5·9%) | 902/7,833 (11·5%) | 0·48 (0·28-0·83) | 0·009* | 0·68 (0·38-1·22) | 0·194 | 0·68 (0·38-1·22) | 0·192 |
| **Previous 15-90 days** | | | |  |  |  |  |  |  |
|  | Influenza | 3/237 (1·3%) | 117/7,833 (1·5%) | 0·85 (0·27-2·68) | 0·776 | 1·09 (0·30-3·93) | 0·896 | 1·09 (0·30-3·94) | 0·891 |
|  | URI | 31/237 (13·1%) | 1,774/7,833 (22·6%) | 0·51 (0·35-0·75) | 0<·001* | 0·71 (0·47-1·09) | 0·114 | 0·71 (0·47-1·09) | 0·114 |
| **Previous 31-90 days** | | | |  |  |  |  |  |  |
|  | Influenza | 3/237 (1·3%) | 109/7,833 (1·4%) | 0·91 (0·29-2·88) | 0·871 | 1·16 (0·32-4·25) | 0·819 | 1·17 (0·32-4·26) | 0·813 |
|  | URI | 28/237 (11·8%) | 1,444/7,833 (18·4%) | 0·59 (0·40-0·88) | 0·010* | 0·77 (0·50-1·20) | 0·252 | 0·77 (0·50-1·20) | 0·252 |
| **The number of medical visit previous 1-365 days (Days, mean, SD)** | | | |  |  |  |  |  |  |
|  | Influenza | 0·038 (0·25) | 0·028 (0·19) | 1·27 (0·72-2·25) | 0·411 | 1·50 (0·72-3·09) | 0·278 | 1·50 (0·72-3·09) | 0·277 |
|  | URI | 1·380 (4·73) | 1·552 (3·06) | 0·98 (0·93-1·03) | 0·398 | 1·01 (0·97-1·05) | 0·651 | 1·01 (0·97-1·05) | 0·648 |

* Unconditional logistic regression model, Significance at P < 0·05

† Model 1 was adjusted for age, sex, income, CCI scores, asthma, COPD, and hypertension

‡ Model 2 was adjusted for model 1 plus influenza and URI
