# Supplementary material for: The associations of previous influenza/upper respiratory infection with COVID-19 susceptibility/morbidity/mortality: a nationwide cohort study in South Korea
Source: Sci Rep. 2021 Nov 3;11:21568. doi: 10.1038/s41598-021-00428-x (PMC8566493; doi:10.1038/s41598-021-00428-x)
Supplement: Supplementary file 9 — Supplementary Information 9. [file 41598_2021_428_MOESM9_ESM.docx]

**Table S9** Subgroup analyses of crude and adjusted odds ratios of influenza and URI (previous 1-14, 1-30, and 1-90 days) for mortality in COVID-19 participants by covariate

| Characteristics | | Dead participants | Survived participants | ORs (95% confidence interval) for mortality | | | | | |
| --- | --- | --- | --- | --- | --- | --- | --- | --- | --- |
|  |  | (exposure/total, %) | (exposure/total, %) | Crude | P-value | Model 1† | P-value | Model 2†‡ | P-value |
| **Age < 50 years old ( n = 4,282)** | | | |  |  |  |  |  |  |
| Previous 1-14 days | | | |  |  |  |  |  |  |
|  | Influenza | 0/4 (0·0%) | 14/4,278 (0·3%) | N/A |  | N/A |  | N/A |  |
|  | URI | 0/4 (0·0%) | 757/4,278 (17·7%) | N/A |  | N/A |  | N/A |  |
| Previous 1-30 days | | | |  |  |  |  |  |  |
|  | Influenza | 0/4 (0·0%) | 16/4,278 (0·4%) | N/A |  | N/A |  | N/A |  |
|  | URI | 0/4 (0·0%) | 938/4,278 (21·9%) | N/A |  | N/A |  | N/A |  |
| Previous 1-90 days | | | |  |  |  |  |  |  |
|  | Influenza | 0/4 (0·0%) | 81/4,278 (1·9%) | N/A |  | N/A |  | N/A |  |
|  | URI | 0/4 (0·0%) | 1,472/4,278 (34·4%) | N/A |  | N/A |  | N/A |  |
| **Age ≥ 50 years old (n = 3,788)** | | | |  |  |  |  |  |  |
| Previous 1-14 days | | | |  |  |  |  |  |  |
|  | Influenza | 2/233 (0·9%) | 11/3,555 (0·3%) | 2·79 (0·62-12·66) | 0·184 | 3·77 (0·72-19·72) | 0·116 | 4·01 (0·76-21·14) | 0·101 |
|  | URI | 27/233 (11·6%) | 657/3,555 (18·5%) | 0·63 (0·38-0·87) | 0·009* | 0·90 (0·58-1·41) | 0·646 | 0·87 (0·56-1·37) | 0·548 |
| Previous 1-30 days | | | |  |  |  |  |  |  |
|  | Influenza | 2/233 (0·9%) | 14/3,555 (0·4%) | 2·19 (0·50-9·70) | 0·301 | 3·09 (0·63-15·29) | 0·167 | 3·32 (0·67-16·45) | 0·142 |
|  | URI | 30/233 (12·9%) | 773/3,555 (21·7%) | 0·53 (0·36-0·79) | 0·002* | 0·86 (0·56-1·32) | 0·490 | 0·84 (0·55-1·29) | 0·423 |
| Previous 1-90 days | | | |  |  |  |  |  |  |
|  | Influenza | 5/233 (2·1%) | 60/3,555 (1·7%) | 1·28 (0·51-3·21) | 0·602 | 1·66 (0·60-4·62) | 0·333 | 1·69 (0·61-4·71) | 0·316 |
|  | URI | 49/233 (21·0%) | 1,173/3,555 (33·0%) | 0·63 (0·38-0·83) | 0·001* | 0·77 (0·54-1·10) | 0·146 | 0·76 (0·53-1·09) | 0·141 |
| **Men (n = 3,236)** | | | |  |  |  |  |  |  |
| Previous 1-14 days | | | |  |  |  |  |  |  |
|  | Influenza | 2/134 (1·5%) | 11/3,102 (0·4%) | 3·90 (0·87-17·61) | 0·077 | 6·92 (1·17-41·15) | 0·033* | 6·63 (1·08-40·61) | 0·041* |
|  | URI | 18/134 (13·4%) | 523/3,102 (16·9%) | 0·79 (0·50-1·34) | 0·417 | 1·16 (0·67-2·02) | 0·598 | 1·08 (0·61-1·90) | 0·792 |
| Previous 1-30 days | | | |  |  |  |  |  |  |
|  | Influenza | 2/134 (1·5%) | 11/3,102 (0·4%) | 4·26 (0·93-19·40) | 0·061 | 6·11 (1·08-34·58) | 0·041* | 6·11 (1·06-35·35) | 0·043* |
|  | URI | 20/134 (14·9%) | 607/3,102 (19·6%) | 0·72 (0·45-1·17) | 0·186 | 1·06 (0·62-1·82) | 0·839 | 1·00 (0·58-1·73) | 0·992 |
| Previous 1-90 days | | | |  |  |  |  |  |  |
|  | Influenza | 4/134 (3·0%) | 59/3,102 (1·9%) | 1·59 (0·57-4·44) | 0·379 | 2·60 (0·82-8·28) | 0·106 | 2·61 (0·82-8·35) | 0·106 |
|  | URI | 30/134 (22·4%) | 941/3,102 (30·3%) | 0·66 (0·44-1·00) | 0·051 | 0·82 (0·51-1·30) | 0·392 | 0·82 (0·51-1·30) | 0·390 |
| **Women (n = 4,834)** | | | |  |  |  |  |  |  |
| Previous 1-14 days | | | |  |  |  |  |  |  |
|  | Influenza | 0/103 (0·0%) | 14/4,731 (0·3%) | N/A |  | N/A |  | N/A |  |
|  | URI | 9/103 (8·7%) | 891/4,731 (18·8%) | 0·41 (0·21-0·82) | 0·012 | 0·65 (0·31-1·35) | 0·250 | 0·65 (0·31-1·36) | 0·252 |
| Previous 1-30 days | | | |  |  |  |  |  |  |
|  | Influenza | 0/103 (0·0%) | 19/4,731 (0·4%) | N/A |  | N/A |  | N/A |  |
|  | URI | 10/103 (9·7%) | 1,104/4,731 (23·3%) | 0·35 (0·18-0·68) | 0·002* | 0·61 (0·30-1·23) | 0·167 | 0·61 (0·30-1·24) | 0·172 |
| Previous 1-90 days | | | |  |  |  |  |  |  |
|  | Influenza | 1/103 (1·0%) | 82/4,731 (1·7%) | 0·56 (0·08-4·03) | 0·561 | 0·68 (0·08-6·09) | 0·728 | 0·74 (0·08-6·57) | 0·788 |
|  | URI | 19/103 (18·4%) | 1,704/4,731 (36·0%) | 0·40 (0·24-0·66) | <0·001* | 0·72 (0·41-1·25) | 0·242 | 0·72 (0·42-1·26) | 0·251 |
| **Low income (n = 2,836)** | | | |  |  |  |  |  |  |
| Previous 1-14 days | | | |  |  |  |  |  |  |
|  | Influenza | 1/86 (1·2%) | 6/2,750 (0·2%) | 5·38 (0·64-45·19) | 0·121 | 11·03 (1·04-116·76) | 0·046* | 15·40 (1·46-162·97) | 0·023* |
|  | URI | 6/86 (7·0%) | 448/2,750 (16·3%) | 0·39 (0·17-0·89) | 0·025* | 0·62 (0·25-1·52) | 0·294 | 0·55 (0·22-1·39) | 0·206 |
| Previous 1-30 days | | | |  |  |  |  |  |  |
|  | Influenza | 1/86 (1·2%) | 11/2,750 (0·4%) | 2·93 (0·37-22·95) | 0·306 | 4·70 (0·52-42·18) | 0·167 | 5·59 (0·62-50·40) | 0·125 |
|  | URI | 6/86 (7·0%) | 557/2,750 (20·3%) | 0·30 (0·13-0·68) | 0·004* | 0·53 (0·22-1·29) | 0·163 | 0·51 (0·21-1·25) | 0·142 |
| Previous 1-90 days | | | |  |  |  |  |  |  |
|  | Influenza | 3/86 (3·5%) | 61/2,750 (2·2%) | 1·59 (0·49-5·18) | 0·439 | 2·18 (0·59-8·05) | 0·241 | 2·17 (0·59-8·05) | 0·247 |
|  | URI | 13/86 (15·1%) | 881/2,750 (32·0%) | 0·38 (0·21-0·69) | 0·001* | 0·69 (0·36-1·35) | 0·282 | 0·70 (0·36-1·36) | 0·287 |
| **Middle income (n = 3,325)** | | | |  |  |  |  |  |  |
| Previous 1-14 days | | | |  |  |  |  |  |  |
|  | Influenza | 0/80 (0·0%) | 12/3,245 (0·4%) | N/A |  | N/A |  | N/A |  |
|  | URI | 11/80 (13·8%) | 644/3,245 (19·8%) | 0·64 (0·34-1·22) | 0·179 | 0·90 (0·44-1·84) | 0·770 | 0·90 (0·44-1·85) | 0·780 |
| Previous 1-30 days | | | |  |  |  |  |  |  |
|  | Influenza | 0/80 (0·0%) | 12/3,245 (0·4%) | N/A | 0·987 | N/A | 0·990 | N/A | 0·991 |
|  | URI | 11/80 (13·8%) | 759/3,245 (23·4%) | 0·52 (0·28-0·99) | 0·047* | 0·81 (0·40-1·66) | 0·566 | 0·81 (0·40-1·67) | 0·574 |
| Previous 1-90 days | | | |  |  |  |  |  |  |
|  | Influenza | 0/80 (0·0%) | 51/3,245 (1·6%) | N/A |  | N/A |  | N/A |  |
|  | URI | 15/80 (18·8%) | 1,145/3,245 (35·3%) | 0·42 (0·24-0·75) | 0·003* | 0·58 (0·31-1·09) | 0·093 | 0·58 (0·31-1·09) | 0·091 |
| **High income (n = 1,909)** | | | |  |  |  |  |  |  |
| Previous 1-14 days | | | |  |  |  |  |  |  |
|  | Influenza | 1/71 (1·4%) | 7/1,838 (0·4%) | 2·90 (0·36-23·23) | 0·315 | 2·80 (0·28-27·78) | 0·379 | 2·58 (0·25-26·53) | 0·424 |
|  | URI | 10/71 (14·1%) | 322/1,838 (17·5%) | 0·81 (0·45-1·66) | 0·659 | 1·30 (0·62-2·71) | 0·489 | 1·26 (0·60-2·65) | 0·542 |
| Previous 1-30 days | | | |  |  |  |  |  |  |
|  | Influenza | 1/71 (1·4%) | 7/1,838 (0·4%) | 3·74 (0·45-30·79) | 0·221 | 2·80 (0·28-27·78) | 0·379 | 2·60 (0·25-26·55) | 0·421 |
|  | URI | 13/71 (18·3%) | 395/1,838 (21·5%) | 0·82 (0·44-1·51) | 0·522 | 1·21 (0·61-2·42) | 0·583 | 1·18 (0·59-2·36) | 0·650 |
| Previous 1-90 days | | | |  |  |  |  |  |  |
|  | Influenza | 2/71 (2·8%) | 29/1,838 (1·6%) | 1·81 (0·42-7·73) | 0·424 | 1·74 (0·33-9·12) | 0·512 | 1·69 (0·32-8·97) | 0·536 |
|  | URI | 21/71 (29·6%) | 619/1,838 (33·7%) | 0·83 (0·49-1·39) | 0·473 | 1·17 (0·65-2·10) | 0·613 | 1·15 (0·64-2·08) | 0·643 |
| **CCI scores = 0 (n = 6,518)** | | | |  |  |  |  |  |  |
| Previous 1-14 days | | | |  |  |  |  |  |  |
|  | Influenza | 1/64 (1·6%) | 21/6,454 (0·3%) | 4·87 (0·65-36·71) | 0·125 | 3·35 (0·27-40·80) | 0·344 | 3·27 (0·26-41·06) | 0·359 |
|  | URI | 13/64 (20·3%) | 1,214/6,454 (18·8%) | 1·10 (0·60-2·03) | 0·760 | 1·09 (0·56-2·12) | 0·800 | 1·05 (0·54-2·06) | 0·880 |
| Previous 1-30 days | | | |  |  |  |  |  |  |
|  | Influenza | 1/64 (1·6%) | 23/6,454 (0·4%) | 4·44 (0·59-33·37) | 0·148 | 3·12 (0·27-36·47) | 0·365 | 3·20 (0·27-37·77) | 0·356 |
|  | URI | 15/64 (23·4%) | 1,480/6,454 (22·9%) | 1·03 (0·58-1·84) | 0·923 | 0·97 (0·51-1·84) | 0·932 | 0·94 (0·50-1·80) | 0·860 |
| Previous 1-90 days | | | |  |  |  |  |  |  |
|  | Influenza | 2/64 (3·1%) | 110/6,454 (1·7%) | 1·86 (0·45-7·70) | 0·392 | 2·63 (0·53-13·19) | 0·240 | 2·82 (0·56-14·11) | 0·207 |
|  | URI | 17/64 (26·6%) | 2,274/6,454 (35·2%) | 0·67 (0·38-1·16) | 0·151 | 0·52 (0·28-0·96) | 0·038* | 0·51 (0·28-0·95) | 0·035* |
| **CCI scores = 1 (n = 889)** | | | |  |  |  |  |  |  |
| Previous 1-14 days | | | |  |  |  |  |  |  |
|  | Influenza | 1/63 (1·6%) | 1/826 (0·1%) | 13·33 (0·82-215·66) | 0·068 | N/A |  | N/A |  |
|  | URI | 7/63 (11·1%) | 140/826 (16·9%) | 0·66 (0·23-1·53) | 0·585 | 0·96 (0·42-2·18) | 0·918 | 0·96 (0·94-0·97) | <0·001* |
| Previous 1-30 days | | | |  |  |  |  |  |  |
|  | Influenza | 1/63 (1·6%) | 1/826 (0·1%) | 13·33 (0·82-215·66) | 0·068 | N/A |  | N/A |  |
|  | URI | 7/63 (11·1%) | 162/826 (19·6%) | 0·51 (0·23-1·15) | 0·103 | 0·72 (0·30-1·71) | 0·458 | 0·77 (0·75-0·78) | <0·001* |
| Previous 1-90 days | | | |  |  |  |  |  |  |
|  | Influenza | 1/63 (1·6%) | 18/826 (2·2%) | 0·73 (0·10-5·51) | 0·756 | 1·48 (0·17-12·86) | 0·722 | 1·41 (0·16-12·31) | 0·759 |
|  | URI | 13/63 (20·6%) | 249/826 (30·1%) | 0·60 (0·32-1·13) | 0·114 | 0·66 (0·33-1·31) | 0·233 | 0·66 (0·33-1·32) | 0·238 |
| **CCI scores ≥ 2 (n = 663)** | | | |  |  |  |  |  |  |
| Previous 1-14 days | | | |  |  |  |  |  |  |
|  | Influenza | 0/110 (0·0%) | 3/553 (0·5%) | N/A |  | N/A |  | N/A |  |
|  | URI | 7/110 (6·4%) | 60/553 (10·8%) | 0·56 (0·25-1·26) | 0·159 | 0·76 (0·32-1·82) | 0·542 | 0·79 (0·33-1·88) | 0·590 |
| Previous 1-30 days | | | |  |  |  |  |  |  |
|  | Influenza | 0/110 (0·0%) | 6/553 (1·1%) | N/A |  | N/A |  | N/A |  |
|  | URI | 8/110 (7·3%) | 69/553 (12·5%) | 0·55 (0·26-1·18) | 0·125 | 0·83 (0·36-1·88) | 0·648 | 0·85 (0·37-1·94) | 0·696 |
| Previous 1-90 days | | | |  |  |  |  |  |  |
|  | Influenza | 2/110 (1·8%) | 13/553 (2·4%) | 0·77 (0·17-3·46) | 0·732 | 1·14 (0·23-5·68) | 0·875 | 1·12 (0·22-5·60) | 0·893 |
|  | URI | 19/110 (17·3%) | 122/553 (22·1%) | 0·74 (0·43-1·26) | 0·264 | 1·17 (0·65-2·11) | 0·609 | 1·17 (0·64-2·11) | 0·613 |
| **Non-asthma (n =7,366 )** | | | |  |  |  |  |  |  |
| Previous 1-14 days | | | |  |  |  |  |  |  |
|  | Influenza | 2/196 (1·0%) | 23/7,170 (0·3%) | 3·20 (0·75-13·68) | 0·116 | 3·71 (0·72-19·13) | 0·117 | 3·76 (0·72-19·62) | 0·117 |
|  | URI | 2/196 (1·0%) | 23/7,170 (0·3%) | 3·20 (0·75-13·68) | 0·116 | 3·71 (0·72-19·13) | 0·117 | 3·76 (0·72-19·62) | 0·117 |
| Previous 1-30 days | | | |  |  |  |  |  |  |
|  | Influenza | 2/196 (1·0%) | 25/7,170 (0·3%) | 2·95 (0·69-12·53) | 0·143 | 2·98 (0·61-14·57) | 0·177 | 3·15 (0·64-15·44) | 0·158 |
|  | URI | 24/196 (12·2%) | 1,537/7,170 (21·4%) | 0·51 (0·33-0·79) | 0·002* | 0·90 (0·56-1·44) | 0·660 | 0·87 (0·54-1·41) | 0·577 |
| Previous 1-90 days | | | |  |  |  |  |  |  |
|  | Influenza | 5/196 (2·6%) | 128/7,170 (1·8%) | 1·44 (0·58-3·56) | 0·430 | 2·30 (0·83-6·38) | 0·111 | 2·36 (0·85-6·54) | 0·100 |
|  | URI | 38/196 (19·4%) | 2,360/7,170 (32·9%) | 0·49 (0·34-0·70) | <0·001* | 0·81 (0·55-1·21) | 0·303 | 0·80 (0·54-1·19) | 0·275 |
| **Asthma (n = 704)** | | | |  |  |  |  |  |  |
| Previous 1-14 days | | | |  |  |  |  |  |  |
|  | Influenza | 0/41 (0·0%) | 2/663 (0·3%) | N/A |  | N/A |  | N/A |  |
|  | URI | 5/41 (12·2%) | 138/663 (20·8%) | 0·53 (0·20-1·37) | 0·190 | 0·64 (0·22-1·87) | 0·417 | 0·64 (0·22-1·87) | 0·417 |
| Previous 1-30 days | | | |  |  |  |  |  |  |
|  | Influenza | 0/41 (0·0%) | 5/663 (0·8%) | N/A |  | N/A |  | N/A |  |
|  | URI | 6/41 (14·6%) | 174/663 (26·2%) | 0·48 (0·20-1·17) | 0·105 | 0·62 (0·23-1·67) | 0·342 | 0·62 (0·23-1·67) | 0·342 |
| Previous 1-90 days | | | |  |  |  |  |  |  |
|  | Influenza | 0/41 (0·0%) | 13/663 (2·0%) | N/A |  | N/A |  | N/A |  |
|  | URI | 11/41 (26·8%) | 285/663 (43·0%) | 0·49 (0·24-0·99) | 0·046* | 0·55 (0·23-1·31) | 0·177 | 0·55 (0·23-1·29) | 0·167 |
| **Non-COPD (n = 7,806)** | | | |  |  |  |  |  |  |
| Previous 1-14 days | | | |  |  |  |  |  |  |
|  | Influenza | 2/206 (1·0%) | 25/7,600 (0·3%) | 2·97 (0·70-12·63) | 0·140 | 3·70 (0·73-18·82) | 0·115 | 3·91 (0·76-20·08) | 0·102 |
|  | URI | 24/206 (11·7%) | 1,372/7,600 (18·1%) | 0·65 (0·41-0·91) | 0·027* | 0·91 (0·57-1·45) | 0·697 | 0·88 (0·55-1·41) | 0·593 |
| Previous 1-30 days | | | |  |  |  |  |  |  |
|  | Influenza | 2/206 (1·0%) | 29/7,600 (0·4%) | 2·56 (0·61-10·80) | 0·201 | 2·99 (0·62-14·45) | 0·174 | 3·24 (0·67-15·71) | 0·145 |
|  | URI | 27/206 (13·1%) | 1,660/7,600 (21·8%) | 0·54 (0·36-0·81) | 0·003* | 0·84 (0·53-1·31) | 0·436 | 0·81 (0·52-1·28) | 0·373 |
| Previous 1-90 days | | | |  |  |  |  |  |  |
|  | Influenza | 5/206 (2·4%) | 132/7,600 (1·7%) | 1·41 (0·57-3·48) | 0·459 | 2·23 (0·81-6·16) | 0·121 | 2·34 (0·85-6·43) | 0·100 |
|  | URI | 42/206 (20·4%) | 2,563/7,600 (33·7%) | 0·50 (0·36-0·71) | <0·001* | 0·71 (0·49-1·04) | 0·082 | 0·70 (0·48-1·03) | 0·072 |
| **COPD (n = 264)** | | | |  |  |  |  |  |  |
| Previous 1-14 days | | | |  |  |  |  |  |  |
|  | Influenza | 0/31 (0·0%) | 0/233 (0·0%) | N/A |  | N/A |  | N/A |  |
|  | URI | 3/31 (9·7%) | 42/233 (18·0%) | 0·49 (0·14-1·68) | 0·255 | 1·02 (0·24-4·40) | 0·980 | 1·02 (0·24-4·40) | 0·980 |
| Previous 1-30 days | | | |  |  |  |  |  |  |
|  | Influenza | 0/31 (0·0%) | 1/233 (0·4%) | N/A |  | N/A |  | N/A |  |
|  | URI | 3/31 (9·7%) | 51/233 (21·9%) | 0·38 (0·11-1·31) | 0·126 | 0·78 (0·19-3·26) | 0·737 | 0·78 (0·19-3·26) | 0·737 |
| Previous 1-90 days | | | |  |  |  |  |  |  |
|  | Influenza | 0/31 (0·0%) | 9/233 (3·9%) | N/A |  | N/A |  | N/A |  |
|  | URI | 7/31 (22·6%) | 82/233 (35·2%) | 0·54 (0·22-1·30) | 0·168 | 1·39 (0·45-4·23) | 0·566 | 1·34 (0·44-4·10) | 0·605 |
| **Non-hypertension (n = 6,413)** | | | |  |  |  |  |  |  |
| Previous 1-14 days | | | |  |  |  |  |  |  |
|  | Influenza | 1/77 (1·3%) | 23/6,336 (0·4%) | 3·61 (0·48-27·09) | 0·212 | 1·80 (0·18-17·81) | 0·616 | 2·08 (0·21-21·05) | 0·535 |
|  | URI | 8/77 (10·4%) | 1,146/6,336 (18·1%) | 0·57 (0·26-1·21) | 0·356 | 0·80 (0·37-1·71) | 0·558 | 0·77 (0·35-1·67) | 0·504 |
| Previous 1-30 days | | | |  |  |  |  |  |  |
|  | Influenza | 1/77 (1·3%) | 26/6,336 (0·4%) | 3·19 (0·43-23·84) | 0·258 | 1·57 (0·17-14·73) | 0·692 | 2·11 (0·23-19·76) | 0·513 |
|  | URI | 8/77 (10·4%) | 1,408/6,336 (22·2%) | 0·41 (0·20-0·85) | 0·016* | 0·55 (0·25-1·22) | 0·143 | 0·53 (0·24-1·20) | 0·127 |
| Previous 1-90 days | | | |  |  |  |  |  |  |
|  | Influenza | 1/77 (1·3%) | 120/6,336 (1·9%) | 0·68 (0·09-4·94) | 0·706 | 0·59 (0·07-4·79) | 0·620 | 0·59 (0·07-4·85) | 0·627 |
|  | URI | 18/77 (23·4%) | 2,165/6,336 (34·2%) | 0·59 (0·35-1·00) | 0·050 | 0·89 (0·49-1·62) | 0·710 | 0·90 (0·50-1·63) | 0·723 |
| **Hypertension (n = 1,657)** | | | |  |  |  |  |  |  |
| Previous 1-14 days | | | |  |  |  |  |  |  |
|  | Influenza | 1/160 (0·6%) | 2/1,497 (0·1%) | 4·68 (0·32-30·30) | 0·324 | 11·33 (1·06-121·32) | 0·045* | 11·39 (1·07-121·50) | 0·044* |
|  | URI | 19/160 (11·9%) | 268/1,497 (17·9%) | 0·62 (0·38-1·02) | 0·058 | 0·97 (0·57-1·67) | 0·915 | 0·96 (0·56-1·65) | 0·880 |
| Previous 1-30 days | | | |  |  |  |  |  |  |
|  | Influenza | 1/160 (0·6%) | 4/1,497 (0·3%) | 2·35 (0·26-21·13) | 0·446 | 7·85 (0·80-77·11) | 0·077 | 7·85 (0·80-77·12) | 0·077 |
|  | URI | 22/160 (13·8%) | 303/1,497 (20·2%) | 0·63 (0·39-1·00) | 0·051 | 1·00 (0·60-1·67) | 0·996 | 1·00 (0·60-1·66) | 0·988 |
| Previous 1-90 days | | | |  |  |  |  |  |  |
|  | Influenza | 4/160 (2·5%) | 21/1,497 (1·4%) | 1·80 (0·61-5·32) | 0·286 | 2·77 (0·78-9·78) | 0·114 | 2·79 (0·79-9·84) | 0·110 |
|  | URI | 31/160 (19·4%) | 480/1,497 (32·1%) | 0·51 (0·34-0·77) | 0·001* | 0·70 (0·45-1·10) | 0·122 | 0·70 (0·45-1·10) | 0·119 |

Abbreviations: COPD, Chronic obstructive pulmonary disease; Upper respiratory tract infection, URI; COVID-19, Coronavirus Disease 2019; N/A, Not applicable; SD, Standard deviation

* Unconditional logistic regression model, Significance at P < 0·05

† Model 1 was adjusted for age, sex, income, CCI scores, asthma, COPD, and hypertension

‡ Model 2 was adjusted for model 1 plus influenza and URI
