# Supplementary material for: The associations of previous influenza/upper respiratory infection with COVID-19 susceptibility/morbidity/mortality: a nationwide cohort study in South Korea
Source: Sci Rep. 2021 Nov 3;11:21568. doi: 10.1038/s41598-021-00428-x (PMC8566493; doi:10.1038/s41598-021-00428-x)
Supplement: Supplementary file 10 — Supplementary Information 10. [file 41598_2021_428_MOESM10_ESM.docx]

**Table S10** Subgroup analyses of crude and adjusted odds ratios of influenza and URI (previous 15-45, 15-90, 31-90, and 1-365 days) for mortality in COVID-19 participants by covariate

| Characteristics | | Dead participants | Survived participants | ORs (95% confidence interval) for mortality | | | | | |
| --- | --- | --- | --- | --- | --- | --- | --- | --- | --- |
|  |  | (exposure/total, %) | (exposure/total, %) | Crude | P-value | Model 1† | P-value | Model 2†‡ | P-value |
| **Age < 50 years old ( n = 4,282)** | | | |  |  |  |  |  |  |
| Previous 15-45 days | | | |  |  |  |  |  |  |
|  | Influenza | 0/4 (0·0%) | 20/4,278 (0·5%) | N/A |  | N/A | 0·976 | N/A |  |
|  | URI | 0/4 (0·0%) | 503/4,278 (11·8%) | N/A |  | N/A | 0·973 | N/A |  |
| Previous 15-90 days | | | |  |  |  |  |  |  |
|  | Influenza | 0/4 (0·0%) | 67/4,278 (1·6%) | N/A |  | N/A | 0·990 | N/A |  |
|  | URI | 0/4 (0·0%) | 1,013/4,278 (23·7%) | N/A |  | N/A | 0·958 | N/A |  |
| Previous 31-90 days | | | |  |  |  |  |  |  |
|  | Influenza | 0/4 (0·0%) | 63/4,278 (1·5%) | N/A |  | N/A | 0·990 | N/A |  |
|  | URI | 0/4 (0·0%) | 829/4,278 (19·4%) | N/A |  | N/A | 0·962 | N/A |  |
| The number of medical visit previous 1-365 days (Days, mean, SD) | | | | | |  |  |  |  |
|  | Influenza | 0·000 (0·00) | 0·034 (0·21) | N/A |  | N/A | 0·982 | N/A |  |
|  | URI | 0·000 (0·00) | 1·605 (2·90) | N/A |  | N/A | 0·800 | N/A |  |
| **Age ≥ 50 years old (n = 3,788)** | | | |  |  |  |  |  |  |
| Previous 15-45 days | | | |  |  |  |  |  |  |
|  | Influenza | 0/233 (0·0%) | 15/3,555 (0·4%) | N/A |  | N/A | 0·994 | N/A |  |
|  | URI | 14/233 (6·0%) | 399/3,555 (11·2%) | 0·51 (0·29-0·88) | 0·015* | 0·69 (0·38-1·25) | 0·221 | 0·69 (0·38-1·25) | 0·218 |
| Previous 15-90 days | | | |  |  |  |  |  |  |
|  | Influenza | 3/233 (1·3%) | 50/3,555 (1·4%) | 0·91 (0·28-2·95) | 0·881 | 1·13 (0·31-4·10) | 0·855 | 1·13 (0·31-4·10) | 0·851 |
|  | URI | 31/233 (13·3%) | 761/3,555 (21·4%) | 0·56 (0·38-0·83) | 0·004* | 0·73 (0·48-1·11) | 0·144 | 0·73 (0·48-1·11) | 0·143 |
| Previous 31-90 days | | | |  |  |  |  |  |  |
|  | Influenza | 3/233 (1·3%) | 46/3,555 (1·3%) | 1·00 (0·31-3·22) | 0·993 | 1·20 (0·33-4·43) | 0·780 | 1·21 (0·33-4·44) | 0·776 |
|  | URI | 28/233 (12·0%) | 615/3,555 (17·3%) | 0·65 (0·44-0·98) | 0·039* | 0·79 (0·51-1·23) | 0·301 | 0·79 (0·51-1·23) | 0·301 |
| The number of medical visit previous 1-365 days (Days, mean, SD) | | | | | |  |  |  |  |
|  | Influenza | 0·039 (0·25) | 0·020 (0·16) | 1·63 (0·90-2·95) | 0·107 | 1·58 (0·74-3·37) | 0·233 | 1·58 (0·75-3·37) | 0·232 |
|  | URI | 1·403 (4·77) | 1·489 (3·25) | 0·99 (0·95-1·04) | 0·706 | 1·01 (0·97-1·05) | 0·593 | 1·01 (0·97-1·05) | 0·590 |
| **Men (n = 3,236)** | | | |  |  |  |  |  |  |
| Previous 15-45 days | | | |  |  |  |  |  |  |
|  | Influenza | 0/134 (0·0%) | 7/3,102 (0·2%) | N/A |  | N/A |  | N/A |  |
|  | URI | 6/134 (4·5%) | 301/3,102 (9·7%) | 0·44 (0·19-1·00) | 0·049* | 0·47 (0·20-1·13) | 0·092 | 0·47 (0·20-1·13) | 0·092 |
| Previous 15-90 days | | | |  |  |  |  |  |  |
|  | Influenza | 2/134 (1·5%) | 48/3,102 (1·5%) | 0·97 (0·23-4·01) | 0·960 | 1·51 (0·32-7·17) | 0·608 | 1·47 (0·31-7·04) | 0·634 |
|  | URI | 17/134 (12·7%) | 605/3,102 (19·5%) | 0·60 (0·36-1·01) | 0·052 | 0·66 (0·37-1·16) | 0·149 | 0·66 (0·37-1·17) | 0·152 |
| Previous 31-90 days | | | |  |  |  |  |  |  |
|  | Influenza | 2/134 (1·5%) | 46/3,102 (1·5%) | 1·01 (0·24-4·19) | 0·993 | 1·55 (0·32-7·44) | 0·582 | 1·52 (0·31-7·30) | 0·605 |
|  | URI | 15/134 (11·2%) | 497/3,102 (16·0%) | 0·66 (0·38-1·14) | 0·136 | 0·70 (0·39-1·29) | 0·254 | 0·71 (0·39-1·29) | 0·259 |
| The number of medical visit previous 1-365 days (Days, mean, SD) | | | | | |  |  |  |  |
|  | Influenza | 0·060 (0·32) | 0·028 (0·19) | 1·73 (0·95-3·13) | 0·072 | 2·65 (1·20-5·88) | 0·017* | 2·65 (1·20-5·88) | 0·016* |
|  | URI | 1·254 (2·57) | 1·309 (2·74) | 0·99 (0·93-1·06) | 0·819 | 1·00 (0·94-1·06) | 0·973 | 1·00 (0·94-1·06) | 0·957 |
| **Women (n = 4,834)** | | | |  |  |  |  |  |  |
| Previous 15-45 days | | | |  |  |  |  |  |  |
|  | Influenza | 0/103 (0·0%) | 28/4,731 (0·6%) | N/A |  | N/A |  | N/A |  |
|  | URI | 8/103 (7·8%) | 601/4,731 (12·7%) | 0·58 (0·28-1·20) | 0·140 | 1·02 (0·46-2·27) | 0·966 | 1·01 (0·46-2·26) | 0·973 |
| Previous 15-90 days | | | |  |  |  |  |  |  |
|  | Influenza | 1/103 (1·0%) | 69/4,731 (1·5%) | 0·66 (0·09-4·82) | 0·684 | 0·75 (0·08-7·02) | 0·801 | 0·79 (0·09-7·27) | 0·832 |
|  | URI | 14/103 (13·6%) | 1,169/4,731 (24·7%) | 0·48 (0·27-0·85) | 0·011* | 0·82 (0·44-1·52) | 0·526 | 0·82 (0·44-1·53) | 0·536 |
| Previous 31-90 days | | | |  |  |  |  |  |  |
|  | Influenza | 1/103 (1·0%) | 63/4,731 (1·3%) | 0·73 (0·10-5·29) | 0·753 | 0·83 (0·09-8·13) | 0·874 | 0·85 (0·09-8·30) | 0·890 |
|  | URI | 13/103 (12·6%) | 947/4,731 (20·0%) | 0·58 (0·32-1·04) | 0·066 | 0·91 (0·48-1·72) | 0·765 | 0·91 (0·48-1·73) | 0·774 |
| The number of medical visit previous 1-365 days (Days, mean, SD) | | | | | |  |  |  |  |
|  | Influenza | 0·010 (0·10) | 0·028 (0·19) | 0·40 (0·06-2·66) | 0·343 | 0·40 (0·05-3·23) | 0·386 | 0·40 (0·05-3·24) | 0·390 |
|  | URI | 1·544 (6·57) | 1·712 (3·25) | 0·98 (0·92-1·05) | 0·611 | 1·02 (0·97-1·07) | 0·400 | 1·02 (0·97-1·07) | 0·405 |
| **Low income (n = 2,836)** | | | |  |  |  |  |  |  |
| Previous 15-45 days | | | |  |  |  |  |  |  |
|  | Influenza | 0/86 (0·0%) | 13/2,750 (0·5%) | N/A |  | N/A |  | N/A |  |
|  | URI | 3/86 (3·5%) | 327/2,750 (11·9%) | 0·27 (0·08-0·85) | 0·026* | 0·59 (0·18-1·98) | 0·393 | 0·58 (0·17-1·96) | 0·383 |
| Previous 15-90 days | | | |  |  |  |  |  |  |
|  | Influenza | 2/86 (2·3%) | 55/2,750 (2·0%) | 1·17 (0·28-4·86) | 0·832 | 1·45 (0·31-6·84) | 0·643 | 1·39 (0·29-6·63) | 0·678 |
|  | URI | 7/86 (8·1%) | 628/2,750 (22·8%) | 0·30 (0·14-0·65) | 0·002* | 0·56 (0·24-1·31) | 0·185 | 0·57 (0·24-1·32) | 0·189 |
| Previous 31-90 days | | | |  |  |  |  |  |  |
|  | Influenza | 2/86 (2·3%) | 51/2,750 (1·9%) | 1·26 (0·30-5·26) | 0·751 | 1·62 (0·34-7·82) | 0·547 | 1·59 (0·33-7·72) | 0·566 |
|  | URI | 6/86 (7·0%) | 501/2,750 (18·2%) | 0·34 (0·15-0·78) | 0·011* | 0·60 (0·24-1·48) | 0·267 | 0·60 (0·24-1·49) | 0·271 |
| The number of medical visit previous 1-365 days (Days, mean, SD) | | | | | |  |  |  |  |
|  | Influenza | 0·058 (0·32) | 0·033 (0·21) | 1·50 (0·71-3·17) | 0·291 | 2·40 (0·99-5·82) | 0·052 | 2·38 (0·98-5·76) | 0·055 |
|  | URI | 0·814 (2·14) | 1·569 (3·46) | 0·85 (0·73-0·97) | 0·019* | 0·96 (0·87-1·07) | 0·485 | 0·97 (0·87-1·07) | 0·506 |
| **Middle income (n = 3,325)** | | | |  |  |  |  |  |  |
| Previous 15-45 days | | | |  |  |  |  |  |  |
|  | Influenza | 0/80 (0·0%) | 16/3,245 (0·5%) | N/A |  | N/A |  | N/A |  |
|  | URI | 3/80 (3·8%) | 373/3,245 (11·5%) | 0·30 (0·09-0·96) | 0·042* | 0·28 (0·08-0·98) | 0·047* | 0·28 (0·08-0·99) | 0·048* |
| Previous 15-90 days | | | |  |  |  |  |  |  |
|  | Influenza | 0/80 (0·0%) | 40/3,245 (1·2%) | N/A |  | N/A |  | N/A |  |
|  | URI | 10/80 (12·5%) | 732/3,245 (22·6%) | 0·49 (0·25-0·96) | 0·037* | 0·65 (0·31-1·36) | 0·253 | 0·65 (0·31-1·36) | 0·250 |
| Previous 31-90 days | | | |  |  |  |  |  |  |
|  | Influenza | 0/80 (0·0%) | 38/3,245 (1·2%) | N/A |  | N/A |  | N/A |  |
|  | URI | 9/80 (11·3%) | 604/3,245 (18·6%) | 0·55 (0·28-1·12) | 0·098 | 0·76 (0·35-1·66) | 0·493 | 0·76 (0·35-1·65) | 0·485 |
| The number of medical visit previous 1-365 days (Days, mean, SD) | | | | | |  |  |  |  |
|  | Influenza | 0·000 (0·00) | 0·025 (0·18) | N/A |  | N/A |  | N/A |  |
|  | URI | 2·038 (7·38) | 1·571 (3·00) | 1·03 (0·98-1·08) | 0·198 | 1·04 (0·99-1·09) | 0·161 | 1·04 (0·99-1·09) | 0·168 |
| **High income (n = 1,909)** | | | |  |  |  |  |  |  |
| Previous 15-45 days | | | |  |  |  |  |  |  |
|  | Influenza | 0/71 (0·0%) | 6/1,838 (0·3%) | N/A |  | N/A |  | N/A |  |
|  | URI | 8/71 (11·3%) | 202/1,838 (11·0%) | 1·03 (0·49-2·18) | 0·941 | 1·33 (0·57-3·11) | 0·509 | 1·34 (0·57-3·13) | 0·504 |
| Previous 15-90 days | | | |  |  |  |  |  |  |
|  | Influenza | 1/71 (1·4%) | 22/1,838 (1·2%) | 1·18 (0·16-8·87) | 0·873 | 1·08 (0·11-10·41) | 0·949 | 1·08 (0·11-10·45) | 0·950 |
|  | URI | 14/71 (1·4%) | 414/1,838 (22·5%) | 0·85 (0·47-1·53) | 0·579 | 1·01 (0·52-1·96) | 0·985 | 1·01 (0·52-1·96) | 0·988 |
| Previous 31-90 days | | | |  |  |  |  |  |  |
|  | Influenza | 1/71 (1·4%) | 20/1,838 (1·1%) | 1·30 (0·17-9·81) | 0·800 | 1·18 (0·12-11·82) | 0·891 | 1·17 (0·12-11·83) | 0·896 |
|  | URI | 13/71 (18·3%) | 339/1,838 (18·4%) | 0·99 (0·54-1·83) | 0·977 | 1·04 (0·52-2·06) | 0·911 | 1·04 (0·52-2·06) | 0·917 |
| The number of medical visit previous 1-365 days (Days, mean, SD) | | | | | |  |  |  |  |
|  | Influenza | 0·056 (0·29) | 0·023 (0·17) | 1·95 (0·81-4·69) | 0·138 | 1·79 (0·53-6·06) | 0·351 | 1·83 (0·53-6·35) | 0·340 |
|  | URI | 1·324 (2·76) | 1·495 (2·48) | 0·97 (0·87-1·08) | 0·571 | 0·98 (0·89-1·07) | 0·636 | 0·98 (0·89-1·07) | 0·609 |
| **CCI scores = 0 (n = 6,518)** | | | |  |  |  |  |  |  |
| Previous 15-45 days | | | |  |  |  |  |  |  |
|  | Influenza | 0/64 (0·0%) | 27/6,454 (0·4%) | N/A |  | N/A |  | N/A |  |
|  | URI | 4/64 (6·3%) | 786/6,454 (12·2%) | 0·48 (0·17-1·33) | 0·157 | 0·38 (0·13-1·13) | 0·082 | 0·38 (0·13-1·13) | 0·082 |
| Previous 15-90 days | | | |  |  |  |  |  |  |
|  | Influenza | 1/64 (1·6%) | 91/6,454 (1·4%) | 1·11 (0·15-8·09) | 0·918 | 2·07 (0·25-17·13) | 0·500 | 1·91 (0·22-16·57) | 0·558 |
|  | URI | 8/64 (12·5%) | 1,530/6,454 (23·7%) | 0·46 (0·22-0·97) | 0·04* | 0·33 (0·14-0·75) | 0·008* | 0·33 (0·14-0·75) | 0·008* |
| Previous 31-90 days | | | |  |  |  |  |  |  |
|  | Influenza | 1/64 (1·6%) | 85/6,454 (1·3%) | 1·19 (0·16-8·68) | 0·864 | 2·28 (0·27-19·22) | 0·447 | 2·08 (0·24-18·27) | 0·510 |
|  | URI | 6/64 (9·4%) | 1,236/6,454 (19·2%) | 0·44 (0·19-1·02) | 0·054 | 0·29 (0·11-0·74) | 0·009* | 0·29 (0·12-0·74) | 0·010* |
| The number of medical visit previous 1-365 days (Days, mean, SD) | | | | | |  |  |  |  |
|  | Influenza | 0·047 (0·28) | 0·027 (0·18) | 1·51 (0·57-4·00) | 0·405 | 2·98 (0·99-8·96) | 0·051 | 3·14 (1·04-9·46) | 0·043* |
|  | URI | 1·344 (2·33) | 1·592 (3·02) | 0·96 (0·87-1·07) | 0·502 | 0·95 (0·85-1·06) | 0·336 | 0·95 (0·85-1·05) | 0·305 |
| **CCI scores = 1 (n = 889)** | | | |  |  |  |  |  |  |
| Previous 15-45 days | | | |  |  |  |  |  |  |
|  | Influenza | 0/63 (0·0%) | 4/826 (0·5%) | N/A |  | N/A |  | N/A |  |
|  | URI | 4/63 (6·3%) | 84/826 (10·2%) | 0·60 (0·21-1·69) | 0·333 | 0·62 (0·21-1·87) | 0·397 | 0·62 (0·21-1·87) | 0·396 |
| Previous 15-90 days | | | |  |  |  |  |  |  |
|  | Influenza | 0/63 (0·0%) | 17/826 (2·1%) | N/A |  | N/A |  | N/A |  |
|  | URI | 8/63 (12·7%) | 163/826 (19·7%) | 0·59 (0·28-1·27) | 0·177 | 0·58 (0·25-1·34) | 0·201 | 0·57 (0·24-1·33) | 0·193 |
| Previous 31-90 days | | | |  |  |  |  |  |  |
|  | Influenza | 0/63 (0·0%) | 17/826 (2·1%) | N/A |  | N/A |  | N/A |  |
|  | URI | 7/63 (11·1%) | 142/826 (17·2%) | 0·60 (0·27-1·35) | 0·218 | 0·55 (0·22-1·35) | 0·193 | 0·54 (0·22-1·34) | 0·185 |
| The number of medical visit previous 1-365 days (Days, mean, SD) | | | | | |  |  |  |  |
|  | Influenza | 0·016 (0·13) | 0·025 (0·17) | 0·64 (0·09-4·55) | 0·656 | 1·32 (0·17-10·18) | 0·793 | 1·33 (0·17-10·31) | 0·784 |
|  | URI | 2·222 (8·38) | 1·574 (3·64) | 1·03 (0·98-1·07) | 0·248 | 1·03 (0·98-1·07) | 0·302 | 1·03 (0·98-1·07) | 0·301 |
| **CCI scores ≥ 2 (n = 663)** | | | |  |  |  |  |  |  |
| Previous 15-45 days | | | |  |  |  |  |  |  |
|  | Influenza | 0/110 (0·0%) | 4/553 (0·7%) | N/A |  | N/A |  | N/A |  |
|  | URI | 6/110 (5·5%) | 32/553 (5·8%) | 0·94 (0·38-2·30) | 0·891 | 1·50 (0·55-4·13) | 0·430 | 1·49 (0·54-4·10) | 0·438 |
| Previous 15-90 days | | | |  |  |  |  |  |  |
|  | Influenza | 2/110 (1·8%) | 9/553 (1·6%) | 1·12 (0·24-5·25) | 0·886 | 1·41 (0·27-7·50) | 0·685 | 1·38 (0·26-7·45) | 0·709 |
|  | URI | 15/110 (13·6%) | 81/553 (14·6%) | 0·92 (0·51-1·67) | 0·783 | 1·48 (0·77-2·86) | 0·240 | 1·48 (0·77-2·85) | 0·245 |
| Previous 31-90 days | | | |  |  |  |  |  |  |
|  | Influenza | 2/110 (1·8%) | 7/553 (1·3%) | 1·45 (0·30-7·05) | 0·649 | 1·60 (0·29-8·92) | 0·592 | 1·51 (0·26-8·71) | 0·643 |
|  | URI | 15/110 (13·6%) | 66/553 (11·9%) | 1·17 (0·64-2·13) | 0·619 | 1·87 (0·96-3·64) | 0·067 | 1·86 (0·95-3·63) | 0·070 |
| The number of medical visit previous 1-365 days (Days, mean, SD) | | | | | |  |  |  |  |
|  | Influenza | 0·045 (0·28) | 0·036 (0·24) | 1·15 (0·54-2·44) | 0·718 | 1·17 (0·48-2·88) | 0·730 | 1·18 (0·48-2·91) | 0·713 |
|  | URI | 0·918 (2·20) | 1·058 (2·53) | 0·98 (0·89-1·07) | 0·590 | 1·03 (0·94-1·13) | 0·564 | 1·03 (0·94-1·13) | 0·555 |
| **Non-asthma (n =7,366 )** | | | |  |  |  |  |  |  |
| Previous 15-45 days | | | |  |  |  |  |  |  |
|  | Influenza | 0/196 (0·0%) | 28/7,170 (0·4%) | N/A |  | N/A |  | N/A |  |
|  | URI | 10/196 (5·1%) | 793/7,170 (11·1%) | 0·43 (0·23-0·82) | 0·01* | 0·65 (0·33-1·30) | 0·225 | 0·66 (0·33-1·31) | 0·230 |
| Previous 15-90 days | | | |  |  |  |  |  |  |
|  | Influenza | 3/196 (1·5%) | 107/7,170 (1·5%) | 1·03 (0·32-3·26) | 0·965 | 1·73 (0·48-6·28) | 0·406 | 1·77 (0·49-6·40) | 0·383 |
|  | URI | 23/196 (11·7%) | 1,561/7,170 (21·8%) | 0·48 (0·31-0·74) | 0·001* | 0·73 (0·45-1·17) | 0·193 | 0·72 (0·45-1·17) | 0·186 |
| Previous 31-90 days | | | |  |  |  |  |  |  |
|  | Influenza | 3/196 (1·5%) | 100/7,170 (1·4%) | 1·10 (0·35-3·50) | 0·873 | 1·93 (0·52-7·16) | 0·325 | 1·96 (0·53-7·24) | 0·312 |
|  | URI | 21/196 (10·7%) | 1,262/7,170 (17·6%) | 0·56 (0·36-0·89) | 0·013* | 0·83 (0·50-1·36) | 0·452 | 0·82 (0·50-1·35) | 0·436 |
| The number of medical visit previous 1-365 days (Days, mean, SD) | | | | | |  |  |  |  |
|  | Influenza | 0·046 (0·27) | 0·026 (0·18) | 1·58 (0·88-2·84) | 0·130 | 1·81 (0·85-3·87) | 0·124 | 1·82 (0·85-3·89) | 0·124 |
|  | URI | 0·934 (2·35) | 1·436 (2·83) | 0·89 (0·82-0·97) | 0·008* | 0·98 (0·91-1·04) | 0·466 | 0·98 (0·91-1·04) | 0·465 |
| **Asthma (n = 704)** | | | |  |  |  |  |  |  |
| Previous 15-45 days | | | |  |  |  |  |  |  |
|  | Influenza | 0/41 (0·0%) | 7/663 (1·1%) | N/A |  | N/A |  | N/A |  |
|  | URI | 4/41 (9·8%) | 109/663 (16·4%) | 0·55 (0·19-1·57) | 0·265 | 0·76 (0·24-2·49) | 0·655 | 0·75 (0·23-2·43) | 0·627 |
| Previous 15-90 days | | | |  |  |  |  |  |  |
|  | Influenza | 0/41 (0·0%) | 10/663 (1·5%) | N/A |  | N/A |  | N/A |  |
|  | URI | 8/41 (19·5%) | 213/663 (32·1%) | 0·51 (0·23-1·13) | 0·097 | 0·60 (0·23-1·56) | 0·294 | 0·58 (0·22-1·51) | 0·267 |
| Previous 31-90 days | | | |  |  |  |  |  |  |
|  | Influenza | 0/41 (0·0%) | 9/663 (1·4%) | N/A |  | N/A |  | N/A |  |
|  | URI | 7/41 (17·1%) | 182/663 (27·5%) | 0·54 (0·24-1·25) | 0·151 | 0·53 (0·19-1·45) | 0·216 | 0·52 (0·19-1·41) | 0·197 |
| The number of medical visit previous 1-365 days (Days, mean, SD) | | | | | |  |  |  |  |
|  | Influenza | 0·000 (0·00) | 0·045 (0·28) | N/A |  | N/A |  | N/A |  |
|  | URI | 3·512 (9·98) | 2·816 (4·76) | 1·02 (0·97-1·07) | 0·410 | 1·04 (0·98-1·10) | 0·210 | 1·04 (0·98-1·10) | 0·214 |
| **Non-COPD (n = 7,806)** | | | |  |  |  |  |  |  |
| Previous 15-45 days | | | |  |  |  |  |  |  |
|  | Influenza | 0/206 (0·0%) | 30/7,600 (0·4%) | N/A |  | N/A |  | N/A |  |
|  | URI | 13/206 (6·3%) | 869/7,600 (11·4%) | 0·52 (0·30-0·92) | 0·024* | 0·72 (0·39-1·33) | 0·295 | 0·72 (0·39-1·34) | 0·300 |
| Previous 15-90 days | | | |  |  |  |  |  |  |
|  | Influenza | 3/206 (1·5%) | 109/7,600 (1·4%) | 1·02 (0·32-3·23) | 0·979 | 1·67 (0·46-6·01) | 0·435 | 1·71 (0·48-6·11) | 0·412 |
|  | URI | 27/206 (13·1%) | 1,713/7,600 (22·5%) | 0·52 (0·35-0·78) | 0·002* | 0·70 (0·45-1·09) | 0·117 | 0·70 (0·45-1·09) | 0·113 |
| Previous 31-90 days | | | |  |  |  |  |  |  |
|  | Influenza | 3/206 (1·5%) | 101/7,600 (1·3%) | 1·10 (0·35-3·49) | 0·875 | 1·85 (0·50-6·79) | 0·355 | 1·89 (0·52-6·87) | 0·337 |
|  | URI | 24/206 (11·7%) | 1,395/7,600 (18·4%) | 0·59 (0·38-0·90) | 0·015* | 0·74 (0·46-1·18) | 0·207 | 0·74 (0·46-1·18) | 0·200 |
| The number of medical visit previous 1-365 days (Days, mean, SD) | | | | | |  |  |  |  |
|  | Influenza | 0·044 (0·27) | 0·027 (0·19) | 1·42 (0·81-2·48) | 0·221 | 1·81 (0·91-3·61) | 0·093 | 1·81 (0·91-3·61) | 0·094 |
|  | URI | 1·413 (5·04) | 1·552 (3·08) | 0·98 (0·93-1·04) | 0·529 | 1·01 (0·97-1·05) | 0·709 | 1·01 (0·97-1·05) | 0·711 |
| **COPD (n = 264)** | | | |  |  |  |  |  |  |
| Previous 15-45 days | | | |  |  |  |  |  |  |
|  | Influenza | 0/31 (0·0%) | 5/233 (2·1%) | N/A |  | N/A |  | N/A |  |
|  | URI | 1/31 (3·2%) | 33/233 (14·2%) | 0·20 (0·03-1·53) | 0·122 | 0·42 (0·05-3·69) | 0·437 | 0·41 (0·05-3·58) | 0·421 |
| Previous 15-90 days | | | |  |  |  |  |  |  |
|  | Influenza | 0/31 (0·0%) | 8/233 (3·4%) | N/A |  | N/A |  | N/A |  |
|  | URI | 4/31 (12·9%) | 61/233 (26·2%) | 0·42 (0·14-1·24) | 0·117 | 0·89 (0·24-3·23) | 0·855 | 0·85 (0·23-3·12) | 0·812 |
| Previous 31-90 days | | | |  |  |  |  |  |  |
|  | Influenza | 0/31 (0·0%) | 8/233 (3·4%) | N/A |  | N/A |  | N/A |  |
|  | URI | 4/31 (12·9%) | 49/233 (21·0%) | 0·56 (0·19-1·67) | 0·295 | 1·24 (0·32-4·76) | 0·757 | 1·19 (0·31-4·59) | 0·799 |
| The number of medical visit previous 1-365 days (Days, mean, SD) | | | | | |  |  |  |  |
|  | Influenza | 0·000 (0·00) | 0·043 (0·20) | N/A |  | N/A |  | N/A |  |
|  | URI | 1·161 (1·63) | 1·567 (2·47) | 0·92 (0·76-1·11) | 0·377 | 1·07 (0·89-1·29) | 0·479 | 1·06 (0·88-1·29) | 0·518 |
| **Non-hypertension (n = 6,413)** | | | |  |  |  |  |  |  |
| Previous 15-45 days | | | |  |  |  |  |  |  |
|  | Influenza | 0/77 (0·0%) | 30/6,336 (0·5%) | N/A |  | N/A |  | N/A |  |
|  | URI | 4/77 (5·2%) | 753/6,336 (11·9%) | 0·41 (0·15-1·12) | 0·080 | 0·59 (0·20-1·71) | 0·331 | 0·59 (0·20-1·72) | 0·332 |
| Previous 15-90 days | | | |  |  |  |  |  |  |
|  | Influenza | 0/77 (0·0%) | 99/6,336 (1·6%) | N/A |  | N/A |  | N/A |  |
|  | URI | 11/77 (14·3%) | 1,460/6,336 (23·0%) | 0·56 (0·29-1·06) | 0·073 | 0·93 (0·46-1·89) | 0·838 | 0·92 (0·45-1·88) | 0·820 |
| Previous 31-90 days | | | |  |  |  |  |  |  |
|  | Influenza | 0/77 (0·0%) | 92/6,336 (1·5%) | N/A |  | N/A |  | N/A |  |
|  | URI | 10/77 (13·0%) | 1,184/6,336 (18·7%) | 0·65 (0·33-1·27) | 0·205 | 1·12 (0·53-2·36) | 0·767 | 1·11 (0·53-2·35) | 0·783 |
| The number of medical visit previous 1-365 days (Days, mean, SD) | | | | | |  |  |  |  |
|  | Influenza | 0·013 (0·11) | 0·030 (0·20) | 0·49 (0·08-3·17) | 0·454 | 0·46 (0·06-3·63) | 0·460 | 0·46 (0·06-3·65) | 0·459 |
|  | URI | 0·844 (1·78) | 1·565 (2·93) | 0·83 (0·71-0·97) | 0·021* | 0·95 (0·83-1·07) | 0·393 | 0·95 (0·83-1·07) | 0·393 |
| **Hypertension (n = 1,657)** | | | |  |  |  |  |  |  |
| Previous 15-45 days | | | |  |  |  |  |  |  |
|  | Influenza | 0/160 (0·0%) | 5/1,497 (0·3%) | N/A |  | N/A |  | N/A |  |
|  | URI | 10/160 (6·3%) | 149/1,497 (10·0%) | 0·60 (0·31-1·17) | 0·135 | 0·76 (0·38-1·55) | 0·449 | 0·76 (0·37-1·54) | 0·441 |
| Previous 15-90 days | | | |  |  |  |  |  |  |
|  | Influenza | 3/160 (1·9%) | 18/1,497 (1·2%) | 1·57 (0·46-5·39) | 0·473 | 1·97 (0·48-8·14) | 0·348 | 1·99 (0·48-8·14) | 0·341 |
|  | URI | 20/160 (12·5%) | 314/1,497 (21·0%) | 0·54 (0·33-0·87) | 0·012* | 0·64 (0·38-1·09) | 0·098 | 0·64 (0·38-1·08) | 0·097 |
| Previous 31-90 days | | | |  |  |  |  |  |  |
|  | Influenza | 3/160 (1·9%) | 17/1,497 (1·1%) | 1·66 (0·48-5·74) | 0·421 | 2·05 (0·49-8·58) | 0·325 | 2·07 (0·50-8·59) | 0·316 |
|  | URI | 18/160 (11·3%) | 260/1,497 (17·4%) | 0·60 (0·36-1·00) | 0·051 | 0·66 (0·38-1·15) | 0·142 | 0·66 (0·38-1·14) | 0·139 |
| The number of medical visit previous 1-365 days (Days, mean, SD) | | | | | |  |  |  |  |
|  | Influenza | 0·050 (0·29) | 0·017 (0·14) | 2·34 (1·15-4·78) | 0·019* | 2·46 (0·99-6·09) | 0·052 | 2·49 (1·00-6·17) | 0·049* |
|  | URI | 1·638 (5·62) | 1·498 (3·58) | 1·01 (0·97-1·05) | 0·660 | 1·02 (0·98-1·06) | 0·317 | 1·02 (0·98-1·06) | 0·294 |

Abbreviations: COPD, Chronic obstructive pulmonary disease; Upper respiratory tract infection, URI; COVID-19, Coronavirus Disease 2019; N/A, Not applicable; SD, Standard deviation

* Unconditional logistic regression model, Significance at P < 0·05

† Model 1 was adjusted for age, sex, income, CCI scores, asthma, COPD, and hypertension

‡ Model 2 was adjusted for model 1 plus influenza and URI
